# Asymptotic Performance of Port-Based Teleportation

**DOI:** 10.1007/s00220-020-03884-0

**Published:** 2020-11-20

**Authors:** Matthias Christandl, Felix Leditzky, Christian Majenz, Graeme Smith, Florian Speelman, Michael Walter

**Affiliations:** 1 QMATH, Department of Mathematical Sciences, University of Copenhagen, Copenhagen, Denmark; 2 JILA, University of Colorado/NIST, Boulder, USA; 3 Center for Theory of Quantum Matter, University of Colorado Boulder, Boulder, CO USA; 4 QuSoft, Amsterdam, The Netherlands; 5 Institute for Logic, Language and Computation, University of Amsterdam, Amsterdam, The Netherlands; 6 Department of Physics, University of Colorado Boulder, Boulder, CO USA; 7 CWI, Amsterdam, The Netherlands; 8 Korteweg-de Vries Institute for Mathematics, University of Amsterdam, Amsterdam, The Netherlands; 9 Institute for Theoretical Physics, University of Amsterdam, Amsterdam, The Netherlands; 10 Informatics Institute, University of Amsterdam, Amsterdam, The Netherlands

## Abstract

Quantum teleportation is one of the fundamental building blocks of quantum Shannon theory. While ordinary teleportation is simple and efficient, port-based teleportation (PBT) enables applications such as universal programmable quantum processors, instantaneous non-local quantum computation and attacks on position-based quantum cryptography. In this work, we determine the fundamental limit on the performance of PBT: for arbitrary fixed input dimension and a large number *N* of ports, the error of the optimal protocol is proportional to the inverse square of *N*. We prove this by deriving an achievability bound, obtained by relating the corresponding optimization problem to the lowest Dirichlet eigenvalue of the Laplacian on the ordered simplex. We also give an improved converse bound of matching order in the number of ports. In addition, we determine the leading-order asymptotics of PBT variants defined in terms of maximally entangled resource states. The proofs of these results rely on connecting recently-derived representation-theoretic formulas to random matrix theory. Along the way, we refine a convergence result for the fluctuations of the Schur–Weyl distribution by Johansson, which might be of independent interest.

## Introduction

### Quantum teleportation protocols

Quantum teleportation [1] is a fundamental quantum information-processing task, and one of the hallmark features of quantum information theory: Two parties Alice and Bob may use a shared entangled quantum state together with classical communication to “teleport” an unknown quantum state from Alice to Bob. The original protocol in [1] consists of Alice measuring the unknown state together with her half of the shared entangled state and letting Bob know about the outcome of her measurement. Based on this information Bob can then manipulate his half of the shared state by applying a suitable correction operation, thus recovering the unknown state in his lab.

From an information-theoretic point of view, quantum teleportation implements a quantum channel between Alice and Bob. If the shared entangled state is a noiseless, maximally entangled state (a so-called EPR state, named after a famous paper by Einstein, Podolski, and Rosen [2]), then this quantum channel is in fact a perfect, noiseless channel. On the other hand, using a noisy entangled state as the shared resource in the teleportation protocol renders the effective quantum channel imperfect or noisy. A common way to measure the noise in a quantum channel is by means of the *entanglement fidelity*, which quantifies how well the channel preserves generic correlations with an inaccessible environment system (see Sect. 2.1 for a definition).

**Fig. 1:**
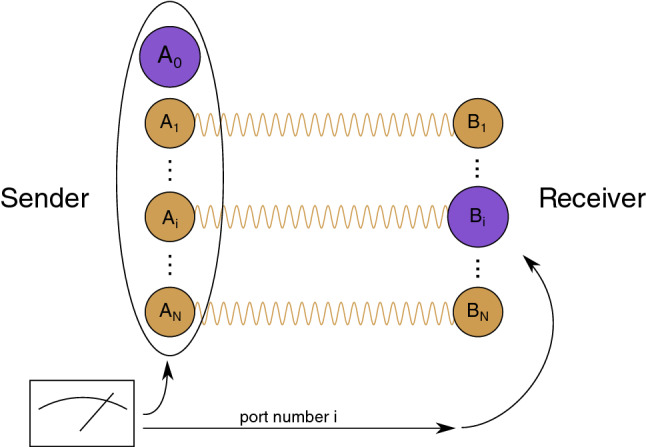
Schematic representation of port-based teleportation (PBT). Like in ordinary teleportation, the sender applies a joint measurement to her input system *A* and her parts of the entangled resource, $A_i, i=1,\ldots ,N$, and sends the outcome to the receiver, who applies a correction operation. In PBT, however, this correction operation merely consists of choosing one of the subsystems $B_i$, the *ports*, of the entangled resource. A PBT protocol cannot implement a perfect quantum channel with a finite number of ports. There are different variants of PBT. The four commonly studied ones are characterized by whether failures are announced, or heralded (probabilistic PBT) or go unnoticed (deterministic PBT), and whether simplifying constraints on the resource state and the sender’s measurement are enforced

Port-based teleportation (PBT) [3, 4] is a variant of the original quantum teleportation protocol [1], where the receiver’s correction operation consists of merely picking the right subsystem, called *port*, of their part of the entangled resource state. Figure 1 provides a schematic description of the protocol (see Sect. 3 for a more detailed explanation). While being far less efficient than the ordinary teleportation protocol, the simple correction operation allows the receiver to apply a quantum operation to the output of the protocol before receiving the classical message. This *simultaneous unitary covariance property* enables all known applications that require PBT instead of just ordinary quantum teleportation, including the construction of universal programmable quantum processors [3], quantum channel discrimination [5] and instantaneous non-local quantum computation (INQC) [6].

In the INQC protocol, which was devised by Beigi and König [6], two spatially separated parties share an input state and wish to perform a joint unitary on it. To do so, they are only allowed a single simultaneous round of communication. INQC provides a generic attack on any quantum position-verification scheme [7], a protocol in the field of position-based cryptography [6, 8–10]. It is therefore of great interest for cryptography to characterize the resource requirements of INQC: it is still open whether a computationally secure quantum position-verification scheme exists, as all known generic attacks require an exponential amount of entanglement. Efficient protocols for INQC are only known for special cases [11–14]. The best lower bounds for the entanglement requirements of INQC are, however, linear in the input size [6, 15, 16], making the hardness of PBT, the corner stone of the best known protocol, the only indication for a possible hardness of INQC.

PBT comes in two variants, deterministic and probabilistic, the latter being distinguished from the former by the fact that the protocol implements a perfect quantum channel whenever it does not fail (errors are “heralded”). In addition, two classes of protocols have been considered in the literature, one using maximally entangled resource states and the other using more complex resources optimized for the protocol. The appeal of the former type of protocol is mostly due to the fact that maximally entangled states are a standard resource in quantum information processing and can be prepared efficiently. Using a protocol based on maximally entangled resources thus removes one parameter from the total complexity of the protocol, the complexity of preparing the resource, leaving the amount of resources as well as the complexity of the involved quantum measurement as the remaining two complexity contributions.

In their seminal work [3, 4], Ishizaka and Hiroshima completely characterize the problem of PBT for qubits. They calculate the performance of the standard^1^ and optimized protocols for deterministic and the EPR and optimized protocols for probabilistic PBT, and prove the optimality of the ‘pretty good’ measurement in the standard deterministic case. They also show a lower bound on the performance of the standard protocol for deterministic PBT, which was later reproven in [6]. Further properties of PBT were explored in [17], in particular with respect to recycling part of the resource state. Converse bounds for the probabilistic and deterministic versions of PBT have been proven in [18] and [19], respectively. In [20], exact formulas for the fidelity of the standard protocol for deterministic PBT with $N=3$ or 4 in arbitrary dimension are derived using a graphical algebra approach. Recently, exact formulas for arbitrary input dimension in terms of representation-theoretic data have been found for all four protocols, and the asymptotics of the optimized probabilistic case have been derived [21, 22].

Note that, in contrast to ordinary teleportation, a protocol obtained from executing several PBT protocols is not again a PBT protocol. This is due to the fact that the whole input system has to be teleported to the same output port for the protocol to have the mentioned simultaneous unitary covariance property. Therefore, the characterization of protocols for any dimension *d* is of particular interest. The mentioned representation-theoretic formulas derived in [21, 22] provide such a characterization. It is, however, not known how to evaluate these formulas efficiently for large input dimension.

### Summary of main results

In this paper we provide several characterization results for port-based teleportation. As our main contributions, we characterize the leading-order asymptotic performance of fully optimized deterministic port-based teleportation (PBT), as well as the standard protocol for deterministic PBT and the EPR protocol for probabilistic PBT. In the following, we provide a detailed summary of our results. These results concern asymptotic characterizations of the entanglement fidelity of deterministic PBT, defined in Sect. 3.1, and the success probability of probabilistic PBT, defined in Sect. 3.2.

Our first, and most fundamental, result concerns deterministic PBT and characterizes the leading-order asymptotics of the optimal fidelity for a large number of ports.

#### Theorem 1.1

For arbitrary but fixed local dimension *d*, the optimal entanglement fidelity for deterministic port-based teleportation behaves asymptotically as

$$\begin{aligned} F_d^*(N) = 1 - \Theta (N^{-2}). \end{aligned}$$

Theorem 1.1 is a direct consequence of Theorem 1.5 below. Prior to our work, it was only known that $F_d^*(N) = 1 - \Omega (N^{-2})$ as a consequence of an explicit converse bound [19]. We prove that this asymptotic scaling is in fact achievable, and we also provide a converse with improved dependency on the local dimension, see Corollary 1.6.

For deterministic port-based teleportation using a maximally entangled resource and the pretty good measurement, a closed expression for the entanglement fidelity was derived in [21], but its asymptotics for fixed $d>2$ and large *N* remained undetermined. As our second result, we derive the asymptotics of deterministic port-based teleportation using a maximally entangled resource and the pretty good measurement, which we call the *standard protocol*.

#### Theorem 1.2

For arbitrary but fixed *d* and any $\delta >0$, the entanglement fidelity of the standard protocol of PBT is given by

$$\begin{aligned} F^{\mathrm {std}}_d(N)=1-\frac{d^2-1}{4N}+O(N^{-\frac{3}{2}+\delta }). \end{aligned}$$

**Fig. 2:**
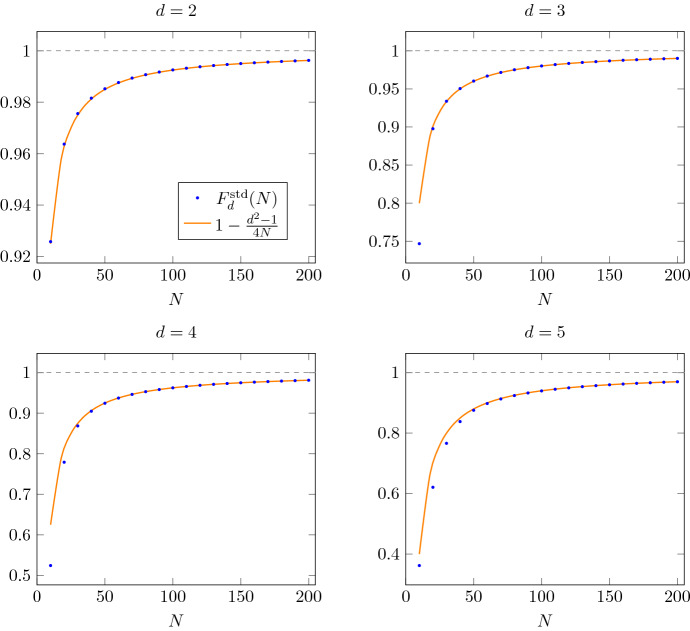
Entanglement fidelity of the standard protocol for deterministic port-based teleportation in local dimension $d=2,3,4,5$ using *N* ports [23]. We compare the exact formula (3.5) for $F_d^{\mathrm {std}}$ (blue dots) with the first-order asymptotics obtained from Theorem 1.2 (orange curve)

Previously, the asymptotic behavior given in the above theorem was only known for $d=2$ in terms of an exact formula for finite *N*; for $d>2$, it was merely known that $F^{\mathrm {std}}_d(N)=1-O\left( N^{-1}\right)$ [4]. In Fig. 2 we compare the asymptotic formula of Theorem 1.2 to a numerical evaluation of the exact formula derived in [21] for $d\le 5$.

For probabilistic port-based teleportation, Mozrzymas et al. [22] obtained the following expression for the success probability $p^*_d$ optimized over arbitrary entangled resources:

$$\begin{aligned} p^*_d(N)=1-\frac{d^2-1}{d^2-1+N}, \end{aligned}$$

valid for all values of *d* and *N* (see the detailed discussion in Sect. 3). In the case of using *N* maximally entangled states as the entangled resource, an exact expression for the success probability in terms of representation-theoretic quantities was also derived in [21]. We state this expression in (3.9) in Sect. 3. However, its asymptotics for fixed $d>2$ and large *N* have remained undetermined to date. As our third result, we derive the following expression for the asymptotics of the success probability of the optimal protocol among the ones that use a maximally entangled resource, which we call the *EPR protocol*.

#### Theorem 1.3

For probabilistic port-based teleportation in arbitrary but fixed dimension *d* with EPR pairs as resource states,

$$\begin{aligned} p^{\mathrm {EPR}}_d(N) = 1 - \sqrt{\frac{d}{N-1}} \mathbb E[\lambda _{\max }(\mathbf {G})] + o\left( N^{-1/2}\right) , \end{aligned}$$

where $$\mathbf {G}\sim {\text {GUE}}^0_d$$.

The famous Wigner semicircle law [24] provides an asymptotic expression for the expected maximal eigenvalue, $\mathbb E[\lambda _{\max }(\mathbf {G})]\sim 2\sqrt{d}$ for $d\rightarrow \infty$. Additionally, there exist explicit upper and lower bounds for all *d*, see the discussion in Sect. 5.

**Fig. 3:**
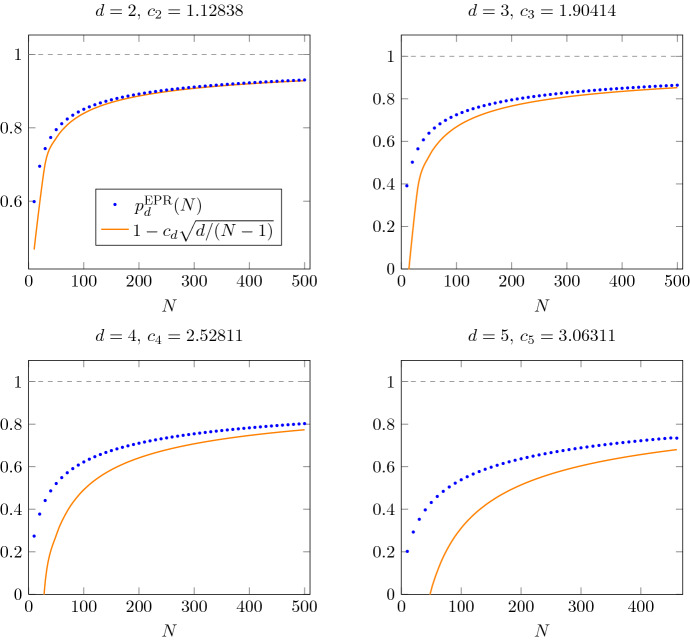
Success probability of the EPR protocol for probabilistic port-based teleporation in local dimension $d=2,3,4,5$ using *N* ports [23]. We compare the exact formula (3.9) for $p_d^{\mathrm {EPR}}$ (blue dots) with the first-order asymptotic formula obtained from Theorem 1.3 (orange curve). The first-order coefficient $c_d \equiv \mathbb {E}[\lambda _{\max }(\mathbf {G})]$ appearing in the formula in Theorem 1.3 was obtained by numerical integration from the eigenvalue distribution of ${\text {GUE}}_d$

To establish Theorems 1.2 and 1.3, we analyze the asymptotics of the Schur–Weyl distribution, which also features in other fundamental problems of quantum information theory including spectrum estimation, tomography, and the quantum marginal problem [25–35]. Our main technical contribution is a new convergence result for its fluctuations that strengthens a previous result by Johansson [36]. This result, which may be of independent interest, is stated as Theorem 4.1 in Sect. 4.

Theorem 1.1 is proved by giving an asymptotic lower bound for the optimal fidelity of deterministic PBT, as well as an upper bound that is valid for any number of ports and matches the lower bound asymptotically. For the lower bound, we again use an expression for the entanglement fidelity of the optimal deterministic PBT protocol derived in [22]. The asymptotics of this formula for fixed *d* and large *N* have remained undetermined so far. We prove an asymptotic lower bound for this entanglement fidelity in terms of the lowest Dirichlet eigenvalue of the Laplacian on the ordered $(d-1)$-dimensional simplex.

#### Theorem 1.4

The optimal fidelity for deterministic port-based teleportation is bounded from below by

$$\begin{aligned} F_d^*(N)&\ge 1-\frac{\lambda _1(\mathrm {OS}_{d-1})}{dN^2}-O(N^{-3}), \end{aligned}$$

where

$$\begin{aligned} \mathrm {OS}_{d-1}=\left\{ x\in \mathbb {R}^d\bigg |\sum \nolimits _i x_i=1, x_i\ge x_{i+1}, x_d\ge 0\right\} \end{aligned}$$

is the $(d-1)$-dimensional simplex of ordered probability distributions with *d* outcomes and $\lambda _1(\Omega )$ is the first eigenvalue of the Dirichlet Laplacian on a domain $\Omega$.

Using a bound from [37] for $\lambda _1(\mathrm {OS}_d)$, we obtain the following explicit lower bound.

#### Theorem 1.5

For the optimal fidelity of port-based teleportation with arbitrary but fixed input dimension *d* and *N* ports, the following bound holds,

$$\begin{aligned} F^*_d(N)\ge 1-\frac{ d^5+O(d^{9/2})}{4\sqrt{2} N^2}+O(N^{-3}). \end{aligned}$$

As a complementary result, we give a strong upper bound for the entanglement fidelity of any deterministic port-based teleportation protocol. While valid for any finite number *N* of ports, its asymptotics for large *N* are given by $1-O(N^{-2})$, matching Theorem 1.5.

#### Corollary 1.6

For a general port-based teleportation scheme with input dimension *d* and *N* ports, the entanglement fidelity $F_d^*$ and the diamond norm error $\varepsilon _d^*$ can be bounded as

$$\begin{aligned} F_d^*(N)&\le {\left\{ \begin{array}{ll} \frac{\sqrt{N}}{d}&{}\quad \text { if } N\le \frac{d^2}{2}\\ 1-\frac{d^2-1}{16N^2}&{}\quad \text { otherwise} \end{array}\right. }&\varepsilon _d^*(N)\ge {\left\{ \begin{array}{ll} 2\bigl (1-\frac{\sqrt{N}}{d}\bigr )&{}\quad \text { if } N\le \frac{d^2}{2}\\ 2\frac{d^2-1}{16N^2}&{}\quad \text { otherwise.} \end{array}\right. } \end{aligned}$$

Previously, the best known upper bound on the fidelity [19] had the same dependence on *N*, but was increasing in *d*, thus failing to reflect the fact that the task becomes harder with increasing *d*. Interestingly, a lower bound from [38] on the program register size of a universal programmable quantum processor also yields a converse bound for PBT that is incomparable to the one from [19] and weaker than our bound.

Finally we provide a proof of the following ‘folklore’ fact that had been used in previous works on port-based teleportation. The unitary and permutation symmetries of port-based teleportation imply that the entangled resource state and Alice’s measurement can be chosen to have these symmetries as well. Apart from simplifying the optimization over resource states and POVMs, this implies that characterizing the entanglement fidelity is sufficient to give worst-case error guarantees. Importantly, this retrospectively justifies the use of the entanglement fidelity *F* in the literature about deterministic port-based teleportation in the sense that any bound on *F* implies a bound on the diamond norm error without losing dimension factors. This is also used to show the diamond norm statement of Corollary 1.6.

#### Proposition 1.7

(Proposition 3.4 and 3.3 and Corollary 3.5, informal). There is an explicit transformation between port-based teleportation protocols that preserves any unitarily invariant distance measure on quantum channels, and maps an arbitrary port-based teleportation protocol with input dimension *d* and *N* ports to a protocol that

(i) has a resource state and a POVM with $U(d)\times S_N$ symmetry, and
(ii) implements a unitarily covariant channel.

In particular, the transformation maps an arbitrary port-based teleportation protocol to one with the symmetries (i) and (ii) above, and entanglement fidelity no worse than the original protocol. Point (ii) implies that

$$\begin{aligned} \varepsilon _d^*=2\left( 1-F_d^*\right) , \end{aligned}$$

where $F_d^*$ and $\varepsilon _d^*$ denote the optimal entanglement fidelity and optimal diamond norm error for deterministic port-based teleportation.

### Structure of this paper

In Sect. 2 we fix our notation and conventions and recall some basic facts about the representation theory of the symmetric and unitary groups. In Sect. 3 we define the task of port-based teleportation (PBT) in its two main variants, the probabilistic and deterministic setting. Moreover, we identify the inherent symmetries of PBT, and describe a representation-theoretic characterization of the task. In Sect. 4 we discuss the Schur–Weyl distribution and prove a convergence result that will be needed to establish our results for PBT with maximally entangled resources. Our first main result is proved in Sect. 5, where we discuss the probabilistic setting in arbitrary dimension using EPR pairs as ports, and determine the asymptotics of the success probability $p^{\mathrm {EPR}}_d$ (Theorem 1.3). Our second main result, derived in Sect. 6.1, concerns the deterministic setting in arbitrary dimension using EPR pairs, for which we compute the asymptotics of the optimal entanglement fidelity $F^{\mathrm {std}}_d$ (Theorem 1.2). Our third result, an asymptotic lower bound on the entanglement fidelity $F_d^*$ of the optimal protocol in the deterministic setting (Theorem 1.5), is proved in Sect. 6.2. Finally, in Sect. 7 we derive a general non-asymptotic converse bound on deterministic port-based teleportation protocols using a non-signaling argument (Theorem 7.5). We also present a lower bound on the communication requirements for approximate quantum teleportation (Corollary 7.4). We make some concluding remarks in Sect. 8. The appendices contain technical proofs.

## Preliminaries

### Notation and definitions

We denote by *A*, *B*, ...quantum systems with associated Hilbert spaces $\mathcal {H}_A$, $\mathcal {H}_B$, ..., which we always take to be finite-dimensional, and we associate to a multipartite quantum system $A_1\ldots A_n$ the Hilbert space $\mathcal {H}_{A_1\ldots {}A_n}=\mathcal {H}_{A_1}\otimes \ldots \otimes \mathcal {H}_{A_n}$. When the $A_i$ are identical, we also write $A^n=A_1\ldots {}A_n$. The set of linear operators on a Hilbert space $\mathcal {H}$ is denoted by $\mathcal {B}(\mathcal {H})$. A *quantum state* $\rho _A$ on quantum system *A* is a positive semidefinite linear operator $\rho _A\in \mathcal {B}(\mathcal {H}_A)$ with unit trace, i.e., $\rho _A\ge 0$ and ${{\,\mathrm{tr}\,}}(\rho _A)=1$. We denote by $I_A$ or $1_A$ the identity operator on $\mathcal {H}_A$, and by $\tau _A=I_A/|A|$ the corresponding *maximally mixed* quantum state, where $|A|\,{:}{=}\,\dim \mathcal {H}_A$. A pure quantum state $\psi _A$ is a quantum state of rank one. We can write $\psi _A=|\psi \rangle \langle \psi |_A$ for a unit vector $|\psi \rangle _A\in \mathcal {H}_A$. For quantum systems $A,A'$ of dimension $\dim \mathcal {H}_A=\dim \mathcal {H}_{A'}=d$ with bases $\lbrace |i\rangle _A\rbrace _{i=1}^d$ and $\lbrace |i\rangle _{A'}\rbrace _{i=1}^d$, the vector $|\phi ^+\rangle _{A'A} = \frac{1}{\sqrt{d}} \sum _{i=1}^d |i\rangle _{A'}\otimes |i\rangle _A$ defines the *maximally entangled* state of Schmidt rank *d*. The *fidelity* $F(\rho ,\sigma )$ between two quantum states is defined by $F(\rho ,\sigma )\,{:}{=}\, \Vert \sqrt{\rho }\sqrt{\sigma }\Vert _1^2$, where $\Vert X\Vert _1={{\,\mathrm{tr}\,}}(\sqrt{X^\dagger X})$ denotes the *trace norm* of an operator. For two pure states $|\psi \rangle$ and $|\phi \rangle$, the fidelity is equal to $F(\psi ,\phi )=|\langle \psi |\phi \rangle |^2$. A *quantum channel* is a completely positive, trace-preserving linear map $\Lambda :\mathcal {B}(\mathcal {H}_A)\rightarrow \mathcal {B}(\mathcal {H}_B)$. We also use the notation $\Lambda :A\rightarrow B$ or $\Lambda _{A\rightarrow B}$, and we denote by ${{\,\mathrm{id}\,}}_A$ the identity channel on *A*. Given two quantum channels $\Lambda _1,\Lambda _2:A\rightarrow B$, the *entanglement fidelity* $F(\Lambda _1,\Lambda _2)$ is defined as

$$\begin{aligned} F(\Lambda _1,\Lambda _2) \,{:}{=}\, F(({{\,\mathrm{id}\,}}_{A'}\otimes \Lambda _1)(\phi ^+_{A'A}),({{\,\mathrm{id}\,}}_{A'}\otimes \Lambda _2)(\phi ^+_{A'A})), \end{aligned}$$

and we abbreviate $F(\Lambda )\,{:}{=}\, F(\Lambda ,{{\,\mathrm{id}\,}})$. The *diamond norm* of a linear map $\Lambda :\mathcal {B}(\mathcal {H}_A)\rightarrow \mathcal {B}(\mathcal {H}_B)$ is defined by

$$\begin{aligned} \Vert \Lambda \Vert _\diamond \,{:}{=}\, \sup _{\Vert X_{A'A}\Vert _1\le 1}\Vert ({{\,\mathrm{id}\,}}_{A'}\otimes \Lambda )(X_{A'A}) \Vert _1. \end{aligned}$$

The induced distance on quantum channels is called the *diamond distance*. A *positive operator-valued measure (POVM)* $E=\lbrace E_x\rbrace$ on a quantum system *A* is a collection of positive semidefinite operators $E_x\ge 0$ satisfying $\sum _x E_x = I_A$.

We denote random variables by bold letters ($\mathbf {X}$, $\mathbf {Y}$, $\mathbf {Z}$, ...) and the valued they take by the non-bold versions ($X,Y,Z,\ldots$). We denote by $\mathbf {X} \sim \mathbb {P}$ that $\mathbf {X}$ is a random variable with probability distribution $\mathbb {P}$. We write $\Pr (\ldots )$ for the probability of an event and $\mathbb {E}\left[ \ldots \right]$ for expectation values. The notation $\mathbf {X}_n\overset{P}{\rightarrow }\mathbf {X} \ (n\rightarrow \infty )$ denotes *convergence in probability* and $\mathbf {X}_n\overset{D}{\rightarrow }\mathbf {X} \ (n\rightarrow \infty )$ denotes *convergence in distribution*. The latter can be defined, e.g., by demanding that $\mathbb {E}\left[ f(\mathbf {X}_n)\right] \rightarrow \mathbb {E}\left[ f(\mathbf {X})\right] \ (n\rightarrow \infty )$ for every continuous, bounded function *f*. The *Gaussian unitary ensemble* ${\text {GUE}}_d$ is the probability distribution on the set of Hermitian $d\times d$-matrices *H* with density $Z_d^{-1} \exp (-\frac{1}{2}{{\,\mathrm{tr}\,}}H^2)$, where $Z_d$ is the appropriate normalization constant. Alternatively, for $\mathbf {X}\sim {\text {GUE}}_d$, the entries $\mathbf {X}_{ii}$ for $1\le i\le d$ are independently distributed as $\mathbf {X}_{ii}\sim N(0,1)$, whereas the elements $\mathbf {X}_{ij}$ for $1\le i<j\le d$ are independently distributed as $\mathbf {X}_{ij}\sim N(0,\frac{1}{2})+iN(0,\frac{1}{2})$. Here, $N(0,\sigma ^2)$ denotes the centered normal distribution with variance $\sigma ^2$. The *traceless Gaussian unitary ensemble* ${\text {GUE}}^0_d$ can be defined as the distribution of the random variable $\mathbf {Y} \,{:}{=}\, \mathbf {X} - \tfrac{{{\,\mathrm{tr}\,}}\mathbf {X}}{d} I$, where $\mathbf {X}\sim {\text {GUE}}_d$.

For a complex number $z\in \mathbb {C}$, we denote by $\mathfrak {R}( z)$ and $\mathfrak {I}( z)$ its real and imaginary part, respectively. We denote by $\mu \vdash _d n$ a partition $(\mu _1,\ldots ,\mu _d)$ of *n* into *d* parts. That is, $\mu \in \mathbb {Z}^d$ with $\mu _1\ge \mu _2\ge \cdots \ge \mu _d\ge 0$ and $\sum _i\mu _i=n$. We also call $\mu$ a *Young diagram* and visualize it as an arrangement of boxes, with $\mu _i$ boxes in the *i*th row. For example, $\mu =(3,1)$ can be visualized as . We use the notation $(i,j)\in \mu$ to mean that (*i*, *j*) is a box in the Young diagram $\mu$, that is, $1\le i\le d$ and $1\le j\le \mu _i$. We denote by ${{\,\mathrm{GL}\,}}(\mathcal {H})$ the general linear group and by $U(\mathcal {H})$ the unitary group acting on a Hilbert space $\mathcal {H}$. When $\mathcal {H}=\mathbb {C}^d$, we write ${{\,\mathrm{GL}\,}}(d)$ and *U*(*d*). Furthermore, we denote by $S_n$ the symmetric group on *n* symbols. A *representation* $\varphi$ of a group *G* on a vector space $\mathcal {H}$ is a map $G\ni g\mapsto \varphi (g)\in {{\,\mathrm{GL}\,}}(\mathcal {H})$ satisfying $\varphi (gh)=\varphi (g)\varphi (h)$ for all $g,h\in G$. In this paper all representations are unitary, which means that $\mathcal {H}$ is a Hilbert space and $\varphi (g) \in U(\mathcal {H})$ for every $g\in G$. A representation is irreducible (or an *irrep*) if $\mathcal {H}$ contains no nontrivial invariant subspace.

### Representation theory of the symmetric and unitary group

Our results rely on the representation theory of the symmetric and unitary groups and Schur–Weyl duality (as well as their semiclassical asymptotics which we discuss in Sect. 4). In this section we introduce the relevant concepts and results (see e.g., [39, 40].

The irreducible representations of $S_n$ are known as *Specht modules* and labeled by Young diagrams with *n* boxes. We denote the Specht module of $S_n$ corresponding to a Young diagram $\mu \vdash _d n$ by $[\mu ]$ (*d* is arbitrary). Its dimension is given by the *hook length formula* [39, pp. 53–54],

2.1 $$\begin{aligned} d_\mu =\frac{n!}{\prod _{(i,j)\in \mu }h_\mu (i,j)}, \end{aligned}$$

where $h_\mu (i,j)$ is the *hook length* of the hook with corner at the box (*i*, *j*), i.e., the number of boxes below (*i*, *j*) plus the number of boxes to the right of (*i*, *j*) plus one (the box itself).

The polynomial irreducible representations of *U*(*d*) are known as *Weyl modules* and labeled by Young diagrams with no more than *d* rows. We denote the Weyl module of *U*(*d*) corresponding to a Young diagram $\mu \vdash _d n$ by $V^d_\mu$ (*n* is arbitrary). Its dimension can be computed using *Stanley’s hook length formula* [39, p. 55],

2.2 $$\begin{aligned} m_{d,\mu }=\prod _{(i,j)\in \mu } \frac{d+c(i,j)}{h_\mu (i,j)}, \end{aligned}$$

where $c(i,j) = j-i$ is the so-called *content* of the box (*i*, *j*). This is an alternative to the *Weyl dimension formula*, which states that

2.3 $$\begin{aligned} m_{d,\mu }=\prod _{1\le i<j\le d} \frac{\mu _i - \mu _j + j - i}{j - i}. \end{aligned}$$

We stress that $m_{d,\mu }$ depends on the dimension *d*.

Consider the representations of $S_n$ and *U*(*d*) on ${\left( \mathbb {C}^d\right) }^{\otimes n}$ given by permuting the tensor factors, and multiplication by $U^{\otimes n}$, respectively. Clearly the two actions commute. *Schur–Weyl duality* asserts that the decomposition of ${\left( \mathbb {C}^d\right) }^{\otimes n}$ into irreps takes the form (see, e.g., [40])

2.4 $$\begin{aligned} {\left( \mathbb {C}^d\right) }^{\otimes n} \cong \bigoplus _{\mu \vdash _d n} [\mu ] \otimes V^d_\mu . \end{aligned}$$

## Port-Based Teleportation

The original quantum teleportation protocol for qubits (henceforth referred to as ordinary teleportation protocol) is broadly described as follows [1]: Alice (the sender) and Bob (the receiver) share an EPR pair (a maximally entangled state on two qubits), and their goal is to transfer or ‘teleport’ another qubit in Alice’s possession to Bob by sending only classical information. Alice first performs a joint Bell measurement on the quantum system to be teleported and her share of the EPR pair, and communicates the classical measurement outcome to Bob using two bits of classical communication. Conditioned on this classical message, Bob then executes a correction operation consisting of one of the Pauli operators on his share of the EPR pair. After the correction operation, he has successfully received Alice’s state. The ordinary teleportation protocol can readily be generalized to qudits, i.e., *d*-dimensional quantum systems. Note that while the term ‘EPR pair’ is usually reserved for a maximally entangled state on two qubits ($d=2$), we use the term more freely for maximally entangled states of Schmidt rank *d* on two qudits, as defined in Sect. 2.

Port-based teleportation, introduced by Ishizaka and Hiroshima [3, 4], is a variant of quantum teleportation where Bob’s correction operation solely consists of picking one of a number of quantum subsystems upon receiving the classical message from Alice. In more detail, Alice and Bob initially share an entangled resource quantum state $\psi _{A^N B^N}$, where $\mathcal {H}_{A_i}\cong \mathcal {H}_{B_i}\cong \mathbb {C}^d$ for $i=1,\ldots ,N$. We may always assume that the resource state is pure, for we can give a purification to Alice and she can choose not to use it.^2^ Bob’s quantum systems $B_i$ are called *ports*. Just like in ordinary teleportation, the goal is for Alice to teleport a *d*-dimensional quantum system $A_0$ to Bob. To achieve this, Alice performs a joint POVM $\{(E_i)_{A_0A^N}\}_{i=1}^N$ on the input and her part of the resource state and sends the outcome *i* to Bob. Based on the index *i* he receives, Bob selects the *i*th port, i.e. the system $B_i$, as being the output register (renaming it to $B_0$), and discards the rest. That is, in contrast to ordinary teleportation, Bob’s decoding operation solely consists of selecting the correct port $B_i$. The quality of the teleportation protocol is measured by how well it simulates the identity channel from Alice’s input register $A_0$ to Bob’s output register $B_0$.

Port-based teleportation is impossible to achieve perfectly with finite resources [3], a fact first deduced from the application to universal programmable quantum processors [41]. There are two ways to deal with this fact: either one can just accept an imperfect protocol, or one can insist on simulating a perfect identity channel, with the caveat that the protocol will fail from time to time. This leads to two variants of PBT, which are called *deterministic* and *probabilistic* PBT in the literature [3].^3^

### Deterministic PBT

A protocol for deterministic PBT proceeds as described above, implementing an imperfect simulation of the identity channel whose merit is quantified by the entanglement fidelity $F_d$ or the diamond norm error $\varepsilon _d$. We denote by $F_d^*(N)$ and $\varepsilon _d^*(N)$ the maximal entanglement fidelity and the minimal diamond norm error for deterministic PBT, respectively, where both the resource state and the POVM are optimized. We will often refer to this as the *fully optimized* case.

Let $\psi _{{A}^N{B}^N}$ be the entangled resource state used for a PBT protocol. When using the entanglement fidelity as a figure of merit, it is shown in [4] that the problem of PBT for the fixed resource state $\psi _{{A}^N{B}^N}$ is equivalent to the state discrimination problem given by the collection of states

3.1 $$\begin{aligned} \eta ^{(i)}_{A^NB_0}=\mathrm {id}_{B_i\rightarrow B_0}{{\,\mathrm{tr}\,}}_{B_i^c}|\psi \rangle \langle \psi |_{{A}^N{B}^N}, \qquad i=1,\ldots ,N. \end{aligned}$$

with uniform prior (here we trace over all *B* systems but $B_i$, which is relabeled to $B_0$). More precisely, the success probability *q* for state discrimination with some fixed POVM $\{E_i\}_{i=1}^N$ and the entanglement fidelity $F_d$ of the PBT protocol with Alice’s POVM equal to $\{E_i\}_{i=1}^N$, but acting on $A^NA_0$, are related by the equation $q=\frac{d^2}{N}F_d$. This link with state discrimination provides us with the machinery developed for state discrimination to optimize the POVM. In particular, it suggests the use of the pretty good measurement [43, 44].

As in ordinary teleportation, it is natural to consider PBT protocols where the resource state is fixed to be *N* maximally entangled states (or EPR pairs) of local dimension *d*. This is because EPR pairs are a standard resource in quantum information theory that can easily be produced in a laboratory. We will denote by $F_d^{\mathrm {EPR}}(N)$ the optimal entanglement fidelity for any protocol for deterministic PBT that uses maximally entangled resource states. A particular protocol is given by combining maximally entangled resource states with the pretty good measurement (PGM) POVM [43, 44]. We call this the *standard protocol* for deterministic PBT and denote the corresponding entanglement fidelity by $F^{\mathrm {std}}_d(N)$. For qubits ($d=2$), the pretty good measurement was shown to be optimal for maximally entangled resource states [4]:

3.2 $$\begin{aligned} F^{\mathrm {std}}_{2}(N) = F^{\mathrm {EPR}}_{2}(N) = 1 - \frac{3}{4N} + o(1/N). \end{aligned}$$

According to [22], the PGM is optimal in this situation for $d>2$ as well.

In [3] it is shown that the entanglement fidelity $F^{\mathrm {std}}_d$ for the standard protocol is at least

3.3 $$\begin{aligned} F^{\mathrm {std}}_d(N) \ge 1-\frac{d^2-1}{N}. \end{aligned}$$

Beigi and König [6] rederived the same bound with different techniques. In [19], a converse bound is provided in the fully optimized setting:

3.4 $$\begin{aligned} F^*_d(N) \le 1-\frac{1}{4(d-1)N^2}+ O(N^{-3}). \end{aligned}$$

Note that the dimension *d* is part of the denominator instead of the numerator as one might expect in the asymptotic setting. Thus, the bound lacks the right qualitative behavior for large values of *d*. A different, incomparable, bound can be obtained from a recent lower bound on the program register dimension of a universal programmable quantum processor obtained by Kubicki et al. [38],

$$\begin{aligned} \varepsilon ^*_d(N) \ge 2\left( 1-c \frac{\log d}{d}\left( 2N+\frac{2}{3}\right) \right) , \end{aligned}$$

where *c* is a constant. By Proposition 1.7, this bound is equivalent to

$$\begin{aligned} F^*_d(N)\le c \frac{\log d}{d}\left( 2N+\frac{2}{3}\right) . \end{aligned}$$

Earlier works on programmable quantum processors [45, 46] also yield (weaker) converse bounds for PBT.

Interestingly, and of direct relevance to our work, *exact* formulas for the entanglement fidelity have been derived both for the standard protocol and in the fully optimized case. In [21], the authors showed that

3.5 $$\begin{aligned} F^{\mathrm {std}}_d(N)=d^{-N-2}\sum _{\alpha \vdash _d N-1}\left( \sum _{\mu =\alpha +\square }\sqrt{d_\mu m_{d,\mu }}\right) ^2. \end{aligned}$$

Here, the inner sum is taken over all Young diagrams $\mu$ that can be obtained by adding one box to a Young diagram $\alpha \vdash _d N-1$, i.e., a Young diagram with $N-1$ boxes and at most *d* rows. Equation (3.5) generalizes the result of [4] for $d=2$, whose asymptotic behavior is stated in Eq. (3.2).

In the fully optimized case, Mozrzymas et al. [22] obtained a formula similar to Eq. (3.5) in which the dimension $d_\mu m_{d,\mu }$ of the $\mu$-isotypic component in the Schur–Weyl decomposition is weighted by a coefficient $c_\mu$ that is optimized over all probability densities with respect to the Schur–Weyl distribution (defined in Sect. 4). More precisely,

3.6 $$\begin{aligned} F^{*}_d(N) =d^{-N-2}\max _{c_\mu }\sum _{\alpha \vdash _d N-1}\left( \sum _{\mu =\alpha +\square }\sqrt{c_\mu d_\mu m_{d,\mu }}\right) ^2, \end{aligned}$$

where the optimization is over all nonnegative coefficients $\{c_\mu \}$ such that $\sum _{\mu \vdash _d N} c_\mu \frac{d_\mu m_{d,\mu }}{d^N}=1$.

### Probabilistic PBT

In the task of probabilistic PBT, Alice’s POVM has an additional outcome that indicates the failure of the protocol and occurs with probability $1-p_d$. For all other outcomes, the protocol is required to simulate the identity channel perfectly. We call $p_d$ the probability of success of the protocol. As before, we denote by $p_d^*(N)$ the maximal probability of success for probabilistic PBT using *N* ports of local dimension *d*, where the resource state as well as the POVM are optimized.

Based on the no-signaling principle and a version of the no-cloning theorem, Pitalúa-García [18] showed that the success probability $p^*_{2^n}(N)$ of teleporting an *n*-qubit input state using a general probabilistic PBT protocol is at most

3.7 $$\begin{aligned} p^*_{2^n}(N) \le 1-\frac{4^n-1}{4^n-1+N}. \end{aligned}$$

Subsequently, Mozrzymas et al. [22] showed for a general *d*-dimensional input state that the converse bound in (3.7) is also achievable, establishing that

3.8 $$\begin{aligned} p^*_d(N)=1-\frac{d^2-1}{d^2-1+N}. \end{aligned}$$

This fully resolves the problem of determining the optimal probability of success for probabilistic PBT in the fully optimized setting.

As discussed above, it is natural to also consider the scenario where the resource state is fixed to be *N* maximally entangled states of rank *d* and consider the optimal POVM given that resource state. We denote by $p^{\mathrm {EPR}}_d$ the corresponding probability of success. We use the superscript $\mathrm {EPR}$ to keep the analogy with the case of deterministic PBT, as the measurement is optimized for the given resource state and no simplified measurement like the PGM is used. In [4], it was shown for qubits ($d=2$) that

$$\begin{aligned} p^{\mathrm {EPR}}_{2}(N)=1-\sqrt{\frac{8}{\pi N}} + o(1/\sqrt{N}). \end{aligned}$$

For arbitrary input dimension *d*, Studziński et al. [21] proved the exact formula

3.9 $$\begin{aligned} p^{\mathrm {EPR}}_d(N)=\frac{1}{d^N}\sum _{\alpha \vdash N-1}m_{d,\alpha }^2\frac{d_{\mu ^*}}{m_{d,\mu ^*}}, \end{aligned}$$

where $\mu ^*$ is the Young diagram obtained from $\alpha$ by adding one box in such a way that

3.10 $$\begin{aligned} \gamma _\mu (\alpha )=N\frac{m_{d,\mu } d_\alpha }{m_{d,\alpha } d_\mu } \end{aligned}$$

is maximized (as a function of $\mu$).

Finally, we note that any protocol for probabilistic PBT with success probability $p_d$ can be converted into a protocol for deterministic PBT by sending over a random port index to Bob whenever Alice’s measurement outcome indicates an error. The entanglement fidelity of the resulting protocol can be bounded as $F_d\ge p_d+\frac{1-p_d}{d^2}$. When applied to the fully optimized protocol corresponding to Eq. (3.8), this yields a protocol for deterministic PBT with better entanglement fidelity than the standard protocol for deterministic PBT. It uses, however, an optimized resource state that might be difficult to produce, while the standard protocol uses *N* maximally entangled states.

### Symmetries

The problem of port-based teleportation has several natural symmetries that can be exploited. Intuitively, we might expect a *U*(*d*)-symmetry and a permutation symmetry, since our figures of merit are unitarily invariant and insensitive to the choice of port that Bob has to select. For the resource state, we might expect an $S_N$-symmetry, while the POVM elements have a marked port, leaving a possible $S_{N-1}$-symmetry among the non-marked ports. This section is dedicated to making these intuitions precise.

The implications of the symmetries have been known for some time in the community and used in other works on port-based teleportation (e.g. in [22]). We provide a formal treatment here for the convenience of the interested reader as well as to highlight the fact that the unitary symmetry allows us to directly relate the entanglement fidelity (which a priori quantifies an average error) to the diamond norm error (a worst case figure of merit). This relation is proved in Corollary 3.5.

While the sub-structure of the resource state on Alice’s side in terms of *N* subsystems is natural from a mathematical point of view, it does not correspond to an operational feature of the task of PBT. This is in contrast to the port sub-structure on Bob’s side, in terms of which the port-based condition on the teleportation protocol is defined. In the following, it will be convenient to allow resource states for PBT to have an arbitrary sub-structure on Alice’s side.

We begin with a lemma on purifications of quantum states with a given group symmetry (see [47, 48] and [49, Lemma 5.5]):

#### Lemma 3.1

Let $\rho _A$ be a quantum state invariant under a unitary representation $\varphi$ of a group *G*, i.e., $[\rho _A,\varphi (g)]=0$ for all $g\in G$. Then there exists a purification $|\rho \rangle _{AA'}$ such that $(\varphi (g)\otimes \varphi ^*(g)) |\rho \rangle _{AA'}=|\rho \rangle _{AA'}$ for all $g\in G$. Here, $\varphi ^*$ is the dual representation of $\varphi$, which can be written as $\varphi ^*(g)=\overline{\varphi (g)}$.

Starting from an arbitrary port-based teleportation protocol, it is easy to construct a modified protocol that uses a resource state such that Bob’s marginal is invariant under the natural action of $S_N$ as well as the diagonal action of *U*(*d*). In slight abuse of notation, we denote by $\zeta _{B^N}$ the unitary representation of $\zeta \in S_N$ that permutes the tensor factors of $\mathcal {H}_B^{\otimes N}$.

#### Lemma 3.2

Let $\rho _{A^NB^N}$ be the resource state of a protocol for deterministic PBT with input dimension *d*. Then there exists another protocol for deterministic PBT with resource state $\rho '_{{A}^N{B}^NIJ}$, where *I* and *J* are additional registers held by Alice, such that $\rho '_{{B}^N}$ is invariant under the above-mentioned group actions,

3.11 $$\begin{aligned} \begin{aligned} U_B^{\otimes N}\rho '_{{B}^N}\left( U_B^{\otimes N}\right) ^\dagger&=\rho '_{{B}^N} \quad \text {for all }U_B\in U(d),\\ \zeta _{B^N} \rho '_{B^N} \zeta _{B^N}^\dagger&= \rho '_{B^N} \quad \text {for all }\zeta \in S_N, \end{aligned} \end{aligned}$$

and such that the new protocol has diamond norm error and entanglement fidelity no worse than the original one.

In fact, Lemma 3.2 applies not only to the diamond norm distance and the entanglement fidelity, but any convex functions on quantum channels that is invariant under conjugation with a unitary channel.

#### Proof of Lemma 3.2

Define the resource state

3.12 $$\begin{aligned} \tilde{\rho }_{A^NB^NI}= \frac{1}{N!} \sum _{\zeta \in S_N}\zeta _{B^N}\rho _{A^NB^N}\zeta _{B^N}^\dagger \otimes |\zeta \rangle \langle \zeta |_I, \end{aligned}$$

where $\zeta _{B^N}$ is the action of $S_N$ on $\mathcal {H}_B^{\otimes N}$ that permutes the tensor factors, and *I* is a classical ‘flag’ register with orthonormal basis $\lbrace |\zeta \rangle \rbrace _{\zeta \in S_N}$. The following protocol achieves the same performance as the preexisting one: Alice and Bob start sharing $\tilde{\rho }_{A^NB^NI}$ as an entangled resource, with Bob holding $B^N$ as usual and Alice holding registers $A^NI$. Alice begins by reading the classical register *I*. Suppose that its content is a permutation $\zeta$. She then continues to execute the original protocol, except that she applies $\zeta$ to the index she is supposed to send to Bob after her measurement, which obviously yields the same result as the original protocol.

A similar argument can be made for the case of *U*(*d*). Let $D\subset U(d)$, $|D|<\infty$ be an exact unitary *N*-design, i.e., a subset of the full unitary group such that taking the expectation value of any polynomial *P* of degree at most *N* in both *U* and $U^\dagger$ over the uniform distribution on *D* yields the same result as taking the expectation of *P* over the normalized Haar measure on *U*(*d*). Such exact *N*-designs exist for all *N* ([50]; see [51] for a bound on the size of exact *N*-designs). We now define a further modified resource state $\rho '_{A^NB^NIJ}$ from $\tilde{\rho }_{A^NB^NI}$ in analogy to (3.12):

$$\begin{aligned} \rho '_{A^NB^NIJ} = \frac{1}{|D|} \sum _{U\in D} U_B^{\otimes N} \tilde{\rho }_{A^NB^NI} (U_B^\dagger )^{\otimes N} \otimes |U\rangle \langle U|_J, \end{aligned}$$

where $\lbrace |U\rangle \rbrace _{U\in D}$ is an orthonormal basis for the flag register *J*. Again, there exists a modified protocol, in which Bob holds the registers $B^N$ as usual, but Alice holds registers $A^NIJ$. Alice starts by reading the register *J* which records the unitary $U\in D$ that has been applied to Bob’s side. She then proceeds with the rest of the protocol after applying $U^\dagger$ to her input state. Note that $\rho '_{B^N}$ clearly satisfies the symmetries in (3.11), and furthermore the new PBT protocol using $\rho '_{A^NB^NIJ}$ has the same performance as the original one using $\rho _{A^NB^N}$, concluding the proof. $\quad \square$

Denote by ${{\,\mathrm{Sym}\,}}^N(\mathcal {H})$ the symmetric subspace of a Hilbert space $\mathcal {H}^{\otimes N}$, defined by

$$\begin{aligned} {{{\,\mathrm{Sym}\,}}}^N(\mathcal {H})\,{:}{=}\, \lbrace |\psi \rangle \in \mathcal {H}^{\otimes N}:\pi |\psi \rangle = |\psi \rangle \text { for all }\pi \in S_N\rbrace . \end{aligned}$$

Using the above two lemmas we arrive at the following result.

#### Proposition 3.3

Let $\rho _{A^NB^N}$ be the resource state of a PBT protocol with input dimension *d*. Then there exists another protocol with properties as in Lemma 3.2 except that it has a resource state $|\psi \rangle \langle \psi |_{{A}^N{B}^N}$ with $|\psi \rangle _{{A}^N{B}^N}\in {{\,\mathrm{Sym}\,}}^{N}(\mathcal {H}_A\otimes \mathcal {H}_B)$ that is a purification of a symmetric Werner state, i.e., it is invariant under the action of *U*(*d*) on $\mathcal {H}_{A}^{\otimes N}\otimes \mathcal {H}_{B}^{\otimes N}$ given by $U^{\otimes N}\otimes \overline{U}^{\otimes N}$.

#### Proof

We begin by transforming the protocol according to Lemma 3.2, resulting in a protocol with resource state $\rho '_{A^NB^N I J}$ such that Bob’s part is invariant under the $S_N$ and *U*(*d*) actions. By Lemma 3.1, there exists a purification $|\psi \rangle _{{A}^N{B}^N}\in {{\,\mathrm{Sym}\,}}^n\left( \mathbb {C}^d\otimes \mathbb {C}^d\right)$ of $\rho '_{B^N}$ that is invariant under $U^{\otimes N}\otimes \overline{U}^{\otimes N}$ (note that the $S_n$-representation is self-dual, so the representation $\phi \otimes \phi ^*$ referred to in Lemma 3.1 just permutes the pairs of systems $A_iB_i$). But Uhlmann’s Theorem ensures that there exists an isometry $V_{A^N\rightarrow A^N I J E}$ for some Hilbert space $\mathcal {H}_E$ such that $V_{A^N\rightarrow A^N I J E}|\psi \rangle _{{A}^N{B}^N}$ is a purification of $\rho '_{A^NB^N I J}$. The following is a protocol using the resource state $|\psi \rangle$: Alice applies *V* and discards *E*. Then the transformed protocol from Lemma 3.2 is performed. $\quad \square$

Using the symmetries of the resource state, we can show that the POVM can be chosen to be symmetric as well. In the proposition below, we omit identity operators.

#### Proposition 3.4

Let $\{\left( E_i\right) _{A_0A^N}\}_{i=1}^N$ be Alice’s POVM for a PBT protocol with a resource state $|\psi \rangle$ with the symmetries from Proposition 3.3. Then there exists another POVM $\{\left( E'_i\right) _{A_0A^N}\}_{i=1}^N$ such that the following properties hold:

(i) $$ \zeta _{A^N}\left( E'_i\right) _{A_0A^N}\zeta _{A^N}^\dagger =\left( E'_{\zeta (i)}\right) _{A_0A^N}$$ for all $$\zeta \in S_N$$;
(ii) $\left( U_{A_0}\otimes \overline{U}_{A}^{\otimes N}\right) \left( E'_i\right) _{A_0A^N} \left( U_{A_0}\otimes \overline{U}_{A}^{\otimes N}\right) ^\dagger =\left( E'_i\right) _{A_0A^N}$ for all $U\in U(d)$;
(iii) the channel $\Lambda '$ implemented by the PBT protocol is unitarily covariant, i.e.,
  $$\begin{aligned} \Lambda '_{A_0\rightarrow B_0}(X)=U_{B_0} \Lambda '_{A_0\rightarrow B_0}(U_{A_0}^\dagger X U_{A_0})U_{B_0}^\dagger \quad \text {for all } U\in U(d); \end{aligned}$$
(iv) the resulting protocol has diamond norm distance (to the identity channel) and entanglement fidelity no worse than the original one.

#### Proof

Define an averaged POVM with elements

$$\begin{aligned} \left( E'_i\right) _{A_0A^N}=\int _{U(\mathcal {H}_A)} \mathrm {d}U \frac{1}{N!}\sum _{\zeta \in S_N} \left( U_{A_0}\otimes \overline{U}_{A}^{\otimes N}\zeta _{A^N}\right) \left( E_{\zeta ^{-1}(i)}\right) _{A_0A^N} \left( U_{A_0}^\dagger \otimes \zeta _{A^N}^\dagger (U_{A}^T)^{\otimes N}\right) , \end{aligned}$$

which clearly has the symmetries (i) and (ii). The corresponding channel can be written as

$$\begin{aligned} \Lambda '_{A_0\rightarrow B_0} = \int _{U(\mathcal {H}_A)} \frac{1}{N!} \sum _{\zeta \in S_N} \Lambda ^{(U,\zeta )}_{A_0\rightarrow B_0}, \end{aligned}$$

where

$$\begin{aligned}&\Lambda ^{(U,\zeta )}_{A_0\rightarrow B_0}(X_{A_0}) \\&\quad = \sum _{i=1}^N {{\,\mathrm{tr}\,}}_{A_0A^NB_i^c}\bigl [ \bigl ( (U_{A_0}\otimes \overline{U}_{A}^{\otimes N}\zeta _{A^N}) (E_{\zeta ^{-1}(i)})_{A_0A^N} (U_{A_0}^\dagger \otimes \zeta _{A^N}^\dagger (U_{A}^T)^{\otimes N}) \bigr )\\&\qquad \bigl (X_{A_0}\otimes |\psi \rangle \langle \psi |_{A^NB^N}\bigr ) \bigr ] \\&\quad = \sum _{i=1}^N {{\,\mathrm{tr}\,}}_{A_0A^NB_i^c}\bigl [ (E_{\zeta ^{-1}(i)})_{A_0A^N} \bigl (U_{A_0}^\dagger X_{A_0} U_{A_0} \\&\qquad \otimes (\zeta _{A^N}^\dagger (U_{A}^T)^{\otimes N} \otimes I_{B^N}) |\psi \rangle \langle \psi |_{A^NB^N} (\overline{U}_{A}^{\otimes N}\zeta _{A^N} \otimes I_{B^N}) \bigr ) \bigr ] \\&\quad = \sum _{i=1}^N {{\,\mathrm{tr}\,}}_{A_0A^NB_i^c}\bigl [ (E_{\zeta ^{-1}(i)})_{A_0A^N} \bigl (U_{A_0}^\dagger X_{A_0} U_{A_0}\\&\qquad \otimes (I_{A^N} \otimes U_B^{\otimes N} \zeta _{B^N}) |\psi \rangle \langle \psi |_{A^NB^N} (I_{A^N} \otimes \zeta _{B^N}^\dagger (U_B^\dagger )^{\otimes N}) \bigr ) \bigr ] \\&\quad = U_{B_0} \Lambda _{A_0\rightarrow B_0}(U_{A_0}^\dagger X_{A_0} U_{A_0}) U_{B_0}^\dagger , \end{aligned}$$

where we suppressed ${{\,\mathrm{id}\,}}_{B_i\rightarrow B_0}$. Here we used the cyclicity property

$$\begin{aligned} {{\,\mathrm{tr}\,}}_A[(X_A Y_A\otimes I_B) Z_{AB}] = {{\,\mathrm{tr}\,}}_A[(Y_A\otimes I_B) Z_{AB}(X_A\otimes I_B)] \end{aligned}$$

of the partial trace and the symmetries of the resource state, and $\Lambda _{A_0\rightarrow B_0}$ denotes the channel corresponding to the original protocol. It follows at once that $\Lambda '_{A_0\rightarrow B_0}$ is covariant in the sense of (iii). Finally, since the identity channel is itself covariant, property (iv) follows from the concavity (convexity) and unitary covariance of the entanglement fidelity and the diamond norm distance, respectively. $\quad \square$

Similarly as mentioned below Lemma 3.2, the statement in Proposition 3.4(iv) can be generalized to any convex function on the set of quantum channels that is invariant under conjugation with unitary channels.

The unitary covariance allows us to apply a lemma from [5] (stated as Lemma D.3 in “Appendix D”) to relate the optimal diamond norm error and entanglement fidelity of port-based teleportation. This shows that the achievability results Eqs. (3.5) to (3.4) for the entanglement fidelity of deterministic PBT, as well as the ones mentioned in the introduction, imply similar results for the diamond norm error without losing a dimension factor.

#### Corollary 3.5

Let $F_d^*$ and $\varepsilon _d^*$ be the optimal entanglement fidelity and optimal diamond norm error for deterministic PBT with input dimension *d*. Then, $\varepsilon _d^*=2\left( 1-F_d^*\right)$.

Note that the same formula was proven for the standard protocol in [5].

### Representation-theoretic characterization

The symmetries of PBT enable the use of representation-theoretic results, in particular Schur–Weyl duality. This was extensively done in [21, 22] in order to derive the formulas Eqs. (3.5)–(3.9). The main ingredient used in [21] to derive Eqs. (3.5) and (3.9) was the following technical lemma. For the reader’s convenience, we give an elementary proof in “Appendix A” using only Schur–Weyl duality and the classical Pieri rule. In the statement below, $B_i^c$ denotes the quantum system consisting of all *B*-systems except the *i*th one.

#### Lemma 3.6

[21]. The eigenvalues of the operator

$$\begin{aligned} T(N)_{AB^N} = \frac{1}{N} \left( \phi ^+_{AB_1}\otimes I_{B_1^c} + \ldots + \phi ^+_{AB_N}\otimes I_{B_N^c} \right) \end{aligned}$$

on $(\mathbb {C}^d)^{\otimes (1+N)}$ are given by the numbers

$$\begin{aligned} \frac{1}{dN} \gamma _\mu (\alpha ) = \frac{1}{d} \frac{d_\alpha m_{d,\mu }}{d_\mu m_{d,\alpha }}, \end{aligned}$$

where $\alpha \vdash _{d}N-1$, the Young diagram $\mu \vdash _d N$ is obtained from $\alpha$ by adding a single box, and $\gamma _\mu (\alpha )$ is defined in Eq. (3.10).

Note that the formula in Lemma 3.6 above gives *all* eigenvalues of $T(N)_{AB^N}$, i.e., including multiplicities.

The connection to deterministic PBT is made via the equivalence with state discrimination. In particular, when using a maximally entangled resource, *T*(*N*) is a rescaled version of the density operator corresponding to the ensemble of quantum states $\eta _i$ from Eq. (3.1),

$$\begin{aligned} T(N) =\frac{d^{N-1}}{N}\sum _i\eta _i. \end{aligned}$$

Using the hook length formulas Eqs. (2.1) and (2.2), we readily obtain the following simple expression for the ratio $\gamma _\mu (\alpha )$ defined in Eq. (3.10):

#### Lemma 3.7

[52] Let $$\mu =\alpha +e_i$$. Then,

$$\begin{aligned} \gamma _\mu (\alpha ) = \mu _i - i + d = \alpha _i - i + d + 1, \end{aligned}$$

i.e.,

$$\begin{aligned} \frac{d_\alpha m_{d,\mu }}{d_\mu m_{d,\alpha }} = \frac{\alpha _i - i + d + 1}{N}. \end{aligned}$$

#### Proof

Using Eqs. (2.1) and (2.2), we find

$$\begin{aligned}&\gamma _\mu (\alpha ) = N \frac{m_{d,\mu } d_\alpha }{m_{d,\alpha } d_\mu } = N \prod _{(i,j)\in \mu } \frac{d+c(i,j)}{h_\mu (i,j)} \frac{\prod _{(i,j)\in \mu }h_\mu (i,j)}{N!} \frac{(N-1)!}{\prod _{(i,j)\in \alpha }h_\alpha (i,j)}\\&\qquad \times \prod _{(i,j)\in \alpha } \frac{h_\alpha (i,j)}{d+c(i,j)} \\&\quad = \prod _{(i,j)\in \mu } \frac{d+c(i,j)}{1} \prod _{(i,j)\in \alpha } \frac{1}{d+c(i,j)} = d + c(i, \mu _i) = d + \mu _i - i, \end{aligned}$$

which concludes the proof. $\quad \square$

#### Remark 3.8

It is clear that $\gamma _\mu (\alpha )$ is maximized for $\alpha =(N-1,0,\ldots ,0)$ and $i=1$. Therefore,

$$\begin{aligned} \Vert T(N) \Vert _\infty = \frac{N+d-1}{dN}. \end{aligned}$$

This result can be readily used to characterize the extendibility of isotropic states, providing an alternative proof of the result by Johnson and Viola [53].

## The Schur–Weyl Distribution

Our results rely on the asymptotics of the Schur–Weyl distribution, a probability distribution defined below in (4.1) in terms of the representation-theoretic quantities that appear in the Schur–Weyl duality (2.4). These asymptotics can be related to the random matrix ensemble ${\text {GUE}}^0_d$. In this section we explain this connection and provide a refinement of a convergence result (stated in (4.4)) by Johansson [36] that is tailored to our applications. While representation-theoretic techniques have been extensively used in previous analyses, the connection between the Schur–Weyl distribution and random matrix theory has, to the best of our knowledge, not been previously recognized in the context of PBT (see however [31] for applications in the the context of quantum state tomography).

Recalling the Schur–Weyl duality ${\left( \mathbb {C}^d\right) }^{\otimes n} \cong \bigoplus _{\alpha \vdash _d n} [\alpha ] \otimes V^d_\alpha$, we denote by $P_\alpha$ the orthogonal projector onto the summand labeled by the Young diagram $\alpha \vdash _d n$. The collection of these projectors defines a projective measurement, and hence

4.1 $$\begin{aligned} p_{d,n}(\alpha ) \,{:}{=}\, {{\,\mathrm{tr}\,}}\left( P_\alpha \tau _d^{\otimes n}\right) = \frac{d_\alpha m_{d,\alpha }}{d^n} \end{aligned}$$

with $\tau _d=\frac{1}{d}1_{\mathbb {C}^d}$ defines a probability distribution on Young diagrams $\alpha \vdash _d n$, known as the *Schur–Weyl distribution*. Now suppose that $\varvec{\alpha }^{(n)} \sim p_{d,n}$ for $n\in \mathbb {N}$. By spectrum estimation [25–27, 54, 55], it is known that

4.2 $$\begin{aligned} \frac{\varvec{\alpha }^{(n)}}{n} \,\xrightarrow {P}\, (\tfrac{1}{d},\ldots ,\tfrac{1}{d}) \quad \text {as }n\rightarrow \infty . \end{aligned}$$

This can be understood as a *law of large numbers*. Johansson [36] proved a corresponding *central limit theorem*: Let $\mathbf {A}^{(n)}$ be the centered and renormalized random variable defined by

4.3 $$\begin{aligned} \mathbf {A}^{(n)} \,{:}{=}\, \frac{\varvec{\alpha }^{(n)} - (\tfrac{n}{d},\ldots ,\tfrac{n}{d})}{\sqrt{n/d}}. \end{aligned}$$

Then Johansson [36] proved that

4.4 $$\begin{aligned} \mathbf {A}^{(n)} \,\xrightarrow {D}\, {\text {spec}}(\mathbf {G}) \end{aligned}$$

for $n\rightarrow \infty$, where $\mathbf {G} \sim {\text {GUE}}^0_d$. The result for the first row is by Tracy and Widom [56] (cf. [36, 57]; see [31] for further discussion).

In the following sections, we would like to use this convergence of random variables stated in Eqs. (4.2) and (4.4) to determine the asymptotics of Eqs. (3.9) and (3.5). To this end, we rewrite the latter as expectation values of some functions of Young diagrams drawn according to the Schur–Weyl distribution. However, in order to conclude that these expectation values converge to the corresponding expectation values of functions on the spectrum of ${\text {GUE}}^0_d$-matrices, we need a stronger sense of convergence than what is provided by the former results. Indeed, we need to establish convergence for functions that diverge polynomially as $n\rightarrow \infty$ when $A_{j}=\omega (1)$ or when $A_{j}=O(n^{-1/2})$.^4^ The former are easily handled using the bounds from spectrum estimation [27], but for the latter a refined bound on $p_{d,n}$ corresponding to small *A* is needed. To this end, we prove the following result, which shows convergence of expectation values of a large class of functions that includes all polynomials in the variables $\mathbf {A}_i$.

In the following, we will need the cone of sum-free non-increasing vectors in $\mathbb {R}^d$,

$$\begin{aligned} C^d=\left\{ x\in \mathbb {R}^d:\sum \nolimits _i x_i=0, \ x_i\ge x_{i+1}\right\} , \end{aligned}$$

and its interior $\mathrm {int}(C^d)=\lbrace x\in C^d:x_i \ne 0 \text { for }i=1,\ldots ,d\rbrace$.

### Theorem 4.1

Let $g:\mathrm {int}(C^d)\rightarrow \mathbb {R}$ be a continuous function satisfying the following: There exist constants $\eta _{ij}$ satisfying $\eta _{ij}> -2-\frac{1}{d-1}$ such that for

$$\begin{aligned} \varphi _{\eta }(x) \,{:}{=}\, \prod _{i<j}\left( x_i-x_j\right) ^{\eta _{ij}} \end{aligned}$$

there exists a polynomial *q* with

$$\begin{aligned} \frac{g(x)}{\varphi _{\eta }(x)}\le q(\Vert x\Vert _1). \end{aligned}$$

For every *n*, let $\mathbf \alpha ^{(n)} \sim p_{d,n}$ be drawn from the Schur–Weyl distribution, $\mathbf{A}^{(n)} \,{:}{=}\, \sqrt{d/n}(\mathbf \alpha ^{(n)}-n/d)$ the corresponding centered and renormalized random variable, and $\tilde{\mathbf{A}}^{(n)}=\mathbf{A}^{(n)}+\frac{d-i}{\sqrt{\frac{n}{d}}}$. Then the family of random variables $\left\{ g\left( \tilde{\mathbf{A}}^{(n)}\right) \right\} _{n\in \mathbb {N}}$ is uniformly integrable and

$$\begin{aligned} \lim _{n\rightarrow \infty } \mathbb {E}\left[ g\left( \tilde{\mathbf{A}}^{(n)}\right) \right] = \mathbb {E}\left[ g(\mathbf{A}) \right] , \end{aligned}$$

where $$\mathbf{A}=\mathrm {spec}(\mathbf {G})$$ and $$\mathbf {G} \sim {\text {GUE}}^0_d$$.

As a special case we recover the uniform integrability of the moments of $\mathbf {A}$ (Corollary 4.5), which implies convergence in distribution in the case of an absolutely continuous limiting distribution. Therefore, Theorem 4.1 is a refinement of the result (4.4) by Johansson. The remainder of this section is dedicated to proving Theorem 4.1.

The starting point for what follows is Stirling’s approximation, which states that

$$\begin{aligned} \sqrt{2\pi } \sqrt{n} \left( \frac{n}{e} \right) ^n \le n!\le e \sqrt{n} \left( \frac{n}{e} \right) ^n \quad \text {for all }n\in \mathbb {N}. \end{aligned}$$

It will be convenient to instead use the following variant,

4.5 $$\begin{aligned} \frac{\sqrt{2\pi }}{e} \sqrt{n+1} \left( \frac{n}{e} \right) ^n \le n!&\le e \sqrt{n} \left( \frac{n}{e} \right) ^n, \end{aligned}$$

where the upper bound is unchanged and the lower bound follows using $n!=\frac{(n+1)!}{n+1}$. The dimension $d_\alpha$ is equal to the multinomial coefficient up to inverse polynomial factors [27]. Defining the normalized Young diagram $\bar{\alpha }=\frac{\alpha }{n}$ for $\alpha \vdash n$, the multinomial coefficient $$\left( {\begin{array}{c}n\\ \alpha \end{array}}\right) $$ can be bounded from above using Eq. (4.5) as

$$\begin{aligned} \left( {\begin{array}{c}n\\ \alpha \end{array}}\right) = \frac{n!}{\alpha _1!\ldots \alpha _d!} \le C_d \sqrt{\frac{n}{\prod _{i=1}^d (\alpha _i+1)}} \, \frac{n^n}{\alpha _1^{\alpha _1}\ldots \alpha _d^{\alpha _d}}, \end{aligned}$$

where $$C_d \,{:}{=}\, \frac{e^{d+1}}{(2\pi )^{d/2}}$$. Hence,

4.6 $$\begin{aligned} d^{-n} \left( {\begin{array}{c}n\\ \alpha \end{array}}\right)&\le C_d\sqrt{\frac{n}{\prod _{i=1}^d (\alpha _i+1)}} \exp \left( -n D(\bar{\alpha }\Vert \tau )\right) \nonumber \\&\le C_d\sqrt{\frac{n}{\prod _{i=1}^d (\alpha _i+1)}} \exp \left( -\frac{n}{2} \left\Vert \bar{\alpha }- \tau \right\Vert _1^2\right) \nonumber \\&= C_dn^{-\frac{d-1}{2}} \left[ \prod _{i=1}^d \left( \bar{\alpha }_i+\frac{1}{n} \right) ^{-\frac{1}{2}} \right] \exp \left( -\frac{n}{2} \left\Vert \bar{\alpha }- \tau \right\Vert _1^2\right) . \end{aligned}$$

Here, $D(p\Vert q)\,{:}{=}\,\sum _i p_i \log {p_i}/\!{q_i}$ is the Kullback-Leibler divergence defined in terms of the natural logarithm, $\tau =(1/d,\ldots ,1/d)$ is the uniform distribution, and we used Pinsker’s inequality [58] in the second step.

We go on to derive an upper bound on the probability of Young diagrams that are close to the boundary of the set of Young diagrams under the Schur–Weyl distribution. More precisely, the following lemma can be used to bound the probability of Young diagrams that have two rows that differ by less than the generic $O(\sqrt{n})$ in length.

### Lemma 4.2

Let $d\in \mathbb {N}$ and $c_1,\ldots ,c_{d-1}\ge 0$, $\gamma _1,\ldots ,\gamma _{d-1}\ge 0$. Let $\alpha \vdash _dn$ be a Young diagram with (a) $\alpha _i-\alpha _{i+1}\le c_i n^{\gamma _i}$ for all *i*. Finally, set $A \,{:}{=}\, \sqrt{d/n}(\alpha -n/d)$. Then,

$$\begin{aligned} p_{d,n}(\alpha ) \le C n^{-\frac{d^2-1}{2}+2\sum _{i<j}\gamma _{ij}} \left[ \prod _{i=1}^{d}\left( 1+\sqrt{\frac{d}{n}}A_i+\frac{d}{n}\right) ^{i-d-\frac{1}{2}} \right] \exp \left( -\frac{1}{2d} \left\| A\right\| _1^2\right) , \end{aligned}$$

where $\gamma _{ij}\,{:}{=}\,\max \{\gamma _i,\gamma _{i+1},\ldots ,\gamma _{j-1}\}$ and $C=C(c_1,\ldots ,c_{d-1},d)$ is a suitable constant.

### Proof

We need to bound $p_{d,n}(\alpha )=m_{d,\alpha } d_\alpha / d^n$ and begin with $m_{d,\alpha }$. By assumption (a), there exist constants $C_{ij}>0$ (depending on $c_i,\ldots ,c_{j-1}$ as well as on *d*) such that the inequality $\alpha _i-\alpha _j+j-i\le C_{ij}n^{\gamma _{ij}}$ holds for all $i<j$. Using the Weyl dimension formula (2.3) and assumption (a), it follows that

4.7 $$\begin{aligned} m_{d,\alpha }&= \prod _{i<j}\frac{\alpha _i-\alpha _j+j-i}{j-i} \le C_1 \, n^{\sum _{i<j}\gamma _{ij}} \end{aligned}$$

for a suitable constant $C_1=C_1(c_1,\ldots ,c_{d-1},d)>0$. Next, consider $d_\alpha$. By comparing the hook-length formulas (2.1) and (2.2), we have

4.8 $$\begin{aligned} d_\alpha&= n! \, m_{d,\alpha }\left[ \prod _{(i,j)\in \alpha }(d+j-i) \right] ^{-1}\nonumber \\&= n! \, m_{d,\alpha }\left[ \prod _{i=1}^d\frac{(\alpha _i+d-i)!}{(d-i)!} \right] ^{-1}\nonumber \\&\le n! \, m_{d,\alpha }\left[ \prod _{i=1}^d\frac{(\alpha _i+1)^{d-i} \alpha _i!}{(d-i)!} \right] ^{-1}\nonumber \\&= m_{d,\alpha } \left[ \prod _{i=1}^d\frac{(d-i)!}{(\alpha _i+1)^{d-i}} \right] \left( {\begin{array}{c}n\\ \alpha \end{array}}\right) \nonumber \\&= C_2 \, m_{d,\alpha } n^{-\frac{d(d-1)}{2}} \left[ \prod _{i=1}^d \left( \bar{\alpha }_i + \frac{1}{n} \right) ^{i-d} \right] \left( {\begin{array}{c}n\\ \alpha \end{array}}\right) , \end{aligned}$$

where $C_2=C_2(d)>0$, and $\bar{\alpha }_i = \alpha _i/n$. In the inequality, we used that $\alpha _i + d - i \ge \alpha _i + 1$ for $1\le i\le d-1$, and for $i=d$, the exponent of $\alpha _i + 1$ on the right hand side is zero.

Combining Eqs. (4.7)–(4.6) and setting $C_3=C_1^2 C_2C_d$, we obtain

$$\begin{aligned} p_{d,n}(\alpha ) = \frac{m_{d,\alpha } d_\alpha }{d^n}&\le C_2 m_{d,\alpha }^2 n^{-\frac{d(d-1)}{2}} \left[ \prod _{i=1}^d \left( \bar{\alpha }_i+\frac{1}{n}\right) ^{i-d} \right] d^{-n} \left( {\begin{array}{c}n\\ \alpha \end{array}}\right) \\&\le C_3 \, n^{-\frac{d^2-1}{2}+2\sum _{i<j}\gamma _{ij}} \left[ \prod _{i=1}^d \left( \bar{\alpha }_i+\frac{1}{n}\right) ^{i-d-\frac{1}{2}} \right] \exp \left( -\frac{n}{2} \left\Vert \bar{\alpha }- \tau \right\Vert _1^2\right) . \end{aligned}$$

Substituting $\bar{\alpha }_i = \frac{1}{d} + \frac{A_i}{\sqrt{nd}}$ we obtain the desired bound. $\quad \square$

In order to derive the asymptotics of entanglement fidelities for port-based teleportation, we need to compute limits of certain expectation values. As a first step, the following lemma ensures that the corresponding sequences of random variables are uniformly integrable. We recall that a family of random variables $\{\mathbf{X}^{(n)}\}_{n\in \mathbb {N}}$ is called *uniformly integrable* if, for every $\varepsilon >0$, there exists $K<\infty$ such that $\sup _n \mathbb {E}\left[ |\mathbf{X}^{(n)}|\cdot \mathbb {1}_{|\mathbf{X}^{(n)}|\ge K}\right] \le \varepsilon$.

### Lemma 4.3

Under the same conditions as for Theorem 4.1, the family of random variables $\left\{ g\left( \tilde{\mathbf{A}}^{(n)}\right) \right\} _{n\in \mathbb {N}}$ is uniformly integrable.

### Proof

Let $\mathbf{X}^{(n)} \,{:}{=}\, g\left( \tilde{\mathbf{A}}^{(n)}\right)$. The claimed uniform integrability follows if we can show that

4.9 $$\begin{aligned} \sup _n \mathbb {E}\left[ |\mathbf{X}^{(n)}|\right] <\infty \end{aligned}$$

for every choice of the $\eta _{ij}$. Indeed, to show that $\{ \mathbf{X}^{(n)} \}$ is uniformly integrable it suffices to show that $\sup _n \mathbb {E}\left[ |\mathbf{X}^{(n)} |^{1+\delta }\right] <\infty$ for some $\delta >0$ [59, Ex. 5.5.1]. If we choose $\delta >0$ such that $\eta '_{ij} \,{:}{=}\, (1+\delta )\eta _{ij} > -2-\frac{1}{d-1}$ for all $1\le i < j \le d$, then it is clear that Eq. (4.9) for $\eta '_{ij}$ implies uniform integrability of the original family.

Moreover, we may also assume that $h_{\eta }\equiv g/\varphi _{\eta }=1$, since the general case then follows from the fact that $p_{d,n}(\alpha )$ decays exponentially in $\Vert A\Vert _1$ (see Lemma 4.2). More precisely, for any polynomial *r* and any constant $\theta _1>0$ there exist constants $\theta _2, \theta _3>0$ such that

$$\begin{aligned} r(\Vert x\Vert _1)\exp \left( -\theta _1\Vert x\Vert _1\right) \le \theta _2\exp \left( -\theta _3\Vert x\Vert _1\right) . \end{aligned}$$

In particular, this holds for the polynomial *q* bounding *h* from above by assumption. When proving the statement $\sup _n \mathbb {E}\left[ |\mathbf{X}^{(n)} |\right] <\infty$, the argument above allows us to reduce the general case $h_{\eta } = g/\varphi _{\eta }\ne 1$ to the case $h_{\eta }=1$, or equivalently, to

$$\begin{aligned} g(x) = \varphi _{\eta }(x) = \prod _{i<j}\left( x_i-x_j\right) ^{\eta _{ij}}. \end{aligned}$$

Thus, it remains to be shown that

4.10 $$\begin{aligned} \sup _n \mathbb {E}\left[ f^{(n)}(\mathbf{A}^{(n)})\right] <\infty , \end{aligned}$$

where

$$\begin{aligned} f^{(n)}(A) \,{:}{=}\, \varphi _{\eta }(\tilde{A})=\prod _{i<j}\left( A_i-A_j+\frac{j-i}{\sqrt{n/d}}\right) ^{\eta _{ij}} \end{aligned}$$

for some constants $\eta _{ij}$ satisfying the assumption of Theorem 4.1 that we fix for the rest of this proof. Define $\Gamma _{ij}\,{:}{=}\, A_i-A_j+\frac{j-i}{\sqrt{n/d}}$. Then we have $f^{(n)}(A) = \prod _{i<j} \Gamma _{ij}^{\eta _{ij}}$, while the Weyl dimension formula (2.3) becomes

$$\begin{aligned} m_{d,\alpha } = \left( \frac{n}{d} \right) ^{\frac{d(d-1)}{4}} \prod _{i<j} \frac{\Gamma _{ij}}{j-i}. \end{aligned}$$

Hence, together with Eqs. (4.8) and (4.6) we can bound $p_{d,n}(\alpha ) = m_{d,\alpha } d_{\alpha } / d^n$ as

4.11 $$\begin{aligned} p_{d,n}(\alpha )&\le C n^{-\frac{d-1}{2}} \Bigg ( \prod _{i<j} \Gamma _{ij}^{2} \Bigg ) \left[ \prod _{i=1}^d \left( \bar{\alpha }_i + \frac{1}{n}\right) ^{i-d-\frac{1}{2}} \right] \exp \left( \frac{n}{2}\Vert \bar{\alpha }-\tau \Vert _1^2\right) \nonumber \\&= C n^{-\frac{d-1}{2}} \Bigg ( \prod _{i<j} \Gamma _{ij}^{2} \Bigg ) \left[ \prod _{i=1}^d \left( 1 + \sqrt{\frac{d}{n}}A_i + \frac{d}{n}\right) ^{i-d-\frac{1}{2}} \right] \exp \left( -\frac{1}{2d} \left\Vert A\right\Vert _1^2\right) , \end{aligned}$$

where $C=C(d)$ is some constant, and we used $\bar{\alpha }_i + \frac{1}{n} = \frac{1}{d}\Big (\sqrt{\frac{d}{n}} A_i + \frac{d}{n} + 1 \Big )$ and $\tau =(1/d,\ldots ,1/d)$ in the equality. Using $f^{(n)}(A) = \prod _{i<j} \Gamma _{ij}^{\eta _{ij}}$, this yields the bound

4.12 $$\begin{aligned} p_{d,n}(\alpha ) \, f^{(n)}(A)&\le C n^{-\frac{d-1}{2}} \left( \prod _{i<j} \Gamma _{ij}^{2+\eta _{ij}} \right) \left[ \prod _{i=1}^d \left( 1 + \sqrt{\frac{d}{n}}A_i + \frac{d}{n} \right) ^{i-d-\frac{1}{2}} \right] \nonumber \\&\quad \exp \left( -\frac{1}{2d} \left\Vert A\right\Vert _1^2\right) . \end{aligned}$$

We now want to bound the expectation value in Eq. (4.10) and begin by splitting the sum over Young diagrams according to whether $\exists i: |A_i|>n^\varepsilon$ for some $\varepsilon \in (0,\frac{1}{2})$ to be determined later, or $|A_i|\le n^\varepsilon$ for all *i*. We denote the former event by $\mathcal {E}$ and obtain

4.13 $$\begin{aligned} \mathbb {E}\left[ f^{(n)}(\mathbf{A}^{(n)})\right] = \mathbb {E}\left[ f^{(n)}(\mathbf{A}^{(n)}) \mathbb {1}_\mathcal {E}\right] + \mathbb {E}\left[ f^{(n)}(\mathbf{A}^{(n)}) \mathbb {1}_{\mathcal {E}^c}\right] . \end{aligned}$$

We treat the two expectation values in (4.13) separately and begin with the first one. If $|A_i|>n^\varepsilon$ for some *i*, then $\Vert A\Vert _1^2 \ge n^{2\varepsilon }$, so it follows by Eq. (4.12) that

$$\begin{aligned}&\mathbb {E}\left[ f^{(n)}(\mathbf{A}^{(n)}) \mathbb {1}_\mathcal {E}\right] \\&\quad = \sum _{\begin{array}{c} \alpha \vdash _d n \text { s.t. } \\ \exists i: |A_i|> n^\varepsilon \end{array}} p_{d,n}(\alpha ) f^{(n)}(A) \\&\qquad \le C \sum _{\begin{array}{c} \alpha \vdash _d n \text { s.t. } \\ \exists i: |A_i|> n^\varepsilon \end{array}} n^{-\frac{d-1}{2}} \left( \prod _{i<j} \Gamma _{ij}^{2+\eta _{ij}} \right) \left[ \prod _{i=1}^d \left( 1 + \sqrt{\frac{d}{n}}A_i + \frac{d}{n} \right) ^{i-d-\frac{1}{2}} \right] \\&\qquad \exp \left( -\frac{1}{2d} \left\Vert A\right\Vert _1^2\right) \\&\quad \le {{\,\mathrm{poly}\,}}(n) \exp \left( -\frac{1}{2d} n^{2\varepsilon }\right) . \end{aligned}$$

Here, ${{\,\mathrm{poly}\,}}(n)$ denotes some polynomial in *n* and we also used that, for fixed *d*, the number of Young diagrams is polynomial in *n*. This shows that the first expectation value in (4.13) vanishes for $n\rightarrow \infty$.

For the second expectation value, note that $|A_i|\le n^{\varepsilon }=o(\sqrt{n})$ for all *i*, and hence there exists a constant $K>0$ such that we have

4.14 $$\begin{aligned} \prod _{i=1}^d \left( 1 + \sqrt{\frac{d}{n}}A_i + \frac{d}{n} \right) ^{i-d-\frac{1}{2}} \le K. \end{aligned}$$

Using Eqs. (4.12) and (4.14), we can therefore bound

$$\begin{aligned} \mathbb {E}\left[ f^{(n)}(\mathbf{A}^{(n)}) \mathbb {1}_{\mathcal {E}^c}\right]&= \sum _{\begin{array}{c} \alpha \vdash _d n \text { s.t. } \\ \forall i: |A_i|\le n^\varepsilon \end{array}} p_{d,n}(\alpha ) f^{(n)}(A) \\&\le C K \sum _{A\in \mathcal {D}_n} n^{-\frac{d-1}{2}} \left( \prod _{i<j} \Gamma _{ij}^{2+\eta _{ij}} \right) \exp \left( -\frac{1}{2d} \left\Vert A\right\Vert _1^2\right) , \end{aligned}$$

where we have introduced $\mathcal {D}_n \,{:}{=}\, \{ A:\alpha \vdash _d n \}$. The summands are nonnegative, even when evaluated on any point in the larger set $\hat{\mathcal {D}}_n \,{:}{=}\, \left\{ A \in \sqrt{\frac{d}{n}}\left( \mathbb {Z}-\frac{n}{d}\right) ^d : \sum _i A_i = 0, A_i \ge A_{i+1} \forall i \right\} \supset \mathcal {D}_n$, so that we have the upper bound

4.15 $$\begin{aligned} \mathbb {E}\left[ f^{(n)}(\mathbf{A}^{(n)}) \mathbb {1}_{\mathcal {E}^c}\right]&\le C K \sum _{A\in \mathcal {D}_n} n^{-\frac{d-1}{2}} \left( \prod _{i<j} \Gamma _{ij}^{2+\eta _{ij}} \right) \exp \left( -\frac{1}{2d} \left\Vert A\right\Vert _1^2\right) \nonumber \\&\le C K \sum _{A\in \hat{\mathcal {D}}_n} n^{-\frac{d-1}{2}} \left( \prod _{i<j} \Gamma _{ij}^{2+\eta _{ij}} \right) \exp \left( -\frac{1}{2d} \left\Vert A\right\Vert _1^2\right) . \end{aligned}$$

Let $x_i=A_i-A_{i+1}, \ i=1,\ldots ,d-1$. Next, we will upper bound the exponential in Eq. (4.15). For this, define $\tilde{x}_i=\max (\frac{1}{d-1},x_i)$ and let $S=\{ i\in \{1,\ldots ,d-1\} \;|\; x_i\le \frac{1}{d-1} \}$. Then, assuming $S^c\ne \emptyset$,

$$\begin{aligned} \sum _{i=1}^{d-1}\tilde{x}_i&\le \left( \sum _{i=1}^{d-1}\tilde{x}_i\right) ^2 = \left( \sum _{i\in S}\tilde{x}_i+\sum _{i\in S^c}\tilde{x}_i\right) ^2 = \left( \frac{|S|}{d-1} + \sum _{i\in S^c} x_i\right) ^2\\&=\left( \frac{|S|}{d-1} \right) ^2 + 2 \frac{|S|}{d-1} \left( \sum _{i\in S^c} x_i \right) + \left( \sum _{i\in S^c} x_i \right) ^2\\&\le \left( \frac{|S|}{d-1} \right) ^2 + 2 \frac{|S|}{d-1} \frac{d-1}{|S^c|} \left( \sum _{i\in S^c} x_i \right) ^2 + \left( \sum _{i\in S^c} x_i \right) ^2\\&= \left( \frac{|S|}{d-1} \right) ^2 + \left( 1 + 2 \frac{|S|}{|S^c|} \right) \left( \sum _{i\in S^c} x_i \right) ^2\\&\le 1 + \left( 2d - 1 \right) \left( \sum _{i=1}^{d-1} x_i \right) ^2 \end{aligned}$$

since $\sum _{i\in S^c} x_i \ge \frac{|S^c|}{d-1}$. This bounds also holds when $S^c=\emptyset$. Hence,

4.16 $$\begin{aligned} \exp \left( -\frac{1}{2d} \left\Vert A\right\Vert _1^2\right) \le \exp \left( -\frac{1}{2d}\left( \sum _{i=1}^{d-1}x_i\right) ^2\right) \le R\exp \left( -\gamma \sum _{i=1}^{d-1}\tilde{x}_i \right) =R\prod _{i=1}^{d-1}\exp \left( -\gamma \tilde{x}_i\right) , \end{aligned}$$

where $\gamma \,{:}{=}\, \frac{1}{2d(2d-1)}$ and $R \,{:}{=}\, e^{\gamma }$. The first inequality follows from $\sum _{i=1}^{d-1}x_i=A_1-A_d=|A_1|+|A_d| \le \Vert A\Vert _1$. If we use Eq. (4.16) in Eq. (4.15) we obtain the upper bound

4.17 $$\begin{aligned} \mathbb {E}\left[ f^{(n)}(\mathbf{A}^{(n)}) \mathbb {1}_{\mathcal {E}^c}\right] \le C' \sum _{A\in \hat{\mathcal {D}}_n} n^{-\frac{d-1}{2}} \left( \prod _{i<j} \Gamma _{ij}^{2+\eta _{ij}} \right) \prod _{i=1}^{d-1}\exp \left( -\gamma \tilde{x}_i\right) \end{aligned}$$

where $$C' \,{:}{=}\, CKR$$.

Let us first assume that all $\eta _{ij} \le -2$, so that $2+\eta _{ij}\in (-\frac{1}{d-1},0]$. Since

$$\begin{aligned} \Gamma _{ij} = \left( \sum _{l=i}^{j-1}x_l\right) +\frac{j-i}{\sqrt{\frac{n}{d}}} = \sum _{l=i}^{j-1}\left( x_l+\frac{1}{\sqrt{\frac{n}{d}}}\right) \ge x_{i}+\frac{1}{\sqrt{\frac{n}{d}}} \end{aligned}$$

and $\eta _{ij}+2\le 0$, we have that

4.18 $$\begin{aligned} \Gamma _{ij}^{2+\eta _{ij}}\le \left( x_{i}+\frac{1}{\sqrt{\frac{n}{d}}}\right) ^{2+\eta _{ij}}, \end{aligned}$$

as power functions with non-positive exponent are non-increasing. We can then upper-bound Eq. (4.17) as follows,

$$\begin{aligned} \mathbb {E}\left[ f^{(n)}(\mathbf{A}^{(n)}) \mathbb {1}_{\mathcal {E}^c}\right]&\le C' \sum _{A\in \hat{\mathcal {D}}_n} n^{-\frac{d-1}{2}} \left( \prod _{i<j} \Gamma _{ij}^{2+\eta _{ij}} \right) \prod _{i=1}^{d-1}\exp \left( -\gamma \tilde{x}_i\right) \\&\le C'\sum _{A\in \hat{\mathcal {D}}_n} n^{-\frac{d-1}{2}} \left( \prod _{i<j} \left( x_{i}+\frac{1}{\sqrt{\frac{n}{d}}}\right) ^{2+\eta _{ij}} \right) \prod _{i=1}^{d-1}\exp \left( -\gamma \tilde{x}_i\right) , \\&= C'\sum _{A\in \hat{\mathcal {D}}_n} n^{-\frac{d-1}{2}} \left( \prod _{i=1}^{d-1} \left( x_{i}+\frac{1}{\sqrt{\frac{n}{d}}}\right) ^{\sum _{j=i+1}^d(2+\eta _{ij})} \right) \prod _{i=1}^{d-1}\exp \left( -\gamma \tilde{x}_i\right) , \\&= C'\prod _{i=1}^{d-1}\left( n^{-\frac{1}{2}} \sum _{x_i\in \sqrt{\frac{d}{n}}\mathbb {N}} \left( x_{i}+\frac{1}{\sqrt{\frac{n}{d}}}\right) ^{\sum _{j=i+1}^d(2+\eta _{ij})} \exp \left( -\gamma \tilde{x}_i\right) \right) \end{aligned}$$

where the first inequality is Eq. (4.17) and in the second inequality we used Eq. (4.18). Since $\eta _{ij} > -2-\frac{1}{d-1}$ by assumption, it follows that $\sum _{j=i+1}^d (2+\eta _{ij}) > -\frac{d-i}{d-1} \ge -1$. Thus, each term in the product is a Riemann sum for an improper Riemann integral, as in Lemma D.4, which then shows that the expression converges for $n\rightarrow \infty$.

The case where some $\eta _{ij}>-2$ is treated by observing that

$$\begin{aligned} \prod _{\begin{array}{c} i<j:\\ \eta _{ij}>-2 \end{array}}\Gamma _{ij}^{2+\eta _{ij}}\exp \left( -\frac{1}{2d} \left\Vert A\right\Vert _1^2\right) \le c_1\exp \left( -\frac{c_2}{2d} \left\Vert A\right\Vert _1^2\right) \end{aligned}$$

for suitable constants $c_1,c_2>0$. We can use this bound in Eq. (4.17) to replace each $\eta _{ij}>-2$ by $\eta _{ij}=-2$, at the expense of modifying the constants $C'$ and $\gamma$, and then proceed as we did before. This concludes the proof of Eq. (4.10). $\quad \square$

The uniform integrability result of Lemma 4.3 implies that the corresponding expectation values converge. To determine their limit in terms of the expectation value of a function of the spectrum of a ${\text {GUE}}^0_d$-matrix, however, we need to show that we can take the limit of the dependencies on *n* of the function and the random variable $\mathbf {A}^{(n)}$ separately. This is proved in the following lemma, where we denote the interior of a set *E* by int(*E*).

### Lemma 4.4

Let $\lbrace \mathbf {A}^{(n)}\rbrace _{n\in \mathbb {N}}$ and $\mathbf {A}$ be random variables on a Borel measure space *E* such that $\mathbf {A}^{(n)}\overset{D}{\rightarrow }\mathbf {A}$ for $n\rightarrow \infty$ and $\mathbf {A}$ is absolutely continuous. Let $f:\mathrm {int}(E)\rightarrow \mathbb {R}$. Let further $f_n: E\rightarrow \mathbb {R}$, $n\in \mathbb {N}$, be a sequence of continuous bounded functions such that $f_n\rightarrow f$ pointwise on $\mathrm {int}(E)$ and, for any compact $S\subset \mathrm {int}(E)$, $\{f_n|_S\}_{n\in \mathbb {N}}$ is uniformly equicontinuous and $f_n|_S\rightarrow f|_S$ uniformly. Then for any such compact $S\subset \mathrm {int}(E)$, the expectation value $\mathbb {E}\left[ f(\mathbf {A})\mathbb {1}_S(\mathbf {A})\right]$ exists and

$$\begin{aligned} \lim _{n\rightarrow \infty }\mathbb {E}\left[ f_n(\mathbf {A}^{(n)})\mathbb {1}_S (\mathbf {A}^{(n)})\right] =\mathbb {E}\left[ f(\mathbf {A})\mathbb {1}_S(\mathbf {A})\right] . \end{aligned}$$

### Proof

For $$n,m\in \mathbb {N}\cup \{\infty \}$$, define

$$\begin{aligned} b_{nm}(S)=\mathbb {E}\left[ f_n(\mathbf {A}^{(m)})\mathbb {1}_S(\mathbf {A}^{(m)})\right] \end{aligned}$$

with $$f_\infty \,{:}{=}\, f$$, $$\mathbf {A}^{(\infty )}\,{:}{=}\, \mathbf {A}$$ and $$S\subset \mathrm {int}(E)$$ compact. These expectation values readily exist as $$f_n$$ is bounded for all *n*, and the uniform convergence of $$f_n|_S $$ implies that $$f|_S$$ is continuous and bounded as well. The uniform convergence $$f_n|_S\rightarrow f|_S$$ implies that $$f_n|_S$$ is uniformly bounded, so by Lebesgue’s theorem of dominated convergence $$b_{\infty m}(S)$$ exists for all $$m\in \mathbb {N}$$ and

4.19 $$\begin{aligned} \lim _{n\rightarrow \infty }b_{nm}(S)=b_{\infty m}(S)\ \forall m\in \mathbb {N}\cup \{\infty \}. \end{aligned}$$

This convergence is even uniform in *m* which follows directly from the uniform convergence of $f_n|_S$. The sequence $\lbrace \mathbf {A}^{(n)}\rbrace _{n\in \mathbb {N}}$ of random variables converges in distribution to the absolutely continuous $\mathbf {A}$, so the expectation value of any continuous bounded function converges. Therefore,

4.20 $$\begin{aligned} \lim _{m\rightarrow \infty }b_{nm}(S)=b_{n\infty }(S)\ \forall n\in \mathbb {N}\cup \{\infty \}. \end{aligned}$$

An inspection of the proof of Theorem 1, Chapter VIII in [60] reveals the following: The fact that the uniform continuity and boundedness of $f_n|_S$ hold uniformly in *n* implies the uniformity of the above limit. Moreover, since both limits exist and are uniform, this implies that they are equal to each other, and any limit of the form

$$\begin{aligned} \lim _{n\rightarrow \infty } b_{n m(n)} \end{aligned}$$

for $m(n)\xrightarrow {n\rightarrow \infty }\infty$ exists and is equal to the limits in Eqs. (4.19) and (4.20). $\quad \square$

Finally, we obtain the desired convergence theorem. For our applications, $\eta _{ij}\equiv -2$ suffices. The range of $\eta _{ij}$’s for which the lemma is proven is naturally given by the proof technique.

### Proof of Theorem 4.1

The uniform integrability of $\mathbf {X}^{(n)}\,{:}{=}\, g\left( \tilde{\mathbf {A}}^{(n)}\right)$ is the content of Lemma 4.3. Recall that uniform integrability means that

$$\begin{aligned} \lim _{K\rightarrow \infty } \sup _{n\in \mathbb {N}} \mathbb {E}\left[ \big |\mathbf {X}^{(n)}\big | \cdot \mathbb {1}_{\mathcal {E}_K^c}\left( \mathbf {A}^{(n)}\right) \right] = 0, \end{aligned}$$

where $\mathcal {E}_K\,{:}{=}\, \lbrace x\in \mathbb {R}^d:\Vert x\Vert _\infty \le K\rbrace$. Let now $\varepsilon >0$ be arbitrary, and $K<\infty$ be such that the following conditions are true:

$$\begin{aligned} \sup _{n\in \mathbb {N}}\mathbb {E}\left[ \big |\mathbf {X}^{(n)}\big | \cdot \mathbb {1}_{\mathcal {E}_K^c}\left( \mathbf {A}^{(n)}\right) \right]&\le \frac{\varepsilon }{3}&\mathbb {E}\left[ g(\mathbf{A})\mathbb {1}_{\mathcal {E}_K^c}\left( \mathbf {A}\right) \right]&\le \frac{\varepsilon }{3}, \end{aligned}$$

where $\mathbf {A}$ is distributed as the spectrum of a ${\text {GUE}}^0_d$ matrix. For the bound on the second expectation value, recall that the density of the eigenvalues $(\mu ,\ldots ,\mu _d)$ of a ${\text {GUE}}^0_d$ matrix is proportional to $\exp (-\sum _{i=1}^d \mu _i^2) \prod _{i<j}(\mu _i-\mu _j)^2$, and hence decays exponentially in $\Vert \mu \Vert _\infty$. By Lemma 4.4, $\lim _{n\rightarrow \infty }\mathbb {E}\left[ \mathbf {X}^{(n)}\mathbb {1}_{\mathcal {E}_K}\left( \mathbf {A}^{(n)}\right) \right] = \mathbb {E}\left[ g(\mathbf{A})\mathbb {1}_{\mathcal {E}_K}\left( \mathbf {A}\right) \right]$. Thus, we can choose $n_0\in \mathbb {N}$ such that for all $n\ge n_0$,

$$\begin{aligned} \left| \mathbb {E}\left[ \mathbf {X}^{(n)}\mathbb {1}_{\mathcal {E}_K}\left( \mathbf {A}^{(n)}\right) \right] - \mathbb {E}\left[ g(\mathbf{A})\mathbb {1}_{\mathcal {E}_K}\left( \mathbf {A}\right) \right] \right| \le \frac{\varepsilon }{3}. \end{aligned}$$

Using the above choices, we then have

$$\begin{aligned} \left| \mathbb {E}\big [\mathbf {X}^{(n)}\big ] - \mathbb {E}\left[ g(\mathbf{A})\right] \right|&\le \mathbb {E}\left[ \big |\mathbf {X}^{(n)}\big | \cdot \mathbb {1}_{\mathcal {E}_K^c}\left( \mathbf {A}^{(n)}\right) \right] + |\mathbb {E}\left[ g(\mathbf{A})\mathbb {1}_{\mathcal {E}_K^c}\left( \mathbf {A}\right) \right] |\\&+ \left| \mathbb {E}\left[ \mathbf {X}^{(n)}\mathbb {1}_{\mathcal {E}_K}\left( \mathbf {A}^{(n)}\right) \right] - \mathbb {E}\left[ g(\mathbf{A})\mathbb {1}_{\mathcal {E}_K}\left( \mathbf {A}\right) \right] \right| \le \varepsilon \end{aligned}$$

for all $n\ge n_0$, proving the desired convergence of the expectation values. $\square$

From Theorem 4.1 we immediately obtain the following corollary about uniform integrability of the moments of $\mathbf {A}$.

### Corollary 4.5

Let $k\in \mathbb {N}$, let $j\in \{1,\ldots ,d\}$, and, for every *n*, let $\mathbf{A}^{(n)}$ be the random vector defined in (4.3). Then, the sequence of *k*th moments $\big \lbrace ( \mathbf{A}^{(n)}_j)^k \big \rbrace _{n\in \mathbb {N}}$ is uniformly integrable and $\lim _{n\rightarrow \infty } \mathbb {E}\bigl [(\mathbf{A}^{(n)}_j)^k\bigr ] = \mathbb {E}[\mathbf{A}_j^k]$, where $\mathbf{A} \sim {\text {GUE}}^0_d$.

## Probabilistic PBT

Our goal in this section is to determine the asymptotics of $p^{\mathrm {EPR}}_d$ using the formula (3.9) and exploiting our convergence theorem, Theorem 4.1. The main result is the following theorem stated in Sect. 1.2, which we restate here for convenience.

### Theorem 1.3

(Restated). For probabilistic port-based teleportation in arbitrary but fixed dimension *d* with EPR pairs as resource states,

$$\begin{aligned} p^{\mathrm {EPR}}_d(N) = 1 - \sqrt{\frac{d}{N-1}} \mathbb {E}[\lambda _{\max }(\mathbf {G})] + o\left( N^{-1/2}\right) , \end{aligned}$$

where $$\mathbf {G}\sim {\text {GUE}}^0_d$$.

Previously, such a result was only known for $d=2$ following from an exact formula for $p^{\mathrm {EPR}}_2(N)$ derived in [4]. We show in Lemma C.1 in “Appendix C” that, for $d=2$, $\mathbb E[\lambda _{\max }(\mathbf {G})] = \frac{2}{\sqrt{\pi }}$, hence rederiving the asymptotics from [4].

While Theorem 1.3 characterizes the limiting behavior of $p^{\mathrm {EPR}}$ for large *N*, it contains the constant $\mathbb {E}[\lambda _{\max }(\mathbf {G})]$, which depends on *d*. As $\mathbb {E}[\mathbf {M}]=0$ for $\mathbf {M}\sim {\text {GUE}}_d$, it suffices to analyze the expected largest eigenvalue for ${\text {GUE}}_d$. The famous Wigner semicircle law [24] implies immediately that

$$\begin{aligned} \lim _{d\rightarrow \infty }\frac{\mathbb {E}[\lambda _{\max }(\mathbf {G})]}{\sqrt{d}}=2, \end{aligned}$$

but meanwhile the distribution of the maximal eigenvalue has been characterized in a much more fine-grained manner. In particular, according to [61], there exist constants *C* and $C'$ such that the expectation value of the maximal eigenvalue satisfies the inequalities

$$\begin{aligned} 1-\frac{1}{C'd^{\frac{2}{3}}}\le \frac{\mathbb {E}[\lambda _{\max }(\mathbf {G})]}{2\sqrt{d}}\le 1-\frac{1}{Cd^{\frac{2}{3}}}. \end{aligned}$$

This also manifestly reconciles Theorem 1.3 with the fact that teleportation needs at least $2 \log d$ bits of classical communication (see Sect. 7), since the amount of classical communication in a port-based teleportation protocol consists of $\log N$ bits.

### Proof of Theorem 1.3

We start with Eq. (3.9), which was derived in [21], and which we restate here for convenience:

$$\begin{aligned} p^{\mathrm {EPR}}_d(N) = \frac{1}{d^N}\sum _{\alpha \vdash N-1}m_{d,\alpha }^2\frac{d_{\mu ^*}}{m_{d,\mu ^*}}, \end{aligned}$$

where $\mu ^*$ is the Young diagram obtained from $\alpha \vdash N-1$ by adding one box such that $\gamma _\mu (\alpha ) = N\frac{m_{d,\mu } d_\alpha }{m_{d,\alpha },d_\mu }$ is maximal. By Lemma 3.7, we have $\gamma _\mu (\alpha ) = \alpha _i - i + d + 1$ for $\mu =\alpha +e_i$. This is maximal if we choose $i=1$, resulting in $\gamma _{\mu ^*}(\alpha ) = \alpha _1+d$. We therefore obtain:

$$\begin{aligned} p^{\mathrm {EPR}}_d(N)&= \frac{1}{d^N}\sum _{\alpha \vdash N-1}m_{d,\alpha } d_\alpha \frac{m_{d,\alpha } d_{\mu ^*}}{m_{d,\mu ^*} d_\alpha }\\&= \frac{1}{d^N}\sum _{\alpha \vdash N-1}m_{d,\alpha } d_\alpha \frac{N}{\gamma _{\mu ^*}(\alpha )}\\&= \frac{1}{d} \mathbb {E}_{\varvec{\alpha }}\left[ \frac{N}{\gamma _{\mu ^*}(\varvec{\alpha })}\right] \\&= \frac{1}{d} \mathbb {E}_{\varvec{\alpha }}\left[ \frac{N}{\varvec{\alpha }^{(N-1)}_1+d}\right] . \end{aligned}$$

Recall that

$$\begin{aligned} \varvec{\alpha }^{(N-1)} = \left( \varvec{\alpha }^{(N-1)}_1,\ldots ,\varvec{\alpha }^{(N-1)}_d \right) \sim p_{d,N-1} \end{aligned}$$

is a random vector corresponding to Young diagrams with $N-1$ boxes and at most *d* rows, where $p_{d,N-1}$ is the Schur–Weyl distribution defined in (4.1). We continue by abbreviating $n=N-1$ and changing to the centered and renormalized random variable $\mathbf {A}^{(n)}$ from Eq. (4.3). Corollary 4.5 implies that

5.1 $$\begin{aligned} \mathbb {E}\left[ \mathbf {A}^{(n)}_{1}\right]&\xrightarrow {N\rightarrow \infty } \mathbb {E}[\lambda _{\max }(\mathbf {G})]&\mathbb {E}\left[ \big (\mathbf {A}^{(n)}_{1}\big )^2\right]&\xrightarrow {N\rightarrow \infty } \mathbb {E}[\lambda _{\max }(\mathbf {G})^2]. \end{aligned}$$

Using the $\mathbf {A}^{(n)}$ variables from (4.3), linearity of the expectation value and suitable rearranging, one finds that

$$\begin{aligned} \sqrt{N - 1} \left( 1 - p^{\mathrm {EPR}}_d(N)\right)&= \mathbb {E}\left[ \sqrt{N-1} - \frac{\sqrt{N-1} N }{\sqrt{d(N-1)} \mathbf {A}^{(n)}_1 + N-1 + d^2}\right] \\&= \mathbb {E}\left[ f_{d,N}\left( \mathbf {A}^{(n)}_1\right) \right] , \end{aligned}$$

where we set

$$\begin{aligned} f_{d,N}(x) \,{:}{=}\, \frac{x \sqrt{d}+\frac{d^2-1}{\sqrt{N-1}}}{1 + \frac{d^2}{N-1} + \frac{x\sqrt{d}}{\sqrt{N-1}}}. \end{aligned}$$

Note that, for $x\ge 0$,

$$\begin{aligned} \left| f_{d,N}(x) - x \sqrt{d} \right|&= \left| \frac{\frac{d^2-1}{\sqrt{N-1}} + \frac{x d^{5/2}}{N-1} + \frac{x^2 d}{\sqrt{N-1}}}{1 + \frac{d^2}{N-1} + \frac{x\sqrt{d}}{\sqrt{N-1}}} \right| \\&\le \frac{d^2-1}{\sqrt{N-1}} + \frac{x d^{5/2}}{N-1} + \frac{x^2 d}{\sqrt{N-1}}\\&\le \frac{1}{\sqrt{N-1}} \left( K_1 + K_2 x + K_3 x^2\right) \end{aligned}$$

for some constants $K_i$, where the first inequality follows from the fact that the denominator in the first line is greater than 1 for $x\ge 0$. Since both $\mathbf {A}^{(n)}_1\ge 0$ and $\lambda _{\max }(\mathbf {G})\ge 0$, and using (5.1), it follows that

$$\begin{aligned}&\left| \mathbb {E}\left[ f_{d,N}\left( \mathbf {A}^{(n)}_1\right) \right] - \sqrt{d}\, \mathbb {E}[\lambda _{\max }(\mathbf {G})] \right| \\&\qquad \le \left| \mathbb {E}\left[ f_{d,N}\left( \mathbf {A}^{(n)}_1\right) \right] - \sqrt{d}\, \mathbb {E}\left[ \mathbf {A}^{(n)}_1\right] \right| + \sqrt{d} \left| \mathbb {E}\left[ \mathbf {A}^{(n)}_1\right] - \mathbb {E}[\lambda _{\max }(\mathbf {G})] \right| \\&\qquad \le \frac{K_1 + K_2 \mathbb {E}\left[ \mathbf {A}^{(n)}_1\right] + K_3 \mathbb {E}\left[ \big (\mathbf {A}^{(n)}_{1}\big )^2\right] }{\sqrt{N-1}} + \sqrt{d} \left|\mathbb {E}\left[ \mathbf {A}^{(n)}_1\right] - \mathbb {E}[\lambda _{\max }(\mathbf {G})] \right|\\&\qquad \xrightarrow {N\rightarrow \infty } 0. \end{aligned}$$

Thus we have shown that, for fixed *d* and large *N*,

$$\begin{aligned} p^{\mathrm {EPR}}_d(N) = 1 - \sqrt{\frac{d}{N-1}} \mathbb {E}[\lambda _{\max }(\mathbf {G})] + o\left( N^{-1/2}\right) , \end{aligned}$$

which is what we set out to prove. $\square$

### Remark 5.1

For the probabilistic protocol with optimized resource state, recall from Eq. (3.8) that

$$\begin{aligned} p^*_d(N)=1-\frac{d^2-1}{d^2-1+N} = 1 - \frac{d^2-1}{N} + o(1/N). \end{aligned}$$

For fixed *d*, this converges to unity as *O*(1/*N*), i.e., much faster than the $O(1/\sqrt{N})$ convergence in the EPR case proved in Theorem 1.3 above.

## Deterministic PBT

The following section is divided into two parts. First, in Sect. 6.1 we derive the leading order of the standard protocol for deterministic port-based teleportation (see Sect. 3, where this terminology is explained). Second, in Sect. 6.2 we derive a lower bound on the leading order of the optimal deterministic protocol. As in the case of probabilistic PBT, the optimal deterministic protocol converges quadratically faster than the standard deterministic protocol, this time displaying an $N^{-2}$ versus $N^{-1}$ behavior (as opposed to $N^{-1}$ versus $N^{-1/2}$ in the probabilistic case).

### Asymptotics of the standard protocol

Our goal in this section is to determine the leading order in the asymptotics of $F^{\mathrm {std}}_d$. We do so by deriving an expression for the quantity $\lim _{N\rightarrow \infty }N(1 - F^{\mathrm {std}}_d(N))$, that is, we determine the coefficient $c_1=c_1(d)$ in the expansion

$$\begin{aligned} F^{\mathrm {std}}_d(N) = 1 - \frac{c_1}{N} + o(N^{-1}) \,. \end{aligned}$$

We need the following lemma that states that we can restrict a sequence of expectation values in the Schur–Weyl distribution to a suitably chosen neighborhood of the expectation value and remove degenerate Young diagrams without changing the limit. Let

$$\begin{aligned} H(x)={\left\{ \begin{array}{ll} 0 &{}\quad x<0\\ 1 &{}\quad x\ge 0 \end{array}\right. } \end{aligned}$$

be the Heaviside step function. Recall the definition of the centered and normalized variables

$$\begin{aligned} A_i\,{:}{=}\, \frac{\alpha _i - n/d}{\sqrt{n/d}}, \end{aligned}$$

such that $\alpha _i = \sqrt{\frac{n}{d}} A_i + \frac{n}{d}$. In the following it will be advantageous to use both variables, so we use the notation $A(\alpha )$ and $\alpha (A)$ to move back and forth between them.

#### Lemma 6.1

Let $C>0$ be a constant and $0<\varepsilon <\frac{1}{2}(d-2)^{-1}$ (for $d=2$, $\varepsilon >0$ can be chosen arbitrary). Let $f_N$ be a function on the set of centered and rescaled Young diagrams (see Eq. (4.3)) that that grows at most polynomially in *N*, and for *N* large enough and all arguments *A* such that $\Vert A\Vert _1\le n^\varepsilon$ fulfills the bound

$$\begin{aligned} f_N(A)\le C N. \end{aligned}$$

Then the limit of its expectation values does not change when removing degenerate and large deviation diagrams,

$$\begin{aligned} \lim _{N\rightarrow \infty }\mathbb {E}_{\varvec{\alpha }}[f_N(\mathbf {A})]=\lim _{N\rightarrow \infty } \mathbb {E}_{\varvec{\alpha }}[f_N(\mathbf {A})H(n^\varepsilon -\Vert \mathbf {A}\Vert _1) \mathbb {1}_{\mathrm {ND}}(\mathbf {A})], \end{aligned}$$

where $\mathbb {1}_{\mathrm {ND}}$ is the indicator function that is 0 if two or more entries of its argument are equal, and 1 else. Moreover we have the stronger statement

$$\begin{aligned} \left| \mathbb {E}_{\varvec{\alpha }}[f_N(\mathbf {A})]-\mathbb {E}_{\varvec{\alpha }} [f_N(\mathbf {A})H(n^\varepsilon -\Vert \mathbf {A}\Vert _1)\mathbb {1}_{\mathrm {ND}}(\mathbf {A})]\right| =O(N^{-1/2+(d-2)\varepsilon }). \end{aligned}$$

#### Proof

The number of all Young diagrams is bounded from above by a polynomial in *N*. But $p_{d, n}(\alpha (A))=O(\exp (-\gamma \Vert A\Vert _1^2))$ for some $\gamma >0$ according to Lemma 4.2, which implies that

$$\begin{aligned} \lim _{N\rightarrow \infty }\mathbb {E}_{\varvec{\alpha }}[f_N(\mathbf {A})]=\lim _{N\rightarrow \infty }\mathbb {E}_{\varvec{\alpha }}[f_N(\mathbf {A})H(n^\varepsilon -\Vert \mathbf {A}\Vert _1)]. \end{aligned}$$

Let us now look at the case of degenerate diagrams. Define the set of degenerate diagrams that are also in the support of the above expectation value,

$$\begin{aligned} \Xi&=\left\{ \alpha \vdash _{d}n:\exists \, 1\le i\le d-1 \text { s.t. }\alpha _i=\alpha _{i+1}\wedge \left( \frac{n}{d}\right) ^{-1/2}\left\| \alpha -\frac{n}{d}\mathbf {1}\right\| _1\le n^\varepsilon \right\} \\&=\mathrm {ND}(d,n)^c\cap \mathrm {supp}(H(n^\varepsilon -\Vert A\Vert _1)). \end{aligned}$$

Here, $\mathbf {1}=(1,\ldots ,1)^T\in \mathbb {R}^d$ is the all-one vector. We write

6.1 $$\begin{aligned} \Xi =\bigcup _{k=1}^{d-1}\Xi _k \end{aligned}$$

with

$$\begin{aligned} \Xi _k=\left\{ \alpha \vdash _{d}n:\alpha _k=\alpha _{k+1}\wedge \left( \frac{n}{d}\right) ^{-1/2}\left\| \alpha -\frac{n}{d}\mathbf {1}\right\| _1\le n^\varepsilon \right\} . \end{aligned}$$

It suffices to show that

$$\begin{aligned} \lim _{N\rightarrow \infty }\mathbb {E}_{\varvec{\alpha }}[f(A(\varvec{\alpha })) H(n^\varepsilon -\Vert A(\varvec{\alpha })\Vert _1)\mathbb {1}_{\Xi _k}(\varvec{\alpha })]=0 \end{aligned}$$

for all $k=1,\ldots ,d-1$. We can now apply Lemma 4.2 to $\Xi _k$ and choose the constants $\gamma _k=0$ and $\gamma _i=\frac{1}{2}+\varepsilon$ for $i\ne k$. Using (4.11), the 1-norm condition on *A* and bounding the exponential function by a constant we therefore get the bound

$$\begin{aligned} p_{d,n}(\alpha (A))\le C_1 n^{-\frac{d+1}{2}} \end{aligned}$$

for some constant $C_1>0$. The cardinality of $\Xi _k$ is not greater than the number of integer vectors whose entries are between $n/d-n^{1/2+\varepsilon }$ and $n/d+n^{1/2+\varepsilon }$ and sum to *n*, and for which the *k*th and $(k+1)$st entries are equal. It therefore holds that

$$\begin{aligned} \left| \Xi _k\right| \le C_2 n^{(d-2)\left( \frac{1}{2} +\varepsilon \right) }. \end{aligned}$$

By assumption,

$$\begin{aligned} f(A)\le C n \quad \text {for all }A\text { such that }\alpha (A)\in \Xi _k. \end{aligned}$$

Finally, we conclude that

$$\begin{aligned} \mathbb {E}_{\varvec{\alpha }}[f(A(\varvec{\alpha }))H(n^\varepsilon - \Vert A(\varvec{\alpha })\Vert _1)\mathbb {1}_{\Xi _k}(\varvec{\alpha })]&\le CC_1C_2n\cdot n^{-\frac{d+1}{2}}n^{(d-2)\left( \frac{1}{2} +\varepsilon \right) }\\&\le \tilde{C} n^{(d-2)\varepsilon -\frac{1}{2}}. \end{aligned}$$

This implies that we have indeed that

$$\begin{aligned} \lim _{N\rightarrow \infty }\mathbb {E}_{\varvec{\alpha }}[f(A(\varvec{\alpha })) H(n^\varepsilon -\Vert A(\varvec{\alpha })\Vert _1)\mathbb {1}_{\Xi _k}(\varvec{\alpha })]=0. \end{aligned}$$

In fact, we obtain the stronger statement

$$\begin{aligned} \left| \mathbb {E}_{\varvec{\alpha }}[f(A(\varvec{\alpha }))H(n^\varepsilon - \Vert A(\varvec{\alpha })\Vert _1)\mathbb {1}_{\Xi _k}(\varvec{\alpha })]\right| =O(N^{-1/2+(d-2)\varepsilon }). \end{aligned}$$

The statement follows now using Eq. (6.1). $\quad \square$

With Lemma 6.1 in hand, we can now prove the main result of this section, which we stated in Sect. 1.2 and restate here for convenience.

#### Theorem 1.2

(Restated). For arbitrary but fixed *d* and any $\delta >0$, the entanglement fidelity of the standard protocol of PBT is given by

$$\begin{aligned} F^{\mathrm {std}}_d(N)=1-\frac{d^2-1}{4N}+O(N^{-\frac{3}{2}+\delta }). \end{aligned}$$

#### Proof

We first define $n=N-1$ and recall (3.5), which we can rewrite as follows:

$$\begin{aligned} F^{\mathrm {std}}_d(N)&=d^{-N-2}\sum _{\alpha \vdash _d n}\left( \sum _{\mu =\alpha +\square }\sqrt{d_\mu m_{d,\mu }}\right) ^2\\&=d^{-N-2}\sum _{\alpha \vdash _d n}d_\alpha m_{d,\alpha }\left( \sum _{\mu =\alpha +\square }\frac{m_{d,\mu }}{m_{d,\alpha }}\sqrt{\frac{d_\mu m_{d,\alpha }}{m_{d,\mu } d_\alpha }}\right) ^2\\&=d^{-N-2}\sum _{\alpha \vdash _d n}d_\alpha m_{d,\alpha }\left( \sum _{\mu =\alpha +e_i\text { YD}}\left[ \prod _{j:j\ne i}\frac{\alpha _i-\alpha _j+j-i+1}{\alpha _i-\alpha _j+j-i}\right] \sqrt{\frac{N}{\mu _i-i+d}} \right) ^2 \\&=\frac{1}{d^3} \mathbb {E}_{\varvec{\alpha }}\left[ \left( \sum _{\varvec{\mu } =\varvec{\alpha }+e_i\text { YD}}\left[ \prod _{j:j\ne i}\frac{\varvec{\alpha }^{(n)}_i-\varvec{\alpha }^{(n)}_j+j-i+1}{\varvec{\alpha }^{(n)}_i-\varvec{\alpha }^{(n)}_j+j-i}\right] \sqrt{\frac{N}{\varvec{\mu }^{(N)}_i-i+d}}\right) ^2\right] \\&=\frac{1}{d^3} \mathbb {E}_{\varvec{\alpha }}\left[ \left( \sum _{\varvec{\mu }=\varvec{\alpha }+e_i\text { YD}}\left[ \prod _{j:j\ne i} \left( 1 + \frac{1}{\varvec{\alpha }^{(n)}_i-\varvec{\alpha }^{(n)}_j+j-i} \right) \right] \sqrt{\frac{N}{\varvec{\mu }^{(N)}_i-i+d}}\right) ^2\right] . \end{aligned}$$

In the third step, we used Lemma 3.7 for the term $\frac{d_\mu m_{d,\alpha }}{m_{d,\mu } d_\alpha }$ and the Weyl dimension formula (2.3) for the term $\frac{m_{d,\mu }}{m_{d,\alpha }}$. The expectation value refers to a random choice of $\alpha \vdash _{d}n$ according to the Schur–Weyl distribution $p_{d,n}$. The sum over $\mu =\alpha +e_i$ is restricted to only those $\mu$ that are valid Young diagrams, i.e., where $\alpha _{i-1}>\alpha _i$, which we indicate by writing ‘YD’. Hence, we have

6.2 $$\begin{aligned}&N(1 - F^{\mathrm {std}}_d(N))\nonumber \\&\quad =\frac{N}{d^2} \mathbb {E}_{\varvec{\alpha }}\left[ d^2 - \left( \sum _{\varvec{\mu }=\varvec{\alpha }+e_i\text { YD}}\left[ \prod _{j\ne i} \left( 1 + \frac{1}{\varvec{\alpha }^{(n)}_i-\varvec{\alpha }^{(n)}_j+j-i} \right) \right] \sqrt{\frac{N/d}{\varvec{\mu }^{(N)}_i-i+d}}\right) ^2\right] .\nonumber \\ \end{aligned}$$

In the following, we suppress the superscript indicating $n=N-1$ for the sake of readability. The random variables $\varvec{\alpha }$, $\mathbf {A}$, and $\varvec{\Gamma }_{ij}$, as well as their particular values $\alpha$, *A*, and $\Gamma _{ij}$, are all understood to be functions of $n=N-1$.

The function

$$\begin{aligned} f_N( A)\,{:}{=}\,\frac{N}{d^2} \left( d^2 - \left( \sum _{{\mu }={\alpha }( A)+e_i\text { YD}}\left[ \prod _{j:j\ne i} \left( 1 + \frac{1}{{\alpha }_i(A)-{\alpha }_j(A)+j-i} \right) \right] \sqrt{\frac{N/d}{{\mu }_i-i+d}}\right) ^2\right) \end{aligned}$$

satisfies the requirements of Lemma 6.1. Indeed we have that

$$\begin{aligned} \frac{1}{{\alpha }_i(A)-{\alpha }_j(A)+j-i} \le 1 \end{aligned}$$

for all $i\ne j$, and clearly

$$\begin{aligned} \sqrt{\frac{N/d}{{\mu }_i-i+d}}\le \sqrt{N}. \end{aligned}$$

Therefore we get

$$\begin{aligned} f_N( A)\le C N^2 \end{aligned}$$

for some constant *C*. If $\Vert A\Vert _1\le n^\varepsilon$, we have that

$$\begin{aligned} \sqrt{\frac{N/d}{{\mu }_i-i+d}}\le \sqrt{\frac{N/d}{n/d-n^\varepsilon }}\le \sqrt{N/n}+O\left( n^{-(1-\varepsilon )}\right) \end{aligned}$$

and hence

$$\begin{aligned} f_N( A)\le C N \end{aligned}$$

for *N* large enough. We therefore define, using an $\varepsilon$ in the range given by Lemma 6.1, the modified expectation value

6.3 $$\begin{aligned} \tilde{\mathbb {E}}_{\varvec{\alpha }}[f(\varvec{\alpha })]\,{:}{=}\, \mathbb {E}_{\varvec{\alpha }}[f(\varvec{\alpha })\mathbb {1}_{\mathrm {ND}(n,d)}(\varvec{\alpha })H(n^\varepsilon -\Vert A(\varvec{\alpha })\Vert _1)], \end{aligned}$$

and note that an application of Lemma 6.1 shows that the limit that we are striving to calculate does not change when replacing the expectation value with the above modified expectation value, and the difference between the members of the two sequences is $O(n^{-\frac{1}{2}+\varepsilon (d-2)})$.

For a non-degenerate $\alpha$, adding a box to any row yields a valid Young diagram $\mu$. Hence, the sum $\sum _{\mu =\alpha +e_i\text { YD}}$ in (6.2) can be replaced by $\sum _{i=1}^d$, at the same time replacing $\mu _i$ with $\alpha _i+1$. The expression in (6.2) therefore simplifies to

$$\begin{aligned} R_N&\,{:}{=}\,\frac{N}{d^2} \tilde{\mathbb {E}}_{\varvec{\alpha }}\left[ d^2 - \left( \sum _{i=1}^d\left[ \prod _{k:k\ne i} \left( 1 + \frac{1}{\varvec{\alpha }_i-\varvec{\alpha }_k+k-i} \right) \right] \sqrt{\frac{N/d}{\varvec{\alpha }_i+1-i+d}}\right) ^2\right] . \end{aligned}$$

Let us look at the square root term, using the variables $\mathbf {A}_i$. For sufficiently large *n*, we write

$$\begin{aligned} \sqrt{\frac{N/d}{\varvec{\alpha }_i+1-i+d}}&=\sqrt{N/n} \left( 1+\frac{(1-i+d)d}{n}+\sqrt{\frac{d}{n}}\mathbf {A}_i\right) ^{-1/2}\\&=\sqrt{\frac{N\gamma _{i,d,n}}{n}}\left( 1+\gamma _{i,d,n}\sqrt{\frac{d}{n}}\mathbf {A}_i\right) ^{-1/2}\\&=\sqrt{\frac{N\gamma _{i,d,n}}{n}}\sum _{r=0}^\infty a_r \left( \gamma _{i,d,n}\sqrt{\frac{d}{n}}\mathbf {A}_i\right) ^r. \end{aligned}$$

In the second line we have defined

$$\begin{aligned} \gamma _{i,d,n}=\left( 1+\frac{(1-i+d)d}{n}\right) ^{-1}, \end{aligned}$$

and in the third line we have written the inverse square root in terms of its power series around 1. This is possible as we have $\Vert \mathbf {A}\Vert _1\le n^\varepsilon$ on the domain of $\tilde{\mathbb {E}}$, so $\gamma _{i,d,n}\sqrt{\frac{d}{n}}\mathbf {A}_i=O(n^{-1/2+\varepsilon })$, i.e., it is in particular in the convergence radius of the power series, which is equal to 1. This implies also that the series converges absolutely in that range. Defining

$$\begin{aligned} \varvec{\Gamma }_{ik}=-\varvec{\Gamma }_{ki}=\mathbf {A}_i-\mathbf {A}_k +\frac{k-i}{\sqrt{\frac{n}{d}}} \end{aligned}$$

as in Sect. 4, we can write

$$\begin{aligned} R_N&=\frac{N}{d^2} \tilde{\mathbb {E}}_{\varvec{\alpha }}\Bigg [d^2 - \frac{N}{n}\sum _{i,j=1}^d\sqrt{\gamma _{i,d,n}\gamma _{j,d,n}}\left[ \prod _{k:k< i} \left( 1 -\sqrt{\frac{d}{n}}\varvec{\Gamma }_{ki}^{-1} \right) \right] \\&\qquad \left[ \prod _{k:k> i} \left( 1 +\sqrt{\frac{d}{n}}\varvec{\Gamma }_{ik}^{-1} \right) \right] \\&\qquad \left[ \prod _{l:l< j} \left( 1 -\sqrt{\frac{d}{n}}\varvec{\Gamma }_{lj}^{-1} \right) \right] \left[ \prod _{l:l> j} \left( 1 +\sqrt{\frac{d}{n}}\varvec{\Gamma }_{jl}^{-1} \right) \right] \\&\quad \quad \left( \sum _{r=0}^\infty a_r \left( \gamma _{i,d,n}\sqrt{\frac{d}{n}}\mathbf {A}_i\right) ^r\right) \left( \sum _{r=0}^\infty a_r \left( \gamma _{j,d,n}\sqrt{\frac{d}{n}}\mathbf {A}_j\right) ^r\right) \Bigg ]\\&{=}{:}\,\frac{N}{d^2} \tilde{\mathbb {E}}_{\varvec{\alpha }}\left[ d^2- \frac{N}{n}\sum _{i,j=1}^d\sqrt{\gamma _{i,d,n}\gamma _{j,d,n}} \left( \sum _{s=0}^{2(d-1)}\left( \frac{d}{n}\right) ^{\frac{s}{2}}P_{i,j}^{(1,s)} \left( \Gamma ^{-1}\right) \right) \right. \\&\qquad \left. \left( \sum _{r=0}^{\infty }\left( \frac{d}{n} \right) ^{\frac{r}{2}}P^{(2,r)}_{i,j}(\tilde{\mathbf {A}})\right) \right] . \end{aligned}$$

Here we have defined $\tilde{ \mathbf {A}}$ by $\tilde{ \mathbf {A}}_i=\gamma _{i,d,n}\mathbf {A}_i$ and the polynomials $P_{i,j}^{(1,s)}$, $P_{i,j}^{(2,r)}$, for $s=0,\ldots ,2(d-1)$, $r\in \mathbb {N}$, $i,j=1,\ldots ,d$, which are homogeneous of degree *r*, and *s*, respectively. In the last equality we have used the absolute convergence of the power series. We have also abbreviated $\varvec{\Gamma }\,{:}{=}\,(\varvec{\Gamma }_{ij})_{i<j}$, $\varvec{\Gamma }^{-1}$ is to be understood elementwise, and $P_{i,j}^{(1,s)}$ has the additional property that for all $k,l\in \{1,\ldots ,d\}$ it has degree at most 2 in each variable $\varvec{\Gamma }_{k,l}$.

By the Fubini-Tonelli Theorem, we can now exchange the infinite sum and the expectation value if the expectation value

$$\begin{aligned} \tilde{\mathbb {E}}_{\varvec{\alpha }}\left[ \left( \sum _{s=0}^{2(d-1)}\left( \frac{d}{n}\right) ^{\frac{s}{2}}\tilde{P}_{i,j}^{(1,s)}\left( |\varvec{\Gamma }^{-1}|\right) \right) \left( \sum _{r=0}^{\infty }\left( \frac{d}{n}\right) ^{\frac{r}{2}}\tilde{P}^{(2,r)}_{i,j}(|\tilde{ \mathbf {A}}|)\right) \right] \end{aligned}$$

exists, where the polynomials $\tilde{P}^{(1,s)}_{i,j}$ and $\tilde{P}^{(2,r)}_{i,j}$ are obtained from $P^{(1,s)}_{i,j}$ and $P^{(2,r)}_{i,j}$, respectively, by replacing the coefficients with their absolute value, and the absolute values $|\varvec{\Gamma }^{-1}|$ and $|\tilde{\mathbf {A}}|$ are to be understood element-wise. But the power series of the square root we have used converges absolutely on the range of $\mathbf {A}$ restricted by $\tilde{\mathbb {E}}$ (see Eq. (6.3)), yielding a continuous function on an appropriately chosen compact interval. Moreover, if *A* is in the range of $\mathbf {A}$ restricted by $\tilde{\mathbb {E}}$, then so is |*A*|. The function is therefore bounded, as is $\tilde{ \mathbf {A}}$ for fixed *N*, and the expectation value above exists. We therefore get

$$\begin{aligned} R_N&=\frac{N}{d^2}\left[ d^2-\frac{N}{n}\sum _{i,j=1}^d\sqrt{\gamma _{i,d,n}\gamma _{j,d,n}} \sum _{s=0}^{2(d-1)}\sum _{r=0}^{\infty }\left( \frac{d}{n}\right) ^{\frac{s+r}{2}} \tilde{\mathbb {E}}_{\varvec{\alpha }}\left[ P_{i,j}^{(1,s)}\left( \varvec{\Gamma }^{-1}\right) P^{(2,r)}_{i,j}(\tilde{ \mathbf {A}})\right] \right] . \end{aligned}$$

Now note that the expectation values above have the right form to apply Theorem 4.1, so we can start calculating expectation values provided that we can exchange the limit $N\rightarrow \infty$ with the infinite sum. We can then split up the quantity $\lim _{N\rightarrow \infty }R_N$ as follows,

6.4 $$\begin{aligned} \lim _{N\rightarrow \infty }R_N&=\lim _{N\rightarrow \infty }\frac{N}{d^2}\left[ d^2-\frac{N}{n}\sum _{i,j=1}^d\sqrt{\gamma _{i,d,n}\gamma _{j,d,n}}\sum _{s=0}^{2(d-1)}\sum _{r=0}^{\infty }\left( \frac{d}{n}\right) ^{\frac{s+r}{2}}\right. \nonumber \\&\quad \left. \tilde{\mathbb {E}}_{\varvec{\alpha }}\left[ P_{i,j}^{(1,s)}\left( \varvec{\Gamma }^{-1}\right) P^{(2,r)}_{i,j}(\tilde{\mathbf {A}})\right] \right] \nonumber \\&=\lim _{N\rightarrow \infty }\frac{N^2}{nd^2}\tilde{\mathbb {E}}_{\varvec{\alpha }}\left[ \frac{d^2 n}{N}-\sum _{i,j=1}^d\sqrt{\gamma _{i,d,n}\gamma _{j,d,n}}P_{i,j}^{(1,0)}\left( \varvec{\Gamma }^{-1}\right) P^{(2,0)}_{i,j}(\tilde{ \mathbf {A}})\right] \end{aligned}$$

6.5 $$\begin{aligned}&\quad -\sum _{r,s\in \mathbb {N}: 1\le r+s\le 2}\lim _{N\rightarrow \infty }\frac{N^2}{nd^2}\left( \frac{d}{n}\right) ^{\frac{r+s}{2}}\nonumber \\&\qquad \tilde{\mathbb {E}}_{\varvec{\alpha }}\left[ \sum _{i,j=1}^d\sqrt{\gamma _{i,d,n}\gamma _{j,d,n}}P_{i,j}^{(1,s)} \left( \varvec{\Gamma }^{-1}\right) P^{(2,r)}_{i,j}(\tilde{ \mathbf {A}})\right] \end{aligned}$$

6.6 $$\begin{aligned}&\quad -\lim _{N\rightarrow \infty }\sum _{\begin{array}{c} r,s\in \mathbb {N}\\ r+s\ge 3\\ s\le 2(d-1) \end{array}}\frac{N^2}{nd^2}\left( \frac{d}{n}\right) ^{\frac{r+s}{2}}\nonumber \\&\qquad \tilde{\mathbb {E}}_{\varvec{\alpha }}\left[ \sum _{i,j=1}^d\sqrt{\gamma _{i,d,n}\gamma _{j,d,n}}P_{i,j}^{(1,s)} \left( \varvec{\Gamma }^{-1}\right) P^{(2,r)}_{i,j}(\tilde{ \mathbf {A}})\right] , \end{aligned}$$

provided that all the limits on the right hand side exist. We continue by determining these limits and begin with Eq. (6.6). First observe that, for fixed *r* and *s* such that $r+s\ge 3$,

6.7 $$\begin{aligned} \lim _{N\rightarrow \infty }\frac{N^2}{nd^2}\left( \frac{d}{n}\right) ^{\frac{r+s}{2}} \tilde{\mathbb {E}}_{\varvec{\alpha }}\left[ \sum _{i,j=1}^d\sqrt{\gamma _{i,d,n}\gamma _{j,d,n}}P_{i,j}^{(1,s)}\left( \varvec{\Gamma }^{-1}\right) P^{(2,r)}_{i,j}(\tilde{ \mathbf {A}})\right] =0. \end{aligned}$$

This is because the expectation value in Eq. (6.7) converges according to Theorem 4.1 and Lemma 6.1, which in turn implies that the whole expression is $O(N^{-1/2})$. In particular, there exists a constant $K>0$ such that, for the finitely many values of *r* and *s* such that $r\le r_0\,{:}{=}\, \lceil (\frac{1}{2}-\varepsilon )^{-1}\rceil$,

$$\begin{aligned} \frac{N^2}{nd^2}\left( \frac{d}{n}\right) ^{\frac{r+s}{2}} \tilde{\mathbb {E}}_{\varvec{\alpha }}\left[ \sum _{i,j=1}^d\sqrt{\gamma _{i,d,n}\gamma _{j,d,n}}P_{i,j}^{(1,s)}\left( \varvec{\Gamma }^{-1}\right) P^{(2,r)}_{i,j}(\tilde{ \mathbf {A}})\right] \le K \quad (\forall N). \end{aligned}$$

Now suppose that $r>r_0$. On the domain of $\tilde{\mathbb {E}}$, we have $\Vert \mathbf {A}\Vert _1\le n^\varepsilon$. Therefore, we can bound

6.8 $$\begin{aligned} \frac{N^2}{nd}\left( \frac{d}{n}\right) ^{\frac{s+r}{2}} P_{i,j}^{(1,s)}\left( \varvec{\Gamma }^{-1}\right) P^{(2,r)}_{i,j}(\tilde{ \mathbf {A}})\le C N^{1+r(\varepsilon -1/2)}\le C \, 2^{1+r(\varepsilon -1/2)} \quad (\forall N) \end{aligned}$$

The first step holds because $\left( \frac{d}{n}\right) ^{\frac{s}{2}} P_{i,j}^{(1,s)}\left( \varvec{\Gamma }^{-1}\right)$ is a polynomial in the variables $\left( \frac{d}{n}\right) ^{\frac{1}{2}} \varvec{\Gamma }^{-1}_{ij}\le 1$ with coefficients independent of *n*, and in the second step we used that $1+r(\varepsilon -1/2) < 0$. We can therefore apply the dominated convergence theorem using the dominating function

$$\begin{aligned} g(r,s)={\left\{ \begin{array}{ll} K&{}\quad r\le r_0 = \lceil (\frac{1}{2}-\varepsilon )^{-1}\rceil \\ C \cdot 2^{1+r(\varepsilon -1/2)} &{}\quad \text {else} \end{array}\right. } \end{aligned}$$

to exchange the limit and the sum in Eq. (6.6). Thus, Eq. (6.7) implies that Eq. (6.6) is zero.

It remains to compute the limits Eqs. (6.4) and (6.5), i.e., the terms

$$\begin{aligned} T_{s,r} \,{:}{=}\, \lim _{N\rightarrow \infty }\frac{N^2}{nd^2}\left( \frac{d}{n} \right) ^{\frac{s+r}{2}} \tilde{\mathbb {E}}_{\varvec{\alpha }}\left[ \delta _{s0}\delta _{r0} \frac{d^2 n}{N}-\sum _{i,j=1}^d\sqrt{\gamma _{i,d,n}\gamma _{j,d,n}}P_{i,j}^{(1,s)}\left( \varvec{\Gamma }^{-1}\right) P^{(2,r)}_{i,j}(\tilde{ \mathbf {A}})\right] . \end{aligned}$$

for $r+s=0,1,2$. The first few terms of the power series for the inverse square root are given by

$$\begin{aligned} (1+x)^{-1/2}=1-\frac{x}{2}+\frac{3x^2}{8}+O(x^3). \end{aligned}$$

The relevant polynomials to calculate the remaining limits are, using the above and $-\varvec{\Gamma }_{ik}=\varvec{\Gamma }_{ki}$,

$$\begin{aligned} P^{(1,0)}_{i,j}&=P^{(1,0)}_{i,j}\equiv 1\\ P^{(1,1)}_{i,j}(\varvec{\Gamma }^{-1})&=-\sum _{k:k< i}\varvec{\Gamma }_{k:ki}^{-1} +\sum _{k> i} \varvec{\Gamma }_{ik}^{-1} -\sum _{l:l< j} \varvec{\Gamma }_{lj}^{-1} +\sum _{l:l> j}\varvec{\Gamma }_{jl}^{-1} \\&=\sum _{k:k\ne i}\varvec{\Gamma }_{ik}^{-1}+\sum _{l:l\ne j} \varvec{\Gamma }_{jl}^{-1}\\ P^{(2,1)}_{i,j}(\tilde{\mathbf {A}})&=a_1a_0(\tilde{\mathbf {A}}_i+\tilde{\mathbf {A}}_j)=-\frac{1}{2} (\tilde{\mathbf {A}}_i+\tilde{\mathbf {A}}_j)\\ P^{(1,2)}_{i,j}(\varvec{\Gamma }^{-1})&=\sum _{k,l:i\ne k\ne l\ne i} \varvec{\Gamma }_{ik}^{-1} \varvec{\Gamma }_{il}^{-1}+\sum _{k,l:j\ne k\ne l\ne j}\mathbf \Gamma _{ik}^{-1} \varvec{\Gamma }_{il}^{-1}+\sum _{k,l:i\ne k, l\ne j} \varvec{\Gamma }_{ik}^{-1} \varvec{\Gamma }_{il}^{-1}\\ P^{(2,2)}_{i,j}(\tilde{\mathbf {A}})&=a_2a_0(\tilde{\mathbf {A}}_i^2+\tilde{\mathbf {A}}_j^2)+a_1^2 \tilde{\mathbf {A}}_i\tilde{\mathbf {A}}_j=\frac{3}{8}(\tilde{\mathbf {A}}_i^2+\tilde{\mathbf {A}}_j^2)+\frac{1}{4} \tilde{\mathbf {A}}_i\tilde{\mathbf {A}}_j. \end{aligned}$$

We now analyze the remaining expectation values using these explicit expressions for the corresponding polynomials.

### Evaluating $T_{0,0}$

Using the power series expansions

$$\begin{aligned} \sqrt{\gamma _{i,d,n}}=1-\frac{d(d+1-i)}{2n}+O(n^{-2})=1-O(n^{-1}), \end{aligned}$$

we simplify

6.9 $$\begin{aligned} T_{0,0}&= \lim _{N\rightarrow \infty }\frac{N^2}{nd^2} \tilde{\mathbb {E}}_{\varvec{\alpha }}\left[ \frac{d^2 n}{N}-\sum _{i,j=1}^d\sqrt{\gamma _{i,d,n}\gamma _{j,d,n}}P_{i,j}^{(1,0)}\left( \varvec{\Gamma }^{-1}\right) P^{(2,0)}_{i,j}(\tilde{ \mathbf {A}})\right] \nonumber \\&=\lim _{N\rightarrow \infty }\frac{N^2}{nd^2}\left( \frac{d^2 n}{N}- \sum _{i,j=1}^d\left( 1-\frac{d(d+1-i)}{2n}-\frac{d(d+1-j)}{2n}+O(n^{-2})\right) \right) \nonumber \\&=\lim _{N\rightarrow \infty }\left( N- \frac{N^2}{n}+\frac{N^2}{nd^2}\sum _{i,j=1}^d\left( \frac{d(2d+2-i-j)}{2n}+O(n^{-2})\right) \right) \nonumber \\&=\lim _{N\rightarrow \infty }\left( - \frac{n+1}{n}+\frac{N^2}{2n^2}\left( 2d^2+2d-d(d+1)\right) +O(n^{-1})\right) \nonumber \\&=\frac{d(d+1)}{2}-1. \end{aligned}$$

In the second-to-last line we have replaced $N=n+1$.

### Evaluating $T_{0,1}$ and $T_{1,0}$

We first compute

$$\begin{aligned}&\sum _{i,j=1}^d\sqrt{\gamma _{i,d,n}\gamma _{j,d,n}}P_{i,j}^{(1,1)} \left( \varvec{\Gamma }^{-1}\right) P^{(2,0)}_{i,j}(\tilde{ \mathbf {A}})\\&\quad =\sum _{i,j=1}^d\sqrt{\gamma _{i,d,n}\gamma _{j,d,n}}\left( \sum _{k:k\ne i} \varvec{\Gamma }_{ik}^{-1}+\sum _{l:l\ne j} \varvec{\Gamma }_{jl}^{-1}\right) \\&\quad =\sum _{i,j=1}^d(1+O(n^{-1}))\left( \sum _{k:k\ne i}\varvec{\Gamma }_{ik}^{-1}+\sum _{l:l\ne j} \varvec{\Gamma }_{jl}^{-1}\right) \\&\quad =\sum _{i,j=1}^dO(n^{-1})\left( \sum _{k:k\ne i}\varvec{\Gamma }_{ik}^{-1}+\sum _{l:l\ne j} \varvec{\Gamma }_{jl}^{-1}\right) . \end{aligned}$$

In the last equation we have used that

$$\begin{aligned} \sum _{i\ne k}\varvec{\Gamma }_{ik}^{-1}=0, \end{aligned}$$

as the summation domain is symmetric in *i* and *k*, and $\mathbf \Gamma _{ik}^{-1}=- \mathbf \Gamma _{ki}^{-1}$. But now we can determine the limit,

6.10 $$\begin{aligned} T_{1,0}&= \lim _{N\rightarrow \infty }\frac{N^2}{nd^2}\left( \frac{d}{n}\right) ^{\frac{1}{2}} \tilde{\mathbb {E}}_{\varvec{\alpha }}\left[ \sum _{i,j=1}^d\sqrt{\gamma _{i,d,n}\gamma _{j,d,n}}P_{i,j}^{(1,1)}\left( \varvec{\Gamma }^{-1}\right) P^{(2,0)}_{i,j}(\tilde{ \mathbf {A}})\right] \nonumber \\&=\lim _{N\rightarrow \infty }\frac{N^2}{nd^2}\left( \frac{d}{n}\right) ^{\frac{1}{2}} \tilde{\mathbb {E}}_{\varvec{\alpha }}\left[ \sum _{i,j=1}^dO(n^{-1})\left( \sum _{k:k\ne i}\varvec{\Gamma }_{ik}^{-1}+\sum _{l:l\ne j} \varvec{\Gamma }_{jl}^{-1}\right) \right] \nonumber \\&=\sum _{i,j=1}^d\lim _{N\rightarrow \infty }O(n^{-1/2})\tilde{\mathbb {E}}_{\varvec{\alpha }}\left[ \left( \sum _{k:k\ne i}\varvec{\Gamma }_{ik}^{-1}+\sum _{l:l\ne j} \varvec{\Gamma }_{jl}^{-1}\right) \right] =0. \end{aligned}$$

Here we have used Theorem 4.1 to see that the sequence of expectation values converges, implying that the expression vanishes due to the $O(n^{-1/2})$ prefactor.

Similarly, to show that $T_{0,1}$ vanishes as well, we calculate

$$\begin{aligned}&\sum _{i,j=1}^d\sqrt{\gamma _{i,d,n}\gamma _{j,d,n}}P_{i,j}^{(1,0)}\left( \varvec{\Gamma }^{-1}\right) P^{(2,1)}_{i,j}(\tilde{ \mathbf {A}})\\&\quad =-\frac{1}{2}\sum _{i,j=1}^d\sqrt{\gamma _{i,d,n}\gamma _{j,d,n}} (\tilde{\mathbf {A}}_i+\tilde{\mathbf {A}}_j)\\&\quad =-\frac{1}{2}\sum _{i,j=1}^d\sqrt{\gamma _{i,d,n}\gamma _{j,d,n}} (\gamma _{i,d,n}{\mathbf {A}}_i+\gamma _{j,d,n}{\mathbf {A}}_j)\\&\quad =-\sum _{i,j=1}^d(1+O(n^{-1}))({\mathbf {A}}_i+{\mathbf {A}}_j)\\&\quad =-\sum _{i,j=1}^dO(n^{-1})({\mathbf {A}}_i+{\mathbf {A}}_j). \end{aligned}$$

Here we have used that $\sqrt{\gamma _{i,d,n}}=1-O(n^{-1})=\gamma _{i,d,n}$, and in the last line we used that $\sum _{i=1}^d{\mathbf {A}}_i=0$. This implies, using the same argument as in Eq. (6.10), that

6.11 $$\begin{aligned} T_{0,1} = \lim _{N\rightarrow \infty }\frac{N^2}{nd^2}\left( \frac{d}{n}\right) ^{\frac{1}{2}} \tilde{\mathbb {E}}_{\varvec{\alpha }}\left[ \sum _{i,j=1}^d\sqrt{\gamma _{i,d,n}\gamma _{j,d,n}}P_{i,j}^{(1,0)}\left( \varvec{\Gamma }^{-1}\right) P^{2,1}_{i,j}(\tilde{ \mathbf {A}})\right] =0. \end{aligned}$$

### Evaluating $T_{s,r}$ for $s+r=2$

For $s+r=2$, we first observe that

$$\begin{aligned}&\lim _{N\rightarrow \infty }\frac{N^2}{nd^2}\left( \frac{d}{n}\right) ^{\frac{s+r}{2}} \tilde{\mathbb {E}}_{\varvec{\alpha }}\left[ \sum _{i,j=1}^d\sqrt{\gamma _{i,d,n}\gamma _{j,d,n}}P_{i,j}^{(1,s)}\left( \varvec{\Gamma }^{-1}\right) P^{(2,r)}_{i,j}(\tilde{ \mathbf {A}})\right] \\&\quad = \lim _{N\rightarrow \infty }\frac{N^2}{n^2d}\tilde{\mathbb {E}}_{\varvec{\alpha }}\left[ \sum _{i,j=1}^d\sqrt{\gamma _{i,d,n}\gamma _{j,d,n}}P_{i,j}^{(1,s)}\left( \varvec{\Gamma }^{-1}\right) P^{(2,r)}_{i,j}(\tilde{ \mathbf {A}})\right] \\&\quad =\frac{1}{d}\lim _{N\rightarrow \infty }\tilde{\mathbb {E}}_{\varvec{\alpha }}\left[ \sum _{i,j=1}^d\sqrt{\gamma _{i,d,n}\gamma _{j,d,n}}P_{i,j}^{(1,s)}\left( \varvec{\Gamma }^{-1}\right) P^{(2,r)}_{i,j}(\tilde{ \mathbf {A}})\right] . \end{aligned}$$

Therefore we can replace all occurrences of $\gamma _{i,d,n}$ by 1 using the same argument as in Eq. (6.10). There are three cases to take care of, $(s,r)=\lbrace (2,0),(1,1),(0,2)\rbrace$. For $(s,r)=(2,0)$, we first look at the term

$$\begin{aligned} \sum _{i,j}\sum _{k,l:i\ne k\ne l\ne i} \varvec{\Gamma }_{ik}^{-1} \varvec{\Gamma }_{il}^{-1}&=d\sum _{i,k,l:i\ne k\ne l\ne i} \varvec{\Gamma }_{ik}^{-1}\varvec{\Gamma }_{il}^{-1}\\&=d\sum _{i,k,l:i\ne k\ne l\ne i}\frac{1}{\left( \pmb {\mathscr {A}}_i-\pmb {\mathscr {A}}_k\right) \left( \pmb {\mathscr {A}}_i-\pmb {\mathscr {A}}_l\right) }, \end{aligned}$$

where we have defined $\pmb {\mathscr {A}}_i=\mathbf {A}_i-i\sqrt{\frac{d}{n}}$. For fixed $i_0\ne k_0\ne j_0\ne i_0$, all permutations of these indices appear in the sum. For these terms with $i,j,k\in \{i_0,j_0,k_0\}$,

$$\begin{aligned}&\sum _{\begin{array}{c} i,j,k\in \{i_0,j_0,k_0\}\\ i\ne k\ne j\ne i \end{array}}\frac{1}{\left( \pmb {\mathscr {A}}_i-\pmb {\mathscr {A}}_j\right) \left( \pmb {\mathscr {A}}_i -\pmb {\mathscr {A}}_k\right) }\\&\quad = \frac{2}{\left( \pmb {\mathscr {A}}_{i_0}-\pmb {\mathscr {A}}_{j_0}\right) \left( \pmb {\mathscr {A}}_{j_0}-\pmb {\mathscr {A}}_{k_0}\right) \left( \pmb {\mathscr {A}}_{k_0} -\pmb {\mathscr {A}}_{i_0}\right) }\\&\qquad \left( -\left( \pmb {\mathscr {A}}_{j_0}-\pmb {\mathscr {A}}_{k_0}\right) -\left( \pmb {\mathscr {A}}_{k_0}- \pmb {\mathscr {A}}_{i_0}\right) -\left( \pmb {\mathscr {A}}_{i_0}-\pmb {\mathscr {A}}_{j_0}\right) \right) =0, \end{aligned}$$

implying

$$\begin{aligned} \sum _{i,j}\sum _{k,l:i\ne k\ne l\ne i} \varvec{\Gamma }_{ik}^{-1} \varvec{\Gamma }_{il}^{-1}&=0 \end{aligned}$$

and therefore

6.12 $$\begin{aligned} T_{2,0} = \lim _{N\rightarrow \infty }\frac{N^2}{n^2d}\tilde{\mathbb {E}}_{\varvec{\alpha }}\left[ \sum _{i,j=1}^d\sqrt{\gamma _{i,d,n}\gamma _{j,d,n}}P_{i,j}^{(1,2)}\left( \varvec{\Gamma }^{-1}\right) P^{(2,0)}_{i,j}(\tilde{ \mathbf {A}})\right] =0. \end{aligned}$$

Moving on to the case $(s,r)=(0,2)$, we first note that, as in the previous case $(s,r)=(2,0)$ and again replacing all occurrences of $\gamma _{i,d,n}$ by 1, we have

6.13 $$\begin{aligned}&\lim _{N\rightarrow \infty }\tilde{\mathbb {E}}[\sum _{i,j=1}^dP^{(2,2)}_{i,j}(\tilde{\mathbf {A}})]\nonumber \\&\quad =\sum _{i,j=1}^d\lim _{N\rightarrow \infty }\tilde{\mathbb {E}}[P^{(2,2)}_{i,j}(\mathbf {A})]\nonumber \\&\quad =\sum _{i,j=1}^d\mathbb {E}\left[ \frac{3}{8}({\mathbf {S}}_i^2+{\mathbf {S}}_j^2)+\frac{1}{4} {\mathbf {S}}_i{\mathbf {S}}_j\right] \nonumber \\&\quad =\frac{3d}{4}\mathbb {E}\left[ \sum _{i=1}^d{\mathbf {S}}_i^2\right] +\frac{1}{4} \mathbb {E}\left[ \sum _{i,j=1}^d{\mathbf {S}}_i{\mathbf {S}}_j\right] . \end{aligned}$$

Here, $\mathbf {S}=\mathrm {spec}(\mathbf {G})\sim {\text {GUE}}^0_d$ and we have used Lemma 6.1 to switch back to the unrestricted expectation value and Theorem 4.1 in the second equality. First we observe that $\mathbf {G}$ is traceless, and hence $\sum _{i=1}^d{\mathbf {S}}_i=0$ such that the second term in (6.13) vanishes. For the first term in (6.13), let $\mathbf {X}\sim {\text {GUE}}_d$ such that $\mathbf {G} = \mathbf {X} - \frac{{{\,\mathrm{tr}\,}}(\mathbf {X})}{d} I\sim {\text {GUE}}^0_d$. We calculate

6.14 $$\begin{aligned} \sum \nolimits _i \mathbf {S}_i^2&= {{\,\mathrm{tr}\,}}(\mathbf {G}^2)\nonumber \\&= {{\,\mathrm{tr}\,}}(\mathbf {X}^2) - \frac{1}{d} {{\,\mathrm{tr}\,}}(\mathbf {X})^2\nonumber \\&= \sum _{i,j} |\mathbf {X}_{ij}|^2 - \frac{1}{d}\left( \sum \nolimits _i \mathbf {X}_{ii}\right) ^2\nonumber \\&= \sum _{i} \mathbf {X}_{ii}^2 + \sum _{i\ne j} |\mathbf {X}_{ij}|^2 - \frac{1}{d}\sum _i \mathbf {X}_{ii}^2 - \frac{2}{d} \sum _{i\ne j} \mathbf {X}_{ii}\mathbf {X}_{jj}. \end{aligned}$$

We have $\mathbb {E}[\mathbf {X}_{ii}^2] = \mathbb {V}[\mathbf {X}_{ii}] = 1$, where $\mathbb {V}[\cdot ]$ denotes the variance of a random variable. Similarly, for $i\ne j$,

$$\begin{aligned} \mathbb {E}[|\mathbf {X}_{ij}|^2] = \mathbb {V}[\mathfrak {R}(\mathbf {X}_{ij})] + \mathbb {V}[\mathfrak {I}(\mathbf {X}_{ij})] = \frac{1}{2} + \frac{1}{2} = 1, \end{aligned}$$

and $\mathbb {E}[\mathbf {X}_{ii}\mathbf {X}_{jj}] = \mathbb {E}[\mathbf {X}_{ii}]\mathbb {E}[\mathbf {X}_{jj}] = 0$, since the entries of a ${\text {GUE}}_d$-matrix are independent. Hence, taking expectation values in (6.14) gives

$$\begin{aligned} \mathbb {E}_{\varvec{\alpha }}\left[ \sum \nolimits _i \mathbf {S}_i^2\right]&= d + d(d-1) - 1 = d^2-1, \end{aligned}$$

and we can calculate

6.15 $$\begin{aligned} T_{0,2} = \lim _{N\rightarrow \infty }\frac{N^2}{n^2d}\tilde{\mathbb {E}}_{\varvec{\alpha }}\left[ \sum _{i,j=1}^d\sqrt{\gamma _{i,d,n}\gamma _{j,d,n}}P_{i,j}^{(1,0)}\left( \varvec{\Gamma }^{-1}\right) P^{(2,2)}_{i,j}(\tilde{ \mathbf {A}})\right] =\frac{3}{4} \left( d^2-1\right) . \end{aligned}$$

We finally turn to the only missing case, $(s,r)=(1,1)$. The polynomial $P^{(1,1)}_{ij}$ is symmetric in *i* and *j*, therefore we can simplify

6.16 $$\begin{aligned}&\sum _{i,j}P^{(1,1)}_{ij}(\varvec{\Gamma }^{-1})P^{(2,1)}_{ij} (\varvec{\Gamma }^{-1})\nonumber \\&\quad =-\frac{1}{2}\sum _{i,j}\left( \sum _{k:k\ne i}\varvec{\Gamma }_{ik}^{-1}+\sum _{l:l\ne j} \varvec{\Gamma }_{jl}^{-1}\right) \left( \tilde{\mathbf {A}}_i+\tilde{\mathbf {A}}_j\right) \nonumber \\&\quad =-\sum _{i,j}\left( \sum _{k:k\ne i}\varvec{\Gamma }_{ik}^{-1}+\sum _{l:l\ne j} \varvec{\Gamma }_{jl}^{-1}\right) \tilde{\mathbf {A}}_i\nonumber \\&\quad =-\sum _{i,j}\left( \sum _{k:k\ne i}\varvec{\Gamma }_{ik}^{-1}+\sum _{l:l\ne j} \varvec{\Gamma }_{jl}^{-1}\right) {\mathbf {A}}_i\nonumber \\&=-d\sum _{i,k:k\ne i}\varvec{\Gamma }_{ik}^{-1}\mathbf {A}_i, \end{aligned}$$

where we have used in the second-to-last equation that we can replace any occurrence of $\gamma _{i,d,n}$ by one, and the last equation follows by the same reasoning as used in the case $(s,r)=(0,1)$ above. Now observe that for each $i\ne k$, both $\Gamma _{ik}^{-1}$ and $\Gamma _{ki}^{-1}=-\Gamma _{ik}^{-1}$ occur in the sum. Therefore we can simplify

6.17 $$\begin{aligned} \sum _{i,k:k\ne i}\varvec{\Gamma }_{ik}^{-1}\mathbf {A}_i&=\sum _{i,k: i<k}\varvec{\Gamma }_{ik}^{-1}(\mathbf {A}_i-\mathbf {A}_k)\nonumber \\&=\sum _{i,k: i<k}\varvec{\Gamma }_{ik}^{-1}\left( \mathbf {A}_i-\mathbf {A}_k+\frac{k-i}{\sqrt{\frac{n}{d}}}\right) +\sqrt{\frac{d}{n}}\sum _{i,k: i<k}(i-k)\varvec{\Gamma }_{ik}^{-1}\nonumber \\&=\sum _{i,k: i<k}\varvec{\Gamma }_{ik}^{-1}\varvec{\Gamma }_{ik}+\sqrt{\frac{d}{n}}\sum _{i,k: i<k}(i-k)\varvec{\Gamma }_{ik}^{-1}\nonumber \\&=\frac{d(d-1)}{2}-O\left( n^{-1/2}\right) \sum _{i,k: i<k}(i-j)\varvec{\Gamma }_{ik}^{-1}, \end{aligned}$$

where we have used the definition of $\varvec{\Gamma }_{ij}$ in the last equality. Combining Eqs. (6.16) and (6.17) we arrive at

6.18 $$\begin{aligned} T_{1,1} = \lim _{N\rightarrow \infty }\frac{N^2}{n^2d}\tilde{\mathbb {E}}_{\varvec{\alpha }}\left[ \sum _{i,j=1}^d\sqrt{\gamma _{i,d,n}\gamma _{j,d,n}}P_{i,j}^{(1,1)}\left( \varvec{\Gamma }^{-1}\right) P^{(2,1)}_{i,j}(\tilde{ \mathbf {A}})\right]&=-\frac{d(d-1)}{2}. \end{aligned}$$

Collecting all the terms $T_{r,s}$ for $r+s\le 2$ that we have calculated in Eqs. (6.9) to (6.12), (6.15) and (6.18), we arrive at

$$\begin{aligned} \lim _{N\rightarrow \infty }R_N&= \lim _{N\rightarrow \infty }\sum _{\begin{array}{c} r,s\in {0,1,2}\\ r+s\le 2 \end{array}}\frac{N^2}{nd^2}\left( \frac{d}{n}\right) ^{\frac{s+r}{2}} \tilde{\mathbb {E}}_{\varvec{\alpha }}\\&\quad \left[ \delta _{s0}\delta _{r0} \frac{d^2 n}{N}-\sum _{i,j=1}^d\sqrt{\gamma _{i,d,n}\gamma _{j,d,n}}P_{i,j}^{(1,s)}\left( \varvec{\Gamma }^{-1}\right) P^{(2,r)}_{i,j}(\tilde{ \mathbf {A}})\right] \\&= T_{0,0} + T_{0,1} + T_{1,0} + T_{0,2} + T_{2,0} + T_{1,1}\\&=\frac{d(d+1)}{2}-1-\frac{3(d^2-1)}{4}+\frac{d(d-1)}{2}\\&=\frac{d^2-1}{4}, \end{aligned}$$

which implies that

$$\begin{aligned} \lim _{N\rightarrow \infty }N\left( 1-F^{\mathrm {std}}_d(N)\right)&=\frac{d^2-1}{4}. \end{aligned}$$

To determine the lower order term, note that in all expressions above we have neglected terms of at most $O(n^{-1/2+\varepsilon (d-2)})$. Eq. (6.8) shows that the terms with $r+s\ge 3$ are $O(n^{-1/2+3/2\varepsilon })$, and the difference between $R_N$ and $N\left( 1-F^{\mathrm {std}}_d(N)\right)$ is $O(n^{-1/2+\varepsilon (d-2)})$ as well. As $\varepsilon \in (0,(d-2)^{-1})$ was arbitrary we conclude that, for all $\delta >0$,

$$\begin{aligned} F^{\mathrm {std}}_d(N)&=1-\frac{d^2-1}{4N}+O(N^{-3/2+\delta }), \end{aligned}$$

which concludes the proof. $\quad \square$

### Asymptotics of the optimal protocol

In this section, our goal is to obtain an asymptotic lower bound on the optimal entanglement fidelity $F_d^*$ of a deterministic PBT protocol with both the entangled resource state and the POVM optimized. This is achieved by restricting the optimization in Eq. (3.6) to the class of protocol families that use a density $c_\mu$ such that the probability distribution $q(\mu )=c_\mu p_{N,d}(\mu )$ converges for $N\rightarrow \infty$ in a certain sense. We then continue to show that the optimal asymptotic entanglement fidelity within this restricted class is related to the first eigenvalue of the Dirichlet Laplacian on the simplex of ordered probability distributions.

The main result of this section is the following theorem, which we restate from Sect. 1.2 for convenience.

#### Theorem 1.4

(Restated). The optimal fidelity for deterministic port-based teleportation is bounded from below by

$$\begin{aligned} F_d^*(N)&\ge 1-\frac{\lambda _1(\mathrm {OS}_{d-1})}{dN^2}-O(N^{-3}), \end{aligned}$$

where

$$\begin{aligned} \mathrm {OS}_{d-1}=\left\{ x\in \mathbb {R}^d\bigg |\sum \nolimits _i x_i=1, x_i\ge x_{i+1}, x_d\ge 0\right\} \end{aligned}$$

is the $(d-1)$-dimensional simplex of ordered probability distributions with *d* outcomes and $\lambda _1(\Omega )$ is the first eigenvalue of the Dirichlet Laplacian on a domain $\Omega$.

Before commencing the proof of Theorem 1.4, let us build some intuition for the fact that the fidelity formula Eq. (3.6) is related to a Laplacian. Reparametrizing Equation (3.6), we obtain the expression

$$\begin{aligned} F^{*}_d(N) =d^{-N-2}\max _{p}\sum _{\alpha \vdash _d N-1}\left( \sum _{\mu =\alpha +\square }\sqrt{p(\mu )}\right) ^2, \end{aligned}$$

where the maximization is taken over all probability distributions *p* on the set of Young diagrams with *N* boxes. Rearranging the sums yields

6.19 $$\begin{aligned} F^{*}_d(N) =d^{-N-2}\max _{p}\sum _{\mu ,\mu '}\sqrt{p(\mu )}\sqrt{p(\mu ')}(1+(d-1)\delta _{\mu \mu '})+\lbrace \text {boundary terms}\rbrace , \end{aligned}$$

where the sum is taken over all pairs $\mu ,\mu '\vdash _d N$ such that $\mu '$ can be obtained from $\mu$ by removing one box and adding one box, and the “boundary terms” subsume differences that arise when it is possible to remove a box from $\mu$ and add one back such that the result is not a Young diagram (this will be made more rigorous below). It is now instructive to equip the set of Young diagrams with a graph structure, where we draw an edge between $\mu$ and $\mu '\ne \mu$ precisely whenever the pair $\mu ,\mu '$ is part of the sum in (6.19). This graph is, in fact, the intersection of the root lattice of $\mathfrak {su}(d)$ with a certain simplex. Observing that $\sqrt{p}$ is an $L^2$-normalized function, we conclude that Eq. (6.19) is equal to the graph Laplacian on the Young diagram lattice we have defined, up to a constant. Normalizing the Young diagrams as done in Sect. 6.1, we see that the graphs for increasing *N* are finer and finer discretizations of the simplex of ordered probability distributions. We can thus expect that, when *p* is a sufficiently nice function, these graph Laplacians converge to the continuous Laplacian.

For the proof of Theorem 1.4 it will be convenient to switch back and forth between summation over a lattice and integration, which is the content of Lemma 6.2 below. Before stating the lemma, we make a few definitions. For a set $\Omega$ we define $d(x,\Omega )\,{:}{=}\, \inf _{y\in \Omega } \Vert x-y\Vert _2$, and for $\delta \ge 0$ we define

$$\begin{aligned} \partial _\delta \Omega \,{:}{=}\, \lbrace x\in \Omega :d(x,\partial \Omega )\le \delta \rbrace . \end{aligned}$$

Let $V_{0}^{d-1}=\{x\in \mathbb {R}^d|\sum _{i=1}^dx_i=0\}$ and $\mathbb {Z}^d_0=\mathbb {Z}^d\cap V_0^{d-1}$. For a vector subspace $V\subset \mathbb {R}^d$ and lattice $\Lambda \subset \mathbb {R}^d$, we denote by $v+V$ and $v+\Lambda$ the affine space and affine lattice with the origin shifted to $v\in \mathbb {R}^d$, respectively. We denote by $\lbrace e_i\rbrace _{i=1}^d$ the standard basis in $\mathbb {R}^d$. For $y\in e_1+\frac{1}{N} \mathbb {Z}^d_0$, define $U_N(y)\subset e_1+V_0^{d-1}$ by the condition

$$\begin{aligned} x\in U_N(y)\Leftrightarrow \forall y'\in e_1+\frac{1}{N} \mathbb {Z}^d_0, y'\ne y:\Vert x-y\Vert _2<\Vert x-y'\Vert _2. \end{aligned}$$

In other words, up to sets of measure zero we have tiled $e_1+V_0^{d-1}$ regularly into neighborhoods of lattice points. This also induces a decomposition $\mathrm {OS}_{d-1}\subset e_1+ V_0^{d-1}$ via intersection, $U_N^\mathrm {OS}(y)=U_N(y)\cap \mathrm {OS}_{d-1}$. We define the function $g_{N}:e_1 + V_0^{d-1} \rightarrow e_1 + \frac{1}{N} \mathbb {Z}_0^d$ via $g_N(x) = y$ where *y* is the unique lattice point such that $x\in U_N(y)$, if such a point exists. On the measure-zero set $\left( \bigcup _{y\in e_1 + \frac{1}{N} \mathbb {Z}_0^d}U_N(y)\right) ^c$, the function $g_N$ can be set to an arbitrary value.

#### Lemma 6.2

Let $f\in C^1(\mathrm {OS}_{d-1})\cap C(\mathbb {R}^d)$ be such that $f(x)= O(d(x,\partial \mathrm {OS}_{d-1})^p)$ for some $p\ge 1$, and $f\equiv 0$ on $\mathbb {R}^d{\setminus }\mathrm {OS}_{d-1}$. Then,

$$\begin{aligned} \mathrm {(i)}&\quad \left| \frac{1}{N^{d-1}} \sum _{y\in \mathrm {OS}_{d-1}\cap \frac{1}{N}\mathbb {Z}^d} f(y) - \int _{\mathrm {OS}_{d-1}} f(g_N(x)) \mathrm {d}x \right| \le O(N^{-p-2}); \\ \mathrm {(ii)}&\quad \left| \int _{\mathrm {OS}_{d-1}} f(g_N(x)) \mathrm {d}x - \int _{ \mathrm {OS}_{d-1}} f(x) \mathrm {d}x \right| \le O(N^{-1}). \end{aligned}$$

If furthermore $f\in C^2(\mathrm {OS}_{d-1})$, then

$$\begin{aligned} \mathrm {(iii)}&\quad \int _{\mathrm {OS}_{d-1}} f(g_N(x))(-\Delta f)(g_N(x)) \mathrm {d}x = \int _{\mathrm {OS}_{d-1}} f(x) (-\Delta f)(x) \mathrm {d}x + O(N^{-1}). \end{aligned}$$

#### Proof

Throughout the proof we set $\Lambda \,{:}{=}\, \mathrm {OS}_{d-1}\cap \frac{1}{N}\mathbb {Z}^d$. Observe first that the largest radius of the cell $U_N(y)$ around $y\in \frac{1}{N}\mathbb {Z}^d$ is equal to half the length $\frac{\sqrt{d}}{N}$ of a main diagonal in a *d*-dimensional hypercube of length $\frac{1}{N}$. Setting $c\,{:}{=}\, \frac{\sqrt{d}}{2}$, it follows that $g_N^{-1}(y)\subseteq \mathrm {OS}_{d-1}$ for all $y\in \Lambda$ with

6.20 $$\begin{aligned} d(y,\partial \mathrm {OS}_{d-1}) > \frac{c}{N}. \end{aligned}$$

Hence, we can write

$$\begin{aligned} \int _{ \mathrm {OS}_{d-1}} f(g_N(x)) \mathrm {d}x = \sum _{y\in \Lambda } \omega (y) f(y), \end{aligned}$$

where $\omega (y)$ assigns the weight $N^{-d+1}$ to all $y\in \Lambda$ satisfying (6.20), and $0\le \omega (y)\le N^{-d+1}$ for all $y\in \partial _{c/N}\mathrm {OS}_{d-1}$ to compensate for i) the fact that in this region $g_N$ maps some $x\in \mathrm {OS}_{d-1}$ to a lattice point outside of $\mathrm {OS}_{d-1}$, and ii) the fact that for some lattice points in $y\in \mathrm {OS}_{d-1}$, not all of the neighborhood of *y* is contained in $\mathrm {OS}_{d-1}$, i.e., $U_N(y){\setminus } \mathrm {OS}_{d-1}\ne \emptyset$.

We bound

$$\begin{aligned}&\left| \sum _{y\in \Lambda } N^{-d+1} f(y) - \int _{\mathrm {OS}_{d-1}} f(g_N(x)) \mathrm {d}x \right| \\&\quad = \left| \sum _{y\in \Lambda } (N^{-d+1}-\omega (y)) f(y)\right| \\&\quad \le \sum _{y\in \partial _{c/N}\mathrm {OS}_{d-1}} N^{-d+1} |f(y)|\\&\quad \le \sum _{y\in \partial _{c/N}\mathrm {OS}_{d-1}} N^{-d+1} \left( \frac{c}{N}\right) ^p\\&\quad \le \frac{c}{N} C_d N^{d-2}N^{-d+1} \left( \frac{c}{N}\right) ^p \\&\quad = O(N^{-p-2}), \end{aligned}$$

where in the second inequality we used the assumption $f\in O(d(x,\partial \mathrm {OS}_{d-1})^p)$, and in the third inequality we used that there are at most $\frac{c}{N} C_d N^{d-2}$ lattice points in $\partial _{c/N}\mathrm {OS}_{d-1}$ for some constant $C_d$ that only depends on *d*. This proves (i).

In order to prove (ii), we first develop $f(g_N(x))$ into a Taylor series around a point *x*:

$$\begin{aligned} f(g_N(x)) = f(x) + (g_N(x)-x)^T\nabla f(x) + O(N^{-1}) \end{aligned}$$

where we used the bound $\Vert g_N(x) - x\Vert _2\le \frac{c}{N}$ for some constant *c* for the remainder term in the Taylor series. Hence, we have

$$\begin{aligned} \left| \int _{\mathrm {OS}_{d-1}} f(g_N(x)) \mathrm {d}x - \int _{ \mathrm {OS}_{d-1}} f(x) \mathrm {d}x \right|&\le \int _{ \mathrm {OS}_{d-1} } \left| (g_N(x)-x)^T\nabla f(x) \right| \,\mathrm {d}x + O(N^{-1})\\&\le \int _{ \mathrm {OS}_{d-1} } \Vert g_N(x)-x\Vert _2 \Vert \nabla f(x)\Vert _2 \,\mathrm {d}x + O(N^{-1})\\&\le \frac{c}{N} K {{\,\mathrm{vol}\,}}(\mathrm {OS}_{d-1}) + O(N^{-1})\\&= O(N^{-1}), \end{aligned}$$

where the second inequality follows from the Cauchy-Schwarz inequality, and in the third inequality we used the fact that by assumption $\Vert \nabla f(x)\Vert _2$ is a continuous function on the compact domain $\mathrm {OS}_{d-1}$ and therefore bounded by a constant *K*, proving (ii).

Finally, we prove assertion (iii). We denote by $\partial _{ij}f \,{:}{=}\, (e_i-e_j)^T \nabla f$ the partial derivative of *f* in the direction $e_{ij}\,{:}{=}\, e_i-e_j$. We approximate $\partial _{ij} f(x)$ using a central difference $D_{ij}[f(x)]\,{:}{=}\, f(x+\tfrac{h}{2} e_{ij}) - f(x-\tfrac{h}{2} e_{ij})$, where $h>0$ is to be chosen later. To this end, consider the Taylor expansions

$$\begin{aligned} f(x+\tfrac{h}{2} e_{ij})&= f(x) + \frac{h}{2} e_{ij}^T \nabla f(x) + O(h^2)\\ f(x-\tfrac{h}{2} e_{ij})&= f(x) - \frac{h}{2} e_{ij}^T \nabla f(x) + O(h^2). \end{aligned}$$

Subtracting the second expansion from the first and rearranging gives

6.21 $$\begin{aligned} \partial _{ij} f(x)= \frac{1}{h} D_{ij}[f(x)] + O(h). \end{aligned}$$

It is easy to see that

$$\begin{aligned} \sum _{i,j=1}^d e_{ij} e_{ij}^T = 2d 1_{V_0^{d-1}}, \end{aligned}$$

and hence, for the Laplacian $\Delta = {{\,\mathrm{tr}\,}}(H(\cdot ))$ on $V_0^{d-1}$ with $H(\cdot )$ the Hessian matrix, we have

6.22 $$\begin{aligned} \Delta f(x)&= {{\,\mathrm{tr}\,}}( H(f)(x))\nonumber \\&= {{\,\mathrm{tr}\,}}\left( 1_{V_0^{d-1}}H(f)(x)\right) \nonumber \\&= \frac{1}{2d} \sum _{i,j=1}^{d} e_{ij}^T H(f)(x) e_{ij}\nonumber \\&= \frac{1}{2d} \sum _{i,j=1}^{d} \partial _{ij}^2 f(x). \end{aligned}$$

Similarly, denoting by $\langle \cdot ,\cdot \rangle _{V_0^{d-1}}$ the inner product on $V_0^{d-1}$, we have

6.23 $$\begin{aligned} \langle \nabla f(x) ,\nabla f(x) \rangle _{V_0^{d-1}}&= \frac{1}{2d} \sum _{i,j=1}^d \langle \nabla f(x) ,e_{ij} e_{ij}^T \nabla f(x) \rangle _{V_0^{d-1}}\nonumber \\&= \frac{1}{2d} \sum _{i,j=1}^d \left( e_{ij}^T \nabla f(x)\right) ^2\nonumber \\&= \frac{1}{2d} \sum _{i,j=1}^d (\partial _{ij}f(x))^2. \end{aligned}$$

We now calculate, abbreviating $\sum \nolimits _{y\in \Lambda }' = \sum _{y\in \Lambda } \omega (y)$:

6.24 $$\begin{aligned}&\int _{\mathrm {OS}_{d-1}} f(g_N(x))(-\Delta f)(g_N(x)) \mathrm {d}x \nonumber \\&\qquad = {\sum {'}}_{y\in \Lambda } f(y) (-\Delta f)(y)\nonumber \\&\qquad = - \frac{1}{2d} {\sum {'}}_{y\in \Lambda } \sum _{i,j=1}^{d} f(y) \partial _{ij}^2 f(y)\nonumber \\&\qquad = \frac{1}{2d} {\sum {'}}_{y\in \Lambda } \sum _{i,j=1}^d (\partial _{ij}f(y))^2 - \frac{1}{2d} {\sum {'}}_{y\in \Lambda } \sum _{i,j=1}^{d} \partial _{ij} \left[ f(y) \partial _{ij} f(y) \right] \nonumber \\&\qquad = {\sum {'}}_{y\in \Lambda } \langle \nabla f(y), \nabla f(y) \rangle _{V_0^{d-1}} - \frac{1}{2dh} \sum _{i,j=1}^{d} {\sum {'}}_{y\in \Lambda } D_{ij}[f(y)\partial _{ij} f(y)] + O(h), \end{aligned}$$

where we used (6.22) in the second equality, and (6.23) and (6.21) in the last equality.

For the first term in (6.24), we have

$$\begin{aligned} {\sum {'}}_{y\in \Lambda } \langle \nabla f(y), \nabla f(y) \rangle _{V_0^{d-1}}&= \int _{\mathrm {OS}_{d-1}} \langle \nabla f(g_N(x)), \nabla f(g_N(x)) \rangle _{V_0^{d-1}} \mathrm {d}x\\&= \int _{\mathrm {OS}_{d-1}} \langle \nabla f(x), \nabla f(x) \rangle _{V_0^{d-1}} \mathrm {d}x + O(N^{-1})\\&= \int _{\mathrm {OS}_{d-1}} f(x) (-\Delta f(x)) \mathrm {d}x + O(N^{-1}), \end{aligned}$$

where the second equality follows from (ii), and the third equality is ordinary integration by parts. For the second term in (6.24), we use the definition of $D_{ij}$ to obtain

6.25 $$\begin{aligned}&\frac{1}{2dh} \sum _{i,j=1}^d {\sum {'}}_{y\in \Lambda } D_{ij}[f(y)\partial _{ij} f(y)] + O(h) \nonumber \\&\quad = \frac{1}{2dh} \sum _{i,j=1}^d {\sum {'}}_{y\in \Lambda } \left( f(y+\tfrac{h}{2}e_{ij})\partial _{ij} f(y+\tfrac{h}{2}e_{ij}) - f(y-\tfrac{h}{2}e_{ij})\partial _{ij} f(y-\tfrac{h}{2}e_{ij}) \right) + O(h).\nonumber \\ \end{aligned}$$

We choose $h=O(N^{-1})$ such that $y\pm \tfrac{h}{2} e_{ij}\in \Lambda$ for all $y\in \Lambda$ sufficiently far away from the boundary of $\Lambda$. Then all terms in (6.25) cancel except for those terms involving evaluations of *f* on $\partial _{h}\mathrm {OS}_{d-1}$ or outside $\mathrm {OS}_{d-1}$. But these terms in turn are $O(h)=O(N^{-1})$, which can be seen using the same arguments as those in the proof of (ii). It follows that, with the above choice of $h=O(N^{-1})$,

$$\begin{aligned} \frac{1}{2dh} \sum _{i,j=1}^d {\sum {'}}_{y\in \Lambda } D_{ij}[f(y)\partial _{ij} f(y)] + O(h) = O(N^{-1}). \end{aligned}$$

In summary, we have shown that

$$\begin{aligned} \int _{\mathrm {OS}_{d-1}} f(g_N(x))(-\Delta f)(g_N(x)) \mathrm {d}x = \int _{\mathrm {OS}_{d-1}} f(x) (-\Delta f(x)) \mathrm {d}x + O(N^{-1}), \end{aligned}$$

which is what we set out to prove.

We are now ready to prove Theorem 1.4:

#### Proof of Thm. 1.4

Fix a dimension *d*, and let $a\in C^2(\mathrm {OS}_{d-1})$ be twice continuously differentiable^5^ such that $a|_{\partial \mathrm {OS}_{d-1}}\equiv 0$, $a(x)\ge 0$ for all $x\in \mathrm {OS}_{d-1}$, and $\Vert a\Vert _2=1$, where $\Vert \cdot \Vert _2$ is the $L_2$-norm on $\mathrm {OS}_{d-1}$. As *d* is fixed throughout the proof, we omit indicating any dependence on *d* except when we would like to emphasize the dimension of an object. Note that clearly $a\in L_2(\mathrm {OS}_{d-1})$ as *a* is continuous and $\mathrm {OS}_{d-1}$ is compact.

We use the square of a scaled version of *a* as a candidate probability distribution *q* on Young diagrams $\mu$ with *N* boxes and at most *d* rows,

6.26 $$\begin{aligned} q(\mu )=\frac{\eta _{N}}{N^{d-1}}a^2\left( \frac{\mu }{N}\right) . \end{aligned}$$

Here $\eta _{N}$ is a normalization constant which is close to one. Roughly speaking, this is due to the fact that the normalization condition for $q(\mu )$ is essentially proportional to a Riemann sum for the integral that calculates the $L_2$-norm of *a*, which is equal to unity by assumption. Indeed, since $a^2$ satisfies the assumptions of Lemma 6.2 with $p=1$, we have

$$\begin{aligned} 1&=\sum _{\mu \vdash _d N}q(\mu )\\&=\frac{\eta _{N}}{N^{d-1}}\sum _{\mu \in \mathbb {Z}^d\cap N\mathrm {OS}_{d-1}}a^2\left( \frac{\mu }{N}\right) \\&=\frac{\eta _{N}}{N^{d-1}}\sum _{y\in \left( \frac{1}{N} \mathbb {Z}^d\right) \cap \mathrm {OS}_{d-1}}a^2\left( y\right) \\&= \eta _N\left( \int _{ \mathrm {OS}_{d-1} } a^2(g_N(y)) \mathrm {d}y + O(N^{-3})\right) \\&= \eta _N\left( \int _{ \mathrm {OS}_{d-1} } a^2(y) \mathrm {d}y + O(N^{-1})\right) \\&= \eta _N \left( 1 + O(N^{-1}) \right) , \end{aligned}$$

where the fourth and fifth equality follow from Lemma 6.2(i) and (ii), respectively, and the last equality follows from $\Vert a\Vert _2=1$. Hence, $\eta _N = 1 + O(N^{-1})$.

Before we proceed, we restate the fidelity formula in (3.6) for the optimal deterministic protocol for the reader’s convenience:

6.27 $$\begin{aligned} F^{*}_d(N) =d^{-N-2}\max _{c_\mu }\sum _{\alpha \vdash _d N-1}\left( \sum _{\mu =\alpha +\square }\sqrt{c_\mu d_\mu m_{d,\mu }}\right) ^2. \end{aligned}$$

We bound this expression from below by choosing $c_\mu =q(\mu )/p(\mu )$, where $q(\mu )$ is defined as in (6.26) and $p(\mu )=\frac{d_\mu m_{d,\mu }}{d^N}$ is the Schur–Weyl distribution. The choice of $c_\mu$ in (6.27) corresponds to a particular PBT protocol whose entanglement fidelity we denote by $F_a$ in the following. It will be convenient to rewrite the sums over Young diagrams $\alpha \vdash _d N-1$ and $\mu =\alpha +\square$ in (6.27) as a sum over Young diagrams $\mu \vdash _d N$ and $i,j=1,\ldots , d$, requiring that both $\mu +e_i-e_j$ and $\mu - e_j$ be Young diagrams themselves. Using this trick, the quantity $\frac{d^2}{\eta _N} F_a$ can be expressed as

6.28 $$\begin{aligned} \frac{d^2}{\eta _N} F_a&=N^{-d+1} \sum _{\mu \vdash _{d}N}a\left( \frac{\mu }{N}\right) \sum _{i,j=1}^d\mathbb {1}_{\mathrm {YD}} (\mu +e_i-e_j) \mathbb {1}_{\mathrm {YD}}(\mu -e_j) a\left( \frac{\mu +e_i-e_j}{N}\right) \nonumber \\&= N^{-d+1} \sum _{\mu \vdash _{d}N}a\left( \frac{\mu }{N}\right) \sum _{i,j=1}^da\left( \frac{\mu +e_i-e_j}{N}\right) \nonumber \\&\qquad {} + N^{-d+1} \sum _{\mu \vdash _{d}N}a\left( \frac{\mu }{N}\right) \sum _{i,j=1}^d \mathbb {1}_{\mathrm {YD}}(\mu +e_i-e_j) \mathbb {1}_{\mathrm {YD}}(\mu -e_j) a\left( \frac{\mu +e_i-e_j}{N}\right) \nonumber \\&\qquad {} - N^{-d+1} \sum _{\mu \vdash _{d}N}a\left( \frac{\mu }{N}\right) \sum _{i,j=1}^da\left( \frac{\mu +e_i-e_j}{N}\right) . \end{aligned}$$

We first argue that up to order $N^{-2}$ we only need to consider the first term in the above expression. To this end, we rewrite the sum in the second term as an integral,

$$\begin{aligned}&N^{-d+1} \sum _{\mu \vdash _{d}N}a\left( \frac{\mu }{N}\right) \sum _{i,j=1}^d f_{i,j}(\mu )a\left( \frac{\mu +e_i-e_j}{N}\right) \\&\quad = \int _{ \mathrm {OS}_{d-1} } h_N(x) a(g_N(x))\sum _{i,j=1}^d f_{i,j}(x) a\left( g_N(x) + \frac{e_i-e_j}{N}\right) \mathrm {d}x, \end{aligned}$$

where $f_{i,j}(x)\,{:}{=}\, \mathbb {1}_{\mathrm {YD}}(Ng_N(x)+e_i-e_j) \mathbb {1}_{\mathrm {YD}}(Ng_N(x)-e_j)$. The function $h_N(x)\in [0,1]$ takes care of normalization around the boundaries of $\mathrm {OS}_{d-1}$, that is, $h_N(x)=1$ except in a region $\partial _{c_1/N}\mathrm {OS}_{d-1}$ for some constant $c_1$ that only depends on *d*. Note that the same statement is true for the function $f_{i,j}(x)$, and therefore, this also holds for the product $h_N(x)f_{i,j}(x)$. Using Lemma 6.2(i) for the third term in (6.28) gives

$$\begin{aligned}&N^{-d+1} \sum _{\mu \vdash _{d}N}a\left( \frac{\mu }{N}\right) \sum _{i,j=1}^da\left( \frac{\mu +e_i-e_j}{N}\right) \\&\quad = \int _{\mathrm {OS}_{d-1}} a(g_N(x))\sum _{i,j=1}^d a\left( g_N(x) + \frac{e_i-e_j}{N} \right) \mathrm {d}x + O(N^{-3}). \end{aligned}$$

Hence, for the difference of the second and third term in (6.28), we obtain

$$\begin{aligned}&N^{-d+1} \sum _{\mu \vdash _{d}N}a\left( \frac{\mu }{N}\right) \sum _{i,j=1}^d \left[ \mathbb {1}_{\mathrm {YD}}(\mu +e_i-e_j) \mathbb {1}_{\mathrm {YD}}(\mu -e_j) -1 \right] a\left( \frac{\mu +e_i-e_j}{N}\right) \\&\qquad {} = \int _{\mathrm {OS}_{d-1}}a(g_N(x))\sum _{i,j=1}^d\left[ h_N(x)f_{i,j}(x)-1\right] a\left( g_N(x)+\frac{e_i-e_j}{N}\right) \mathrm {d}x + O(N^{-3})\\&\qquad {} \le \frac{c_2}{N^2}\int _{\partial _{c_1/N}\mathrm {OS}_{d-1}}\left[ h_N(x)\mathbb {1}_{\mathrm {YD}}(Ng_N(x)+e_i-e_j) \mathbb {1}_{\mathrm {YD}}(Ng_N(x)-e_j) -1\right] \mathrm {d}x + O(N^{-3})\\&\qquad {} \le \frac{c_3}{N^2}{{\,\mathrm{vol}\,}}(\partial _{c_1/N}\mathrm {OS}_{d-1}) + O(N^{-3})\\&\qquad {} = O(N^{-3}) \end{aligned}$$

for some constants $c_2$ and $c_3$. Here, the first inequality is obtained by a Taylor expansion of the different occurrences of *a* around the respective closest boundary point and using the fact that *a* vanishes on the boundary by assumption. The second inequality follows since $h_N$ is bounded uniformly in *N*.^6^

We now turn to the first term in (6.28), applying Lemma 6.2(i) once more to obtain

$$\begin{aligned}&N^{-d+1} \sum _{\mu \vdash _{d}N}a\left( \frac{\mu }{N}\right) \sum _{i,j=1}^da\left( \frac{\mu +e_i-e_j}{N}\right) \\&\quad =\int _{ \mathrm {OS}_{d-1} } a(g_N(x)) \sum _{i,j=1}^d a\left( g_N(x)+ \frac{e_i-e_j}{N}\right) \mathrm {d}x + O(N^{-3}). \end{aligned}$$

Expanding $a\left( g_N(x)+\frac{e_i-e_j}{N}\right)$ into a Taylor series gives

$$\begin{aligned} a\left( g_N(x)+\frac{e_i-e_j}{N}\right)&=a(g_N(x))+\frac{1}{N} \langle e_i-e_j, (\nabla a)(g_N(x))\rangle _{V_0^{d-1}}\\&\quad +\frac{1}{2N^2}{{\,\mathrm{tr}\,}}\left[ (e_i-e_j)(e_i-e_j)^T (H(a))(g_N(x))\right] +O(N^{-3}), \end{aligned}$$

where $\langle \cdot ,\cdot \rangle _{V_0^{d-1}}$ is the standard inner product on $V_0^{d-1}$ and *H*(*a*) denotes the Hessian of *a* on $V_0^{d-1}$. Summing over *i* and *j* yields

6.29 $$\begin{aligned} \sum _{i,j=1}^d e_i-e_j&=0&\sum _{i,j=1}^d(e_i-e_j)(e_i-e_j)^T&=2d 1_{V_0^{d-1}}. \end{aligned}$$

It follows that

$$\begin{aligned}&\int _{\mathrm {OS}_{d-1}}a(g_N(x))\sum _{i,j=1}^da\left( g_N(x)+\frac{e_i-e_j}{N}\right) \mathrm {d}x +O(N^{-3})\\&\qquad {} =\int _{\mathrm {OS}_{d-1}}a(g_N(x))\left( d^2a(g_N(x))+\frac{d}{N^2}(\Delta a)(g_N(x))\right) \mathrm {d}x+O(N^{-3})\\&\qquad {} = \frac{d^2}{N^{d-1}} \sum _{y\in \mathrm {OS}_{d-1}\cap \frac{1}{N}\mathbb {Z}^d} a^2(y) -\frac{d}{N^2}\int _{\mathrm {OS}_{d-1}}a(g_N(x))(-\Delta a)(g_N(x))\mathrm {d}x+O(N^{-3})\\&\qquad {} =\frac{d^2}{\eta _{N}}-\frac{d}{N^2}\int _{\mathrm {OS}_{d-1}}a(g_N(x))(-\Delta a)(g_N(x))\mathrm {d}x+O(N^{-3})\\&\qquad {} =\frac{d^2}{\eta _{N}}-\frac{d}{N^2}\int _{\mathrm {OS}_{d-1}}a(x)(-\Delta a)(x)\mathrm {d}x+O(N^{-3}), \end{aligned}$$

where in the first equality the $N{-1}$ term vanishes due to (6.29), and we defined the Laplace operator $\Delta (a)={{\,\mathrm{tr}\,}}H(a)$ on $V_0^{d-1}$. In the second equality we used Lemma 6.2(i) to switch back to discrete summation, in the third equality we used the normalization of *a*, and in the fourth equality we used Lemma 6.2(iii).

Putting together everything we have derived so far, we obtain

$$\begin{aligned} F_a=1-\frac{1}{dN^2}\int _{\mathrm {OS}_{d-1}}a(x)(-\Delta a)(x)\mathrm {d}x+O(N^{-3}). \end{aligned}$$

In equation Eq. (3.6), the fidelity is maximized over all densities $c_\mu$. The above expression shows, that restricting to the set of densities $c_\mu$ that stem from a function *a* on $\mathrm {OS}_{d-1}$ makes the problem equivalent to minimizing the expression

$$\begin{aligned} \int _{\mathrm {OS}_{d-1}}a(x)(-\Delta a)(x)\mathrm {d}x. \end{aligned}$$

When taking the infimum over $a\in H^2(\mathrm {OS}_{d-1})$, where $H^2(\mathrm {OS}_{d-1})$ is the Sobolev space of twice weakly differentiable functions, instead of $a\in C^2(\mathrm {OS}_{d-1})$, this is exactly one of the variational characterizations of the first Dirichlet eigenvalue of the Laplace operator on $\mathrm {OS}_{d-1}$. This is because the eigenfunction corresponding to the first eigenvalue of the Dirichlet Laplacian can be chosen positive (see, e.g., [62]). But $C^2(\mathrm {OS}_{d-1})$ is dense in $H^2(\mathrm {OS}_{d-1})$, which implies that

$$\begin{aligned} \sup _a F_a&=1-\frac{\lambda _1(\mathrm {OS}_{d-1})}{dN^2}+O(N^{-3}), \end{aligned}$$

where the supremum is taken over all non-negative functions $a\in C^2(\mathrm {OS}_{d-1})$. $\square$

Upper and lower bounds for the first Dirichlet eigenvalue of the Laplacian on a sufficiently well-behaved domain readily exist.

#### Theorem 6.3

[37, 63] For the first Dirichlet eigenvalue $\lambda _1(\Omega )$ on a bounded convex domain $\Omega \subset \mathbb {R}^d$, the following inequalities hold,

$$\begin{aligned} \lambda _1(\Omega )&\ge \lambda _1(B_1)\left( \frac{{{\,\mathrm{vol}\,}}(B_1)}{{{\,\mathrm{vol}\,}}(\Omega )}\right) ^\frac{2}{d}\text {, and}\\ \lambda _1(\Omega )&\le \lambda _1(B_1)\frac{{{\,\mathrm{vol}\,}}(\partial \Omega )}{dr_\Omega {{\,\mathrm{vol}\,}}(\Omega )}, \end{aligned}$$

where $B_1\subset \mathbb {R}^d$ is the unit ball and $r_\Omega$ is the inradius of $\Omega$.

The inradius of $\mathrm {OS}_{d-1}$ is equal to $1/d^2$. This can be seen by guessing the center of the inball $\hat{x}=((2d-1)/d^2,(2d-3)/d^2,\ldots ,1/d^2)$ and checking that the distance to each facet is $1/d^2$. Therefore we get the following lower bound on the optimal PBT fidelity. This theorem is stated in Sect. 1.2, and restated here for convenience.

#### Theorem 1.5

(Restated). For the optimal fidelity of port-based teleportation with arbitrary but fixed input dimension *d* and *N* ports, the following bound holds,

$$\begin{aligned} F^*_d(N)\ge 1-\frac{ d^5+O(d^{9/2})}{4\sqrt{2} N^2}+O(N^{-3}). \end{aligned}$$

#### Proof

Theorem 1.4 gives us the bound

$$\begin{aligned} F^*_d(N)\ge 1-\frac{\lambda _1(\mathrm {OS}_{d-1})}{dN^2}+O(N^{-3}). \end{aligned}$$

Using Theorem 6.3 and Lemma D.5 we bound

$$\begin{aligned} \lambda _1(\mathrm {OS}_{d-1})&\le \lambda _1(B_1^{d-1})\frac{{{\,\mathrm{vol}\,}}(\partial \Omega )}{dr_\Omega {{\,\mathrm{vol}\,}}(\Omega )}\\&\le \lambda _1(B_1^{d-1})d^2\left( \frac{d(d-1)}{\sqrt{2}}+\sqrt{d(d-1)}+\sqrt{2}\right) . \end{aligned}$$

The first eigenvalue of the Dirichlet Laplacian on the $(d-1)$-dimensional Ball is given by

$$\begin{aligned} \lambda _1(B_1^{d-1})=j^2_{\frac{d-3}{2},1}, \end{aligned}$$

where $j_{\nu ,l}$ is the *l*th root of the Bessel function of the first kind with parameter $\nu$. This is, in turn, bounded as [64]

$$\begin{aligned} j_{\nu ,1}\le \sqrt{\nu +1}(\sqrt{\nu +2}+1). \end{aligned}$$

Putting the inequalities together we arrive at

$$\begin{aligned} \lambda _1(B_1^{d-1})&\le \frac{d-1}{2}\left( \sqrt{\frac{d+1}{2}}+1\right) ^2,\\ \lambda _1(\mathrm {OS}_{d-1})&\le \frac{d-1}{2}\left( \sqrt{\frac{d+1}{2}}+1\right) ^2d^2\left( \frac{d(d-1)}{\sqrt{2}}+\sqrt{d(d-1)}+\sqrt{2}\right) , \text { and hence}\\ F^*_d(N)&\ge 1-\frac{ \frac{d-1}{2}\left( \sqrt{\frac{d+1}{2}}+1\right) ^2d\left( \frac{d(d-1)}{\sqrt{2}}+\sqrt{d(d-1)}+\sqrt{2}\right) }{N^2}+O(N^{-3})\\&=1-\frac{ d^5+O(d^{9/2})}{4\sqrt{2} N^2}+O(N^{-3}). \end{aligned}$$

In the appendix, we provide a concrete protocol in Theorem B.1 that achieves the same asymptotic dependence on *N* and *d*, with a slightly worse constant.

Intuitively it seems unlikely that a “wrinkly” distribution, i.e. a distribution that does not converge against an $L_1$ density on $\mathrm {OS}$, is the optimizer in Eq. (3.6). Supposing that the optimizer comes from a function *a* as described above, we can also derive a converse bound for the asymptotics of the entanglement fidelity $F^*_d(N)$ using Theorem 6.3.

#### Remark 6.4

Let $P^N_a$ be the PBT protocol with $c_\mu =N^{d-1}a^2(\mu /N)/P(\mu )$ for some function $a\in L_2(\mathrm {OS}_{d-1})$. For the asymptotic fidelity of such protocols for large *N* the following converse bound holds,

$$\begin{aligned} F_{a}(N)\le 1-\frac{\pi d^4+O(d^{3})}{8e^3N^2}+O(N^{-3}). \end{aligned}$$

This can be seen as follows. From Theorem 1.4 we have that

$$\begin{aligned} F_a\le 1-\frac{\lambda _1(\mathrm {OS}_{d-1})}{dN^2}+O(N^{-3}). \end{aligned}$$

Theorem 6.3 together with Lemma D.5 yields

$$\begin{aligned} \lambda _1(\mathrm {OS}_{d-1})&\ge \lambda _1(B_1)\left( \frac{{{\,\mathrm{vol}\,}}(B_1^{d-1})}{{{\,\mathrm{vol}\,}}(\mathrm {OS}_{d-1})}\right) ^\frac{2}{d}\\&=\lambda _1(B_1)\left( \frac{\pi ^{\frac{d-1}{2}}\sqrt{d}((d-1)!)^2}{\Gamma (\frac{d-1}{2}+1)}\right) ^\frac{2}{d}\\&\ge \pi ^{1-1/d}\lambda _1(B_1)\left( \frac{((d-1)!)^2}{\Gamma (\frac{d-1}{2}+1)}\right) ^\frac{2}{d} \end{aligned}$$

where in the second line we have used the volume of the $(d-1)$-dimensional Ball,

$$\begin{aligned} {{\,\mathrm{vol}\,}}(B_1^{d-1})=\frac{\pi ^{\frac{d-1}{2}}}{\Gamma (\frac{d-1}{2}+1)}, \end{aligned}$$

and $\Gamma (x)$ is the gamma function. Using bound versions of Stirling’s approximation we obtain

$$\begin{aligned} \lambda _1(\mathrm {OS}_{d-1})&\ge O(1)\lambda _1(B_1)\left( \frac{d-1}{e}\right) ^{3(1-1/d)}. \end{aligned}$$

Using a lower bound for the first zero of the Bessel function of the first kind [65] we bound

$$\begin{aligned} \lambda _1(B_1^{d-1})&\ge \left( \frac{d}{2}+c\right) ^2 \end{aligned}$$

for some constant *c*, so we finally arrive at

$$\begin{aligned} F_a&=1-\frac{\pi d^4+O(d^{3})}{8e^3N^2}+O(N^{-3}). \end{aligned}$$

This bound has the nice property that $N\propto d^2$ if the error of the PBT protocol is fixed, which is what we expect from information theoretic insights (see Sect. 7).

## Converse Bound

We begin by deriving a lower bound on the communication requirements for approximate quantum teleportation of any kind, i.e., not only for PBT. Such a result could be called folklore, but has, to the best of our knowledge, not appeared elsewhere.^7^

For the proof we need the converse bound for one-shot quantum state splitting that was given in [67] in terms of the smooth max-mutual information $I_{\max }^{\varepsilon }(E:A)_{\rho }$. To define this quantity, let $D_{\max }(\rho \Vert \sigma )=\min \left\{ \lambda \in \mathbb {R}\big |2^\lambda \sigma \ge \rho \right\}$ be the max-relative entropy [68], and let $P(\rho ,\sigma )\,{:}{=}\, \sqrt{1-F(\rho ,\sigma )}$ be the purified distance. Furthermore, let $B_\varepsilon (\rho )\,{:}{=}\, \lbrace \bar{ \rho }:\bar{ \rho }\ge 0, {{\,\mathrm{tr}\,}}\bar{ \rho }\le 1, P(\rho ,\bar{ \rho })\le \varepsilon \rbrace$ be the $\varepsilon$-ball of subnormalized states around $\rho$ with respect to the purified distance. The smooth max-mutual information is defined as

$$\begin{aligned} I_{\max }^{\varepsilon }(E:A)_{\rho }&\,{:}{=}\, \min _{\bar{\rho }\in B_\varepsilon (\rho )}I_{\max }(E:A)_{\bar{\rho }}, \end{aligned}$$

where $I_{\max }(E:A)_{\bar{\rho }}\,{:}{=}\,\min _{\sigma _A} D_{\max }(\bar{\rho }_{AE}\Vert \sigma _A\otimes \bar{\rho }_E)$ with the minimization over normalized quantum states $\sigma _A$.

### Lemma 7.1

Let

$$\begin{aligned} |\phi ^+\rangle _{AB}=\frac{1}{\sqrt{d}}\sum _{i=0}^{d-1}|ii\rangle _{AB}\in \mathcal {H}_A\otimes \mathcal {H}_B \end{aligned}$$

be the $d\times d$-dimensional maximally entangled state. Then

$$\begin{aligned} 2\log \left\lceil d(1-\varepsilon ^2)\right\rceil \ge I_{\max }^{\varepsilon }(A:B)_{\phi ^+}\ge 2\log \left( d(1-\varepsilon ^2)\right) . \end{aligned}$$

### Proof

Let $\rho \in B(\mathcal {H}_A\otimes \mathcal {H}_{B})$ be a quantum state such that $I_{\max }^{\varepsilon }(A:B)_{\phi ^+}=I_{\max }(A:B)_{\rho }$, and let $|\gamma \rangle _{ABE}$ be a purification of $\rho$. Uhlmann’s Theorem ensures that there exists a pure quantum state $|\alpha \rangle _E$ such that

7.1 $$\begin{aligned} \sqrt{1-\varepsilon ^2}\le \sqrt{F}(\phi ^+,\rho )=\langle \phi ^+|_{AB}\langle \alpha |_E|\gamma \rangle _{ABE}. \end{aligned}$$

This holds without taking the absolute value because any phase can be included in $|\alpha \rangle$. Let

7.2 $$\begin{aligned} |\gamma \rangle _{ABE}=\sum _{i=0}^{d-1}\sqrt{p_i}|\phi _i\rangle _{A} \otimes |\psi _i\rangle _{BE} \end{aligned}$$

be the Schmidt decomposition of $|\gamma \rangle$ with respect to the bipartition *A* : *BE*. Let further $U_A$ be the unitary matrix such that $U_A|i\rangle _A=|\phi _i\rangle _{A}$. Using the Mirror Lemma D.1 we get

$$\begin{aligned} |\phi ^+\rangle _{AB}&=U_AU_A^\dagger |\phi ^+\rangle _{AB}\\&=U_A\bar{U}_B|\phi ^+\rangle _{AB}\\&=\frac{1}{\sqrt{d}}\sum _{i=0}^{d-1}|\phi _i\rangle _{A}|\xi _i\rangle _{B}, \end{aligned}$$

where $\bar{U}$ is the complex conjugate in the computational basis and $|\xi _i\rangle _{B}=\bar{U}_B|i\rangle _B$. With this we obtain from (7.1) that

$$\begin{aligned} 1-\varepsilon ^2&\le (\langle \phi ^+|_{AB}\langle \alpha |_E|\gamma \rangle _{ABE})^2\\&= (\mathfrak {R}\langle \phi ^+|_{AB}\langle \alpha |_E|\gamma \rangle _{ABE} )^2\\&=\left( \sum _{i=0}^{d-1}\sqrt{\frac{p_i}{d}}\mathfrak {R}\langle \xi _i|_B\langle \alpha |_E|\psi _i\rangle _{BE}\right) ^2\\&\le \frac{1}{d}\sum _{i=0}^{d-1}\left( \mathfrak {R}\langle \xi _i|_B\langle \alpha |_E|\psi _i\rangle _{BE}\right) ^2\\&\le \frac{1}{d}\sum _{i=0}^{d-1}\mathfrak {R}\langle \xi _i|_B\langle \alpha |_E|\psi _i\rangle _{BE}. \end{aligned}$$

The second inequality is the Cauchy-Schwarz inequality and the third inequality follows from $\mathfrak {R}\langle \xi _i|_B\langle \alpha |_E|\psi _i\rangle _{BE}\le 1$.

The next step is to bound the max-mutual information of $\rho$. Let

$$\begin{aligned} \lambda =I_{\max }(A:B)_\rho =I_{\max }^{\varepsilon }(A:B)_{\phi ^+}. \end{aligned}$$

By the definition of $I_{\max }$ there exists a quantum state $\sigma _B$ such that

$$\begin{aligned} 2^\lambda =\left\| \rho _A^{-{\frac{1}{2}}}\otimes \sigma _B^{-{\frac{1}{2}}}\rho _{AB} \, \rho _A^{-{\frac{1}{2}}}\otimes \sigma _B^{-{\frac{1}{2}}}\right\| _\infty . \end{aligned}$$

Here, $X^{-1}$ denotes the pseudo-inverse of a matrix *X*, i.e., $X^{-1}X=XX^{-1}$ is equal to the projector onto the support of *X*. Let $|\phi _\sigma \rangle =\sqrt{d}\sigma _B^{1/2}|\phi ^+\rangle$ be the standard purification of $\sigma$. We bound

$$\begin{aligned} 2^\lambda&=\left\| \rho _A^{-{\frac{1}{2}}}\otimes \sigma _B^{-{\frac{1}{2}}}\rho _{AB} \, \rho _A^{-{\frac{1}{2}}}\otimes \sigma _B^{-{\frac{1}{2}}}\right\| _\infty \\&\ge \langle \phi _\sigma |\rho _A^{-{\frac{1}{2}}}\otimes \sigma _B^{-{\frac{1}{2}}}\rho _{AB} \, \rho _A^{-{\frac{1}{2}}}\otimes \sigma _B^{-{\frac{1}{2}}}|\phi _\sigma \rangle \\&={{\,\mathrm{tr}\,}}\langle \phi _\sigma |\rho _A^{-{\frac{1}{2}}}\otimes \sigma _B^{-{\frac{1}{2}}}|\gamma \rangle \langle \gamma |_{ABE}\rho _A^{-{\frac{1}{2}}}\otimes \sigma _B^{-{\frac{1}{2}}}|\phi _\sigma \rangle \\&\ge \langle \phi _\sigma |_{AB}\langle \alpha |_E\rho _A^{-{\frac{1}{2}}}\otimes \sigma _B^{-{\frac{1}{2}}}|\gamma \rangle \langle \gamma |_{ABE}\rho _A^{-{\frac{1}{2}}}\otimes \sigma _B^{-{\frac{1}{2}}}|\phi _\sigma \rangle _{AB}|\alpha \rangle _E\\&=d\left| \langle \phi ^+|_{AB}\rho _A^{-{\frac{1}{2}}}\langle \alpha |_E|\gamma \rangle _{ABE}\right| ^2\\&=\left| \sum \nolimits _i\langle \xi _i|_B\langle \alpha |_E|\psi _i\rangle _{BE}\right| ^2\\&\ge \left( \sum \nolimits _i\mathfrak {R}\langle \xi _i|_B\langle \alpha |_E|\psi _i\rangle _{BE}\right) ^2\\&\ge d^2(1-\varepsilon ^2)^2, \end{aligned}$$

where we used the particular form of $|\phi _\sigma \rangle$ in the third equality, and (7.2) in the fourth equality, together with the fact that $\lbrace p_i\rbrace _i$ are the eigenvalues of $\rho _A$. This proves the claimed up upper bound on $I_{\max }^{\varepsilon }(A:B)_{\phi ^+}$.

In order to prove the lower bound, let ${r}=\lceil d(1-\varepsilon ^2)\rceil$ and

$$\begin{aligned} |\phi ^+_{r}\rangle =\frac{1}{\sqrt{{r}}}\sum _{i=0}^{{r}-1}|ii\rangle _{AB}\in \mathcal {H}_A\otimes \mathcal {H}_B. \end{aligned}$$

Then we have

$$\begin{aligned} {I_{\max }(A:B)_{\phi ^+_{r}}}&= 2\log {r}=2\log \lceil d(1-\varepsilon ^2)\rceil \\ |\langle \phi ^+|\phi ^+_{r}\rangle |^2&= {r}/d\ge 1-\varepsilon ^2. \end{aligned}$$

The observation that $|\phi ^+_{r}\rangle \langle \phi ^+_{r}|$ is a point in the minimization over $\sigma$ finishes the proof.

$$\square $$

Using the special case of state merging/splitting with trivial side information and the converse bound from [67], we can bound the necessary quantum communication for simulating the identity channel with a given entanglement fidelity.

### Corollary 7.2

Let $\mathcal {E}_{AA'\rightarrow B}$, $\mathcal {D}_{BB'\rightarrow A}$ be quantum (encoding and decoding) channels with $\dim \mathcal {H}_A=d$ and $\dim \mathcal {H}_B= {d'}$ such that there exists a resource state $\rho _{A'B'}$ achieving

$$\begin{aligned} F(\mathcal {D}\circ \mathcal {E}((\cdot )\otimes \rho _{A'B'}))=1-\varepsilon ^2. \end{aligned}$$

Then the following inequality holds:

$$\begin{aligned} {d'}\ge d\left( 1-\varepsilon ^2\right) . \end{aligned}$$

### Proof

Using Lemma 7.1, this follows from applying the lower bound on the communication cost of one-shot state splitting from [67] to the special case where Alice and the reference system share a maximally entangled state. $\quad \square$

Together with superdense coding this implies a lower bound on approximate teleportation.

### Corollary 7.3

If in the above corollary $\mathcal {E}$ is a *qc*-channel, then

$$\begin{aligned} {d'}\ge d^2\left( 1-\varepsilon ^2\right) ^2. \end{aligned}$$

### Proof

This follows as any protocol with a lower classical communication in conjunction with superdense coding would violate Corollary 7.2. $\quad \square$

For the special case of port-based teleportation, this implies a lower bound on the number of ports.

### Corollary 7.4

Any port-based teleportation protocol with input dimension *d* and *N* ports has entanglement fidelity at most

$$\begin{aligned} F^*_d(N) \le \frac{\sqrt{N}}{d}. \end{aligned}$$

### Proof

In port-based teleportation, the only information that is useful to the receiver is which port to select. More precisely, given a protocol *P* for PBT in which Alice sends a message that is not a port number, we can construct a modified protocol *P* where Alice applies the procedure that Bob uses in *P* to deduce the port to select and then sends the port number instead. For a given entanglement fidelity *F*, having fewer than $\left( {d}{F}\right) ^2$ ports would therefore violate the bound from Corollary 7.3. $\quad \square$

The converse bound on the amount of quantum communication in Corollary 7.2 holds for arbitrary protocols implementing a simulation of the identity channels, and Corollary 7.3 puts a lower bound on the classical communication of any (approximate) teleportation scheme. We continue to derive a converse bound specifically for port-based teleportation that is nontrivial for all combinations of *d* and *N*. Let us consider a general port-based teleportation scheme, given by POVMs $\{E_{A^N}^{(i)}\}$ and a resource state $\rho _{A^NB^N}$, where $A_0\cong \mathbb {C}^d$ and $B_1,\ldots ,B_N\cong \mathbb {C}^d$. We would like to upper-bound the entanglement fidelity

7.3 $$\begin{aligned} F^*_d(N)&= F\left( \sum _{i=1}^N (I_{B_0} \otimes I_{B_i\rightarrow B_1}) {{\,\mathrm{tr}\,}}_{(B_0B_i)^c}[ (E_A^{(i)} \otimes I_B) (\rho _{A^NB^N} \otimes \phi ^+_{A_0B_0}) ]\right. \nonumber \\&\qquad \left. (I_{B_0} \otimes I_{B_i\rightarrow B_1}^\dagger ), \phi ^+_{B_0B_1}\right) , \end{aligned}$$

where $B_0\cong \mathbb {C}^d$ and $F(\rho ,\sigma )=\Vert \sqrt{\rho }\sqrt{\sigma }\Vert _1^2$ is the fidelity. This fidelity corresponds to the special case of Alice using an arbitrary PBT protocol to teleport half of a maximally-entangled state to Bob, who already possesses the other half. An upper bound for this fidelity then directly implies an upper bound for the entanglement fidelity of the PBT protocol. We prove the following

### Theorem 7.5

For any port-based teleportation scheme, the entanglement fidelity (7.3) can be bounded from above as

7.4 $$\begin{aligned} F^*_d(N)&\le 1-\frac{d^2-1}{8N^2}\frac{1}{1+\frac{d^2-2}{2N}}. \end{aligned}$$

Asymptotically, this bound becomes

7.5 $$\begin{aligned} F^*_d(N)&\le 1 - \frac{d^2-1}{8} \frac{1}{N^2} + O(N^{-3}). \end{aligned}$$

### Proof

Note first that for a pure state $|\psi \rangle$ we have $F(\psi ,\tau ) = \langle \psi |\tau |\psi \rangle$ for any mixed state $\tau$, and hence $\tau \mapsto F(\psi ,\tau )$ is linear for any $\tau$. Since $\phi ^+_{B_0B_1}$ is pure, the entanglement fidelity (7.3) can hence be rewritten as

$$\begin{aligned} F^*_d(N)&= \sum _{i=1}^N p(i) F\left( \frac{1}{p(i)}{{\,\mathrm{tr}\,}}_{(B_0B_i)^c}[((E^{(i)})^{1/2}_A \otimes I_B) (\rho _{A^NB^N} \otimes \phi ^+_{A_0B_0})\right. \\&\qquad \left. ((E^{(i)})^{1/2}_A \otimes I_B)], \phi ^+_{B_0B_i}\right) \\&= \sum _{i=1}^N p(i) F\left( \frac{1}{p(i)}((E^{(i)})^{1/2}_A \otimes I_B) (\rho _{A^NB^N} \otimes \phi ^+_{A_0B_0})\right. \\&\qquad \left. ((E^{(i)})^{1/2}_A \otimes I_B), \phi ^+_{B_0B_i}\otimes \sigma ^{(i)}_{(B_0B_i)^c}\right) \end{aligned}$$

for suitable $\sigma ^{(i)}_{(B_0B_i)^c}$ whose existence is guaranteed by Uhlmann’s Theorem. Here we have introduced $p(i)={{\,\mathrm{tr}\,}}[(E^{(i)})^{1/2}_A (\rho _{A^NB^N} \otimes \tau _{A_0}) (E^{(i)})^{1/2}_A]$. Abbreviating $\sqrt{F}(\cdot ,\cdot ) \equiv \sqrt{F(\cdot ,\cdot )}$, we now have for any $j\in \{1,\ldots ,N\}$ that

$$\begin{aligned} F^*_d(N)&\le \sum _{i=1}^N p(i) \sqrt{F}\left( \frac{1}{p(i)}((E^{(i)})^{1/2}_A \otimes I_B) (\rho _{A^NB^N} \otimes \phi ^+_{A_0B_0})\right. \\&\qquad \left. ((E^{(i)})^{1/2}_A \otimes I_B), \phi ^+_{B_0B_i}\otimes \sigma ^{(i)}_{(B_0B_i)^c}\right) \\&\le \sqrt{F}\left( \rho _{B_j} \otimes \tau _{B_0}, p(j) \phi ^+_{B_0B_j} + (1-p(j)) \tau _{B_0} \otimes \sigma _{B_j}\right) \end{aligned}$$

where the second step uses joint concavity of the root fidelity, and we trace out all systems but $B_0B_j$, with $\sigma _{B_j}$ being some appropriate state. Now, the fact that $\langle \phi |^+_ {AB} \bigl (X_A \otimes \tau _B\bigr )|\phi \rangle ^+_ {AB} = \frac{1}{d^2}{{\,\mathrm{tr}\,}}(X_A)$ for any operator $X_A$ and data processing inequality with respect to the binary measurement $\{\phi ^+_{B_0B_j}, I-\phi ^+_{B_0B_j}\}$ gives

$$\begin{aligned} F^*_d(N)&\le \sqrt{f}\left( \frac{1}{d^2}, p(j) + (1-p(j)) \frac{1}{d^2}\right) , \end{aligned}$$

where $\sqrt{f}(x,y)=\sqrt{xy}+\sqrt{(1-x)(1-y)}$ is the binary root fidelity. Note that $f(q,p + (1-p)q)$ is monotonically increasing as *p* decreases from 1 to 0. Now, one of the *N* probabilities *p*(*j*) has to be $\ge 1/N$. Thus,

7.6 $$\begin{aligned} F^*_d(N)&\le \sqrt{f}\left( \frac{1}{d^2}, \frac{1}{N} + \left( 1-\frac{1}{N} \right) \frac{1}{d^2}\right) . \end{aligned}$$

To derive the non-asymptotic bound (7.4), Equation (7.6) can be rearranged as

$$\begin{aligned} F^*_d(N)&\le \frac{1}{d^2}\left[ \left( d^2-1\right) \left( 1-\frac{1}{2 N}\right) \sqrt{1-\frac{1}{(1-2 N)^2}} +\left( \frac{d^2-1}{2 N}+1\right) \right. \\&\quad \left. \sqrt{1-\frac{\left( d^2-1\right) ^2}{\left( d^2+2 N-1\right) ^2}}\right] . \end{aligned}$$

We bound the square roots using $\sqrt{1+a}\le 1+a/2$ for any $a\ge -1$ to obtain

$$\begin{aligned} F^*_d(N)&\le \frac{1}{d^2}\left[ \left( d^2-1\right) \left( 1-\frac{1}{2 N}\right) \left( 1-\frac{1}{2 (1-2 N)^2}\right) +\left( \frac{d^2-1}{2 N}+1\right) \right. \\&\qquad \left. \left( 1-\frac{\left( d^2-1\right) ^2}{2 \left( d^2+2 N-1\right) ^2}\right) \right] \\&=1-\frac{d^2-1}{8N^2}\frac{1}{(1-\frac{1}{2N}) \left( 1+\frac{d^2-1}{2N}\right) }\\&\le 1-\frac{d^2-1}{8N^2}\frac{1}{1+\frac{d^2-2}{2N}}, \end{aligned}$$

which is (7.4). For $N\rightarrow \infty$ this implies

$$\begin{aligned} F^*_d(N)&\le 1 - \frac{d^2-1}{8} \frac{1}{N^2} + O(N^{-3}), \end{aligned}$$

which is (7.5) and concludes the proof. $\quad \square$

**Fig. 4:**
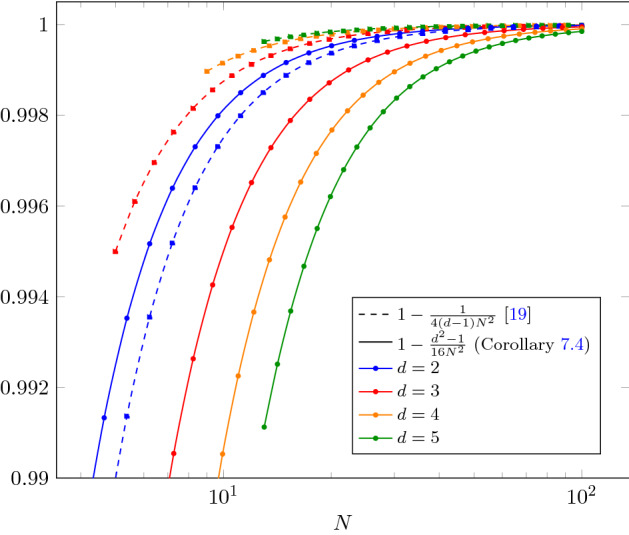
Comparison of the converse bound $F_d^*(N) \le 1-\frac{1}{4(d-1)N^2}$ derived in [19] and the converse bound $F_d^*(N)\le 1-\frac{d^2-1}{16N^2}$ derived in Corollary 7.4, valid for $N>d^2/2$

Combining Theorem 7.5 with Corollary 7.4 above yields a simplified bound as a corollary, that we stated as Corollary 1.6 in Sect. 1.2 as one of our main results. We restate it below for convenience, and in Fig. 4 we compare the quality of this bound for $N>d^2/2$ with the converse bound (3.4) derived in [19].

### Corollary 1.6

(Restated). For a general port-based teleportation scheme with input dimension *d* and *N* ports, the entanglement fidelity $F_d^*$ and the diamond norm error $\varepsilon _d^*$ can be bounded as

$$\begin{aligned} F_d^*(N)&\le {\left\{ \begin{array}{ll} \frac{\sqrt{N}}{d}&{}\quad \text { if } N\le \frac{d^2}{2}\\ 1-\frac{d^2-1}{16N^2}&{}\quad \text { otherwise} \end{array}\right. }&\varepsilon _d^*(N)\ge {\left\{ \begin{array}{ll} 2\bigl (1-\frac{\sqrt{N}}{d}\bigr )&{}\quad \text { if } N\le \frac{d^2}{2}\\ 2\frac{d^2-1}{16N^2}&{}\quad \text { otherwise.} \end{array}\right. } \end{aligned}$$

## Conclusion

In this paper, we completed the picture of the asymptotic performance of port-based teleportation (PBT) in the important regime when the input dimension is fixed while the number of ports tends to infinity. In particular, we determined the asymptotic performance of deterministic PBT in the fully optimized setting, showing that the optimal infidelity decays as $\Theta (1/N^2)$ with the number of ports *N*. We also determined the precise asymptotics of the standard protocol for deterministic PBT (which uses EPR pairs and the ‘pretty good’ measurement) as well as probabilistic PBT using EPR pairs. The asymptotics for probabilistic PBT in the fully optimized setting had been determined previously in [22].

While our work closes a chapter in the study of PBT, it opens several interesting avenues for further investigation, both in the finite and in the asymptotic regime. Note that the limit $d\rightarrow \infty$ for fixed *N* is not very interesting, as the error tends to one in this regime. However, it would be natural to consider limits where both *N* and *d* tend to infinity. In particular, the fidelity $F_d^*(N)$ plausibly has a nontrivial limit when the ratio $N/d^2$ remains fixed. Given the import of PBT to, e.g., instantaneous non-local quantum computation, it would be desirable to determine the limiting value. Finally, we also mention the problem of determining the exact functional dependence on *d* of the leading order coefficient $\lim _{N\rightarrow \infty }N^2(1-F_d^*(N))$ in fully optimized deterministic PBT. Furthermore, we hope that our mathematical tools will be helpful for determining the asymptotics of other quantum information-theoretic tasks that can also be characterized in terms of representation-theoretic data, such as quantum state purification.

## Proof of Lemma 3.6

The following lemma was first derived in [21]. In this section we give an alternative proof. Our proof is elementary and only uses the Schur–Weyl duality and the Pieri rule.

### Lemma 3.6

(Restated) The eigenvalues of the operator

$$\begin{aligned} T(N)_{AB^N} = \frac{1}{N} \left( \phi ^+_{AB_1}\otimes I_{B_1^c} + \ldots + \phi ^+_{AB_N}\otimes I_{B_N^c} \right) \end{aligned}$$

on $(\mathbb {C}^d)^{\otimes (1+N)}$ are given by the numbers

$$\begin{aligned} \frac{1}{dN} \gamma _\mu (\alpha ) = \frac{1}{d} \frac{d_\alpha m_{d,\mu }}{d_\mu m_{d,\alpha }}, \end{aligned}$$

where $\alpha \vdash _{d}N-1$, the Young diagram $\mu \vdash _d N$ is obtained from $\alpha$ by adding a single box, and $\gamma _\mu (\alpha )$ is defined in Eq. (3.10).

### Proof

We note that the operator *T*(*N*) commutes with the action of *U*(*d*) by $\bar{U} \otimes U^{\otimes N}$ as well as with the action of $S_N$ that permutes the systems $B_1,\ldots ,B_N$. Let us work out the corresponding decomposition of $(\mathbb {C}^d)^{1+N}$: We first consider the action of $U(d) \times U(d)$ by $\bar{U} \otimes V^{\otimes N}$ together with the $S_N$. By Schur–Weyl duality,

$$\begin{aligned} (\mathbb {C}^d)^{1+N} \cong (\mathbb {C}^d)^* \otimes \bigoplus _{\mu \vdash _d N} V_\mu ^d \otimes W_\mu . \end{aligned}$$

The notation means that $\mu$ runs over all Young diagrams with *N* boxes and no more than *d* rows (i.e., $\mu _1\ge \ldots \ge \mu _d\ge 0$ and $\sum _j \mu _j=N$). We write $V_\mu ^d$ for the irreducible *U*(*d*)-representation with highest weight $\mu$, and $W_\mu$ for the irreducible $S_N$-representation corresponding to the partition $\mu$.

The dual representation $(\mathbb {C}^d)^*$ is not polynomial; its highest weight is $(-1,0,\ldots ,0)$. However, $(\mathbb {C}^d)^* \cong V_{(1,\ldots ,1,0)}^d \otimes {\det }^{-1}$. The (dual) Pieri rule tells us that $V_{(1,\ldots ,1,0)}^d \otimes V_\mu ^d$ contains all irreducible representations whose highest weight can be obtained by adding 1’s to all but one of the rows (with multiplicity one). Tensoring with the determinant amounts to subtracting $(-1,\ldots ,-1)$, so the result of tensoring with $(\mathbb {C}^d)^*$ amounts to subtracting 1 from one of the rows:

$$\begin{aligned} (\mathbb {C}^d)^* \otimes V_\mu ^d = \bigoplus _{i : \mu _i > \mu _{i+1}} V_{\mu - \epsilon _i}^d, \end{aligned}$$

where we write $\epsilon _i$ for the *i*th standard basis vector. (Note that we set $\mu _{d+1}=-\infty$, so that $i=d$ is always a valid choice in the direct sum above; hence, $\mu -\epsilon _i$ is always a highest weight, but does not need to be a Young diagram.) Thus, we obtain the following multiplicity-free decomposition into $U(d) \times S_N$-representations:

$$\begin{aligned} (\mathbb {C}^d)^{1+N} \cong \bigoplus _{\mu \vdash _d N} \bigoplus _{i: \mu _i > \mu _{i+1}} V_{\mu - \epsilon _i}^d \otimes W_\mu . \end{aligned}$$

The operator *T*(*N*) can be decomposed accordingly:

$$\begin{aligned} T(N) = \bigoplus _{\mu ,i} t_{\mu ,i} \cdot I_{V_{\mu - \epsilon _i}^d} \otimes I_{W_\mu } \end{aligned}$$

for some $t_{\mu ,i}\ge 0$. To determine the $t_{\mu ,i}$, let us denote by $P_\mu$ the isotypical projectors for the $S_N$-action on $(\mathbb {C}^d)^{\otimes N}$ and by $Q_\alpha$ the isotypical projectors for the *U*(*d*) action by ${\bar{U}} \otimes U^{\otimes N}$ (they commute). Then:

A.1 $$\begin{aligned} {{\,\mathrm{tr}\,}}T(N) (I_A \otimes P_\mu ) Q_{\mu - \epsilon _i} = t_{\mu ,i} \, \dim (V_{\mu -\epsilon _i}^d) \, \dim (W_\mu ) \,. \end{aligned}$$

On the other hand:

$$\begin{aligned} {{\,\mathrm{tr}\,}}T(N) (I_A \otimes P_\mu ) Q_{\mu - \epsilon _i} = {{\,\mathrm{tr}\,}}\phi ^+_{AB_1} (I_A \otimes P_\mu ) Q_{\mu - \epsilon _i}. \end{aligned}$$

The maximally entangled state $\phi ^+_{AB_1}$ is invariant under ${\bar{U}} \otimes U$. This means that on the range of the projector $\phi ^+_{AB_1}$, the actions of ${\bar{U}} \otimes U^{\otimes N}$ and $I_{AB_1} \otimes U^{\otimes (N-1)}$ agree! Explicitly:

$$\begin{aligned} \mathbb {C}|\phi ^+\rangle _{AB_1} \otimes (\mathbb {C}^d)^{\otimes (N-1)} \cong \mathbb {C}|\phi ^+\rangle _{AB_1} \otimes \bigoplus _{\alpha \vdash _d N-1} V_{\alpha }^d \otimes [\alpha ] \cong \bigoplus _{\alpha \vdash _d N-1} V_{\alpha }^d \otimes [\alpha ]. \end{aligned}$$

It follows that

$$\begin{aligned} \phi ^+_{AB_1} Q_\alpha = \phi ^+_{AB_1} (I_{AB_1} \otimes Q'_\alpha ) \end{aligned}$$

where $Q'_\alpha$ refers to the action of *U*(*d*) by $U^{\otimes (N-1)}$ on $B_2\ldots {}B_n$, and so

$$\begin{aligned} {{\,\mathrm{tr}\,}}\phi ^+_{AB_1} (I_A \otimes P_\mu ) Q_{\mu - \epsilon _i} = {\left\{ \begin{array}{ll} {{\,\mathrm{tr}\,}}\phi ^+_{AB_1} (I_A \otimes P_\mu ) (I_{AB_1} \otimes Q'_\alpha ) &{}\quad \text {if } \alpha \,{:}{=}\, \mu -\epsilon _i \text { is a partition}, \\ 0 &{}\quad \text {otherwise}. \end{array}\right. } \end{aligned}$$

We can now trace over the A-system:

$$\begin{aligned} {{\,\mathrm{tr}\,}}\phi ^+_{AB_1} (I_A \otimes P_\mu ) (I_{AB_1} \otimes Q'_\alpha ) = \frac{1}{d} {{\,\mathrm{tr}\,}}P_\mu (I_{B_1} \otimes Q'_\alpha ) \,. \end{aligned}$$

The remaining trace is on $(\mathbb {C}^d)^{\otimes N}$. The operator $P_\mu$ refers to the $S_N$-action, while $Q'_\alpha$ refers to the *U*(*d*)-action by $U^{\otimes (N-1)}$ on $B_2\ldots {}B_n$. Equivalently, we can define $Q'_\alpha$ with respect to the $S_{N-1}$ action by permuting the last $N-1$ tensor factors. Using Schur–Weyl duality and the branching rule for restricting $S_N$ to $S_1 \times S_{N-1}$:

$$\begin{aligned} (\mathbb {C}^d)^{\otimes N} = \bigoplus _\mu V_\mu ^d \otimes W_\mu = \bigoplus _\mu V_\mu ^d \otimes \bigoplus _{i : \alpha = \mu - \epsilon _i \text { partition}} W_\alpha \end{aligned}$$

And hence

$$\begin{aligned} \frac{1}{d} {{\,\mathrm{tr}\,}}P_\mu (I_{B_1} \otimes Q'_\alpha ) = \frac{1}{d} \dim (V^d_\mu ) \dim (W_\alpha ) \end{aligned}$$

in the case of interest. Comparing this with Eq. (A.1), we obtain the following result:

$$\begin{aligned} t_{\mu ,i} = \frac{1}{d} \frac{\dim (V^d_\mu )}{\dim (V^d_\alpha )} \frac{\dim (W_\alpha )}{\dim (W_\mu )} \end{aligned}$$

if $\alpha = \mu - \epsilon _i$ is a partition, and otherwise zero. These are the desired eigenvalues of *T*(*N*). $\quad \square$

## A Family of Explicit Protocols for Deterministic PBT

Guessing a good candidate density $c_\mu$ with a simple functional form for the optimization in Eq. (3.6) yields a protocol with performance close to the achievability bound Theorem 1.5.

### Theorem B.1

For fixed but arbitrary dimension *d*, there exists a concrete protocol for deterministic PBT with entanglement fidelity

$$\begin{aligned} F\ge 1-\frac{d^4(d+3)}{2N^{2}}-O(N^{-3}) \end{aligned}$$

### Proof

Assume that $N/d^2$ is an integer (otherwise use only the first $d^2\left\lfloor \frac{N}{d^2}\right\rfloor$ ports). Let $c_\mu$ be defined such that

$$\begin{aligned} q(\mu )=c_\mu p(\mu )={\left\{ \begin{array}{ll} \eta _{N}\left( R^2-r(\mu )^2\right) ^2 &{}\quad r\le R\\ 0&{}\quad \text {else}, \end{array}\right. } \end{aligned}$$

with

$$\begin{aligned} r(\mu )&=\Vert \mu -\hat{\mu }\Vert _2,\\ \hat{\mu }&=\left( (2d-1)\frac{N}{d^2},(2d-3)\frac{N}{d^2},\ldots ,\frac{N}{d^2}\right) ,\\ R&=\sqrt{2}\frac{N}{d^2}, \end{aligned}$$

and

$$\begin{aligned} \eta _{N}=\left( \sum _{\begin{array}{c} \mu \in \hat{\mu }+\Lambda _d\\ r(\mu )\le R \end{array}}\left( R^2-r(\mu )^2\right) ^2\right) ^{-1} \end{aligned}$$

is a normalization factor that ensures that *q* is a probability distribution. $\hat{\mu }$ has Euclidean distance *R* from the boundary of the set of Young diagrams, i.e. all vectors $\mu \in \hat{\mu }+\Lambda _d$ such that $\Vert \mu -\hat{\mu }\Vert _2\le R$ are Young diagrams. We extend the probability distribution *q* to be defined on all $v\in \hat{\mu }+\Lambda _d$ for convenience. Let $B^{\Lambda _d}_L(v_0)=\{v\in v_0+\Lambda _d|\Vert v-v_0\Vert _2\le L\}$. We now look at the PBT-fidelity for the protocol using the density $c_\mu$. First note that the formula Eq. (3.6) can be rearranged in the following way,

$$\begin{aligned} d^2F&=\sum _{\alpha \vdash _d N-1}\left( \sum _{\mu =\alpha +\square }\sqrt{q(\mu )}\right) ^2\\&=\sum _{\alpha \vdash _d N-1}\sum _{\mu ,\mu '=\alpha +\square }\sqrt{q(\mu )q(\mu ')}\\&=\sum _{\mu \vdash _d N}\sum _{\mu '=\mu +\square -\square }\sqrt{q(\mu )q(\mu ')}. \end{aligned}$$

In the last line, the notation $\mu '=\mu +\square -\square$ means summing over all possibilities to remove a square from $\mu$ and adding one, including removing and adding the same square. Noting that all vectors in $B^{\Lambda _d}_R(\hat{\mu })$ are Young diagrams, we can write

B.1 $$\begin{aligned} d^2F&=\sum _{\mu \in B^{\Lambda _d}_R(\hat{\mu })}\sum _{i,j=1}^d\mathbb {1}_{B_R(\hat{\mu })}(\mu +e_i-e_j)\sqrt{q(\mu )q(\mu +e_i-e_j)}\nonumber \\&=\sum _{\mu \in B^{\Lambda _d}_R(\hat{\mu })}q(\mu )\sum _{i,j=1}^d\mathbb {1}_{B_R(\hat{\mu })}(\mu +e_i-e_j)\sqrt{\frac{q(\mu +e_i-e_j)}{q(\mu )}}\nonumber \\&=\sum _{\mu \in B^{\Lambda _d}_R(\hat{\mu })}q(\mu )\sum _{i,j=1}^d\mathbb {1}_{B_R(\hat{\mu })}(\mu +e_i-e_j)\left( 1+2\frac{g_{ij}(\mu )-1}{\sqrt{f(\mu )}}\right) \nonumber \\&=\sum _{\mu \in B^{\Lambda _d}_{R-\sqrt{2}}(\hat{\mu })}q(\mu )\sum _{i,j=1}^d\left( 1+2\frac{g_{ij}(\mu )-1}{\sqrt{f(\mu )}}\right) \nonumber \\&\quad +\sum _{\mu \in B^{\Lambda _d}_{R}(\hat{\mu }){\setminus } B^{\Lambda _d}_{R-\sqrt{2}}(\hat{\mu })}q(\mu )\sum _{i,j=1}^d\mathbb {1}_{B_R(\hat{\mu })}(\mu +e_i-e_j)\left( 1+2\frac{g_{ij}(\mu )-1}{\sqrt{f(\mu )}}\right) . \end{aligned}$$

Here we have defined the functions

$$\begin{aligned} g_{ij}(\mu )=\mu _j-\hat{\mu }_j-\mu _i+\hat{\mu }_i \end{aligned}$$

and

$$\begin{aligned} f(\mu )=\left( R^2-r(\mu )^2\right) ^2. \end{aligned}$$

The last equation holds because $\Vert e_i-e_j\Vert _2=(1-\delta _{ij})\sqrt{2}$, i.e. for all $\mu \in B^{\Lambda _d}_{R-\sqrt{2}}(\hat{\mu })$ and all $1\le i,j\le d$ we have $\mu +e_i-e_j\in B^{\Lambda _d}_{R}(\hat{\mu })$.

We can bound the normalization constant as follows. Denote by $\mathcal {P}(\Lambda _d)$ the unit cell of $\Lambda _d$ with smallest diameter, $\ell$ . The volume of the unit cell is $\sqrt{d}$, which can be seen as follows.^8^ A basis for the lattice $\Lambda _d = \lbrace v\in \mathbb {Z}^d:\sum _{i=1}^d v_i = 0\rbrace$ is given by $B=\lbrace b_i\rbrace _{i=1}^{d-1}$, where $b_i=e_1-e_{i+1}$. It follows that $M=B^T B$ is a $(d-1)\times (d-1)$-matrix with all diagonal elements equal to 2 and all off-diagonal elements equal to 1. The matrix *M* has one eigenvalue *d* corresponding to the eigenvector $\sum _{i=1}^{d-1} e_i$, and $d-2$ eigenvalues 1 corresponding to the eigenvectors $e_1-e_{i+1}$, respectively. Hence, $\det (\Lambda _d) = \sqrt{d}$.

Let further $g:\mathbb {R}\Lambda _d\rightarrow \Lambda _d$ be the function such that for all $x\in \mathbb {R}\Lambda ^d$ there exist $\gamma _i\in (-1/2,1/2]$, $i=1,\ldots ,d-1$ such that

$$\begin{aligned} x=g_N(x)+\sum _{i=1}^{d-1}\gamma _i a_i. \end{aligned}$$

Heuristically, *g* is the function that maps every point in the $(d-1)$-dimensional subspace $\Lambda ^d$ lives in to the lattice point *v* in whose surrounding unit cell it lies, where the surrounding unit cell is here the set $\{v+\sum _{i=1}^{d-1}\gamma _i a_i|\gamma _i\in (-1/2,1/2]\}$, i.e. the point lies in the center of the cell. As *f* is nonnegative, we have with *l* as defined above that

B.2 $$\begin{aligned} \eta _{N}^{-1}&=\sum _{\mu \in B^{\Lambda _d}_R(\hat{\mu })}f(\mu )\nonumber \\&\le \frac{1}{\sqrt{d}} \int _{ B^{\Lambda _d}_{R+\ell /2}(\hat{\mu })}f(g_N(x))\mathrm {d}x\nonumber \\&\le \frac{1}{\sqrt{d}} \int _{ B^{\Lambda _d}_{R+\ell /2}(\hat{\mu })}f(x)\mathrm {d}x\nonumber \\&\quad +\frac{1}{\sqrt{d}} \int _{ B^{\Lambda _d}_{R+\ell /2}(\hat{\mu })}\frac{\ell }{2}\max _{x':\Vert x-x'\Vert _2\le \ell /2}\left\| (\nabla f)(x')\right\| _2\mathrm {d}x \end{aligned}$$

The gradient of *f* is given by

$$\begin{aligned} (\nabla f)(x)=-4(R^2-\Vert x\Vert _2^2)x. \end{aligned}$$

We can bound

$$\begin{aligned} |4(R^2-(r\pm l)^2)(r\pm l)|\le 4(R^2-(r-l)^2)(r+l), \end{aligned}$$

so

$$\begin{aligned}&\frac{1}{\sqrt{d}} \int _{ B^{\Lambda _d}_{R+\ell /2}(\hat{\mu })}\frac{\ell }{2}\max _{x':\Vert x-x'\Vert _2\le \ell /2}\left\| (\nabla f)(x')\right\| _2\mathrm {d}x\\&\quad \le \frac{1}{\sqrt{d}} \int _{ B^{\Lambda _d}_{R+\ell /2}(\hat{\mu })}\frac{\ell }{2}4(R^2-(r(x)-\ell /2)^2)(r(x)+\ell /2)\mathrm {d}x\\&\quad = \frac{2\ell {{\,\mathrm{vol}\,}}(\mathbb {S}_{d-2})}{\sqrt{d}} \int _{ 0}^{R+\ell /2}r^{d-2}(R^2-(r-\ell /2)^2)(r+\ell /2)\mathrm {d}r\\&\quad = \frac{2\ell {{\,\mathrm{vol}\,}}(\mathbb {S}_{d-2})}{\sqrt{d}}\left( \frac{1}{d}-\frac{1}{d+2}\right) \left( R+\ell /2\right) ^{d+2}+O(R^{d+1}). \end{aligned}$$

Here, we changed into spherical coordinates with origin in $\hat{\mu }$ in the third line, and ${{\,\mathrm{vol}\,}}(\mathbb {S}_{d-2})$ is the volume of the $(d-2)$-dimensional sphere. Turning to the first term in Eq. (B.2), we calculate

$$\begin{aligned} \int _{ B^{\Lambda _d}_{R+\ell /2}(\hat{\mu })}f(x)\mathrm {d}x&=\int _{ B^{\Lambda _d}_{R+\ell /2}(\hat{\mu })}(R^2-r(x)^2)^2\mathrm {d}x\\&={{\,\mathrm{vol}\,}}(\mathbb {S}_{d-2})\int _{ 0}^{R+\ell /2}r^{d-2}(R^2-r^2)^2\mathrm {d}r\\&={{\,\mathrm{vol}\,}}(\mathbb {S}_{d-2})\left( R+\ell /2\right) ^{d+3}\left( \frac{1}{d-1}-\frac{2}{d+1}+\frac{1}{d+3}\right) \\&=\frac{8{{\,\mathrm{vol}\,}}(\mathbb {S}_{d-2})\left( R+\ell /2\right) ^{d+3}}{d^3+3d^2-d-3}. \end{aligned}$$

Combining the last two equations, expanding the polynomials of the form $\left( R+\ell /2\right) ^{k}$ and using the power series expansion of $1/(1+x)$ we finally arrive at

$$\begin{aligned} \eta _{N}&=\frac{\sqrt{d}\left( d^3+3d^2-d-3\right) }{8{{\,\mathrm{vol}\,}}(\mathbb {S}_{d-2})} R^{-(d+3)}+O(R^{-(d+4)}). \end{aligned}$$

Returning to equation Eq. (B.1), let us first bound the magnitude of the last term. To this end, observe that for $r(\mu )\ge R-\sqrt{2}$, we have

$$\begin{aligned} \sqrt{f(\mu )}&=(R^2-r(\mu )^2)\\&\le 2\sqrt{2} R. \end{aligned}$$

Furthermore we have that

$$\begin{aligned} \mathbb {1}_{B_R(\hat{\mu })}(\mu +e_i-e_j)\left( 1+2\frac{g_{ij}(\mu )-1}{\sqrt{f(\mu )}} \right)&\le 1+2\frac{2R}{\sqrt{f(\mu )}}, \end{aligned}$$

and hence

$$\begin{aligned}&\sum _{\mu \in B^{\Lambda _d}_{R}(\hat{\mu }){\setminus } B^{\Lambda _d}_{R-\sqrt{2}}(\hat{\mu })}q(\mu )\sum _{i,j=1}^d\mathbb {1}_{B_R(\hat{\mu })}(\mu +e_i-e_j)\left( 1+2\frac{g_{ij}(\mu )-1}{\sqrt{f(\mu )}}\right) \\&\quad \le d^2\eta _{N}\sum _{\mu \in B^{\Lambda _d}_{R}(\hat{\mu }){\setminus } B^{\Lambda _d}_{R-\sqrt{2}}(\hat{\mu })}\left( f(\mu )+2\sqrt{f(\mu )}R\right) \\&\quad \le d^2\eta _{N}\sum _{\mu \in B^{\Lambda _d}_{R}(\hat{\mu }){\setminus } B^{\Lambda _d}_{R-\sqrt{2}}(\hat{\mu })}\left( \left( 2\sqrt{2}R\right) ^2+4\sqrt{2} R^2\right) \\&\quad \le 4(2+\sqrt{2})d^2R^2\eta _{N}\Bigl |B^{\Lambda _d}_{R}(\hat{\mu }){\setminus } B^{\Lambda _d}_{R-\sqrt{2}}(\hat{\mu })\Bigr |. \end{aligned}$$

To bound the number of lattice points in the spherical shell $B^{\Lambda _d}_{R}(\hat{\mu }){\setminus } B^{\Lambda _d}_{R-\sqrt{2}}(\hat{\mu })$, note that i) each lattice point is surrounded by its own unit cell, and ii) these cells have diameter $\ell$. Therefore all these unit cells are disjoint subsets of a shell of width $\sqrt{2}+\ell$, and hence we have the bound

$$\begin{aligned} \Bigl |B^{\Lambda _d}_{R}(\hat{\mu }){\setminus } B^{\Lambda _d}_{R-\sqrt{2}}(\hat{\mu })\Bigr |\le {{\,\mathrm{vol}\,}}{\mathbb {S}_{d-2}}(R+\ell )^{d-2}(\ell +\sqrt{2}). \end{aligned}$$

Combining the bounds we arrive at

$$\begin{aligned}&\sum _{\mu \in B^{\Lambda _d}_{R}(\hat{\mu }){\setminus } B^{\Lambda _d}_{R-\sqrt{2}}(\hat{\mu })}q(\mu )\sum _{i,j=1}^d\mathbb {1}_{B_R(\hat{\mu })}(\mu +e_i-e_j)\left( 1+2\frac{g_{ij}(\mu )-1}{\sqrt{f(\mu )}}\right) =O(R^{-3}) \end{aligned}$$

Turning to the first expression on the right hand side of Eq. (B.1), we observe that both the set $B^{\Lambda _d}_{R-\sqrt{2}}$ and the distribution *q* are invariant under the map $\mu \mapsto 2\mu -\hat{\mu }$, i.e. central reflection about $\hat{\mu }$. Therefore the sum over $g_{ij}(\mu )$, which is linear in $\mu -\hat{\mu }$, vanishes, i.e.

$$\begin{aligned}&\sum _{\mu \in B^{\Lambda _d}_{R-\sqrt{2}}(\hat{\mu })}q(\mu )\sum _{i,j=1}^d\left( 1+2\frac{g_{ij}(\mu )-1}{\sqrt{f(\mu )}}\right) \\&\quad =\sum _{\mu \in B^{\Lambda _d}_{R-\sqrt{2}}(\hat{\mu })}q(\mu )\sum _{i,j=1}^d\left( 1-2\frac{1}{\sqrt{f(\mu )}}\right) \\&\quad =\sum _{\mu \in B^{\Lambda _d}_{R}(\hat{\mu })}q(\mu )\sum _{i,j=1}^d\left( 1-2\frac{1}{\sqrt{f(\mu )}}\right) -\sum _{\mu \in B^{\Lambda _d}_{R}(\hat{\mu }){\setminus } B^{\Lambda _d}_{R-\sqrt{2}}(\hat{\mu })}q(\mu )\sum _{i,j=1}^d\left( 1-2\frac{1}{\sqrt{f(\mu )}}\right) \\&\quad \ge d^2-\eta _{N}\left( 2\sum _{\mu \in B^{\Lambda _d}_{R}(\hat{\mu })}\sqrt{f(\mu )}+\sum _{\mu \in B^{\Lambda _d}_{R}(\hat{\mu }){\setminus } B^{\Lambda _d}_{R-\sqrt{2}}(\hat{\mu })}\sqrt{f(\mu )}\left( \sqrt{f(\mu )}-2\right) \right) \end{aligned}$$

Using the same argument as for bounding $\eta _{N}$, we find

$$\begin{aligned} \sum _{\mu \in B^{\Lambda _d}_{R}(\hat{\mu })}\sqrt{f(\mu )}&\le \frac{2\ell {{\,\mathrm{vol}\,}}(\mathbb {S}_{d-2})}{\sqrt{d}} \int _{ 0}^{R+\ell /2}r^{d-2}(R^2-(r-\ell /2)^2)\mathrm {d}r\\&=\frac{{{\,\mathrm{vol}\,}}(\mathbb {S}_{d-2})R^{d+1}}{(d^2-1)\sqrt{d}}+O(R^d) . \end{aligned}$$

The second term is bounded in the same way as the spherical shell sum above, yielding

$$\begin{aligned} \eta _{N}d^2\sum _{\mu \in B^{\Lambda _d}_{R}(\hat{\mu }){\setminus } B^{\Lambda _d}_{R-\sqrt{2}}(\hat{\mu })}\sqrt{f(\mu )}\left( \sqrt{f(\mu )}-2\right) =O(R^{-3}). \end{aligned}$$

Combining all bounds, we arrive at

$$\begin{aligned} F&\ge 1-\frac{d^3+3d^2-d-3}{d^2-1}R^{-2}+O(R^{-3})=(d+3)R^{-2}+O(R^{-3}). \end{aligned}$$

Using $R=\frac{N}{d^2}$ we obtain the final bound

$$\begin{aligned} F&\ge 1- \frac{d^4(d+3)}{2N^{2}}+O(R^{-3}). \end{aligned}$$

$$\square $$

## The Maximal Eigenvalue of a $2\times 2$ GUE${}_0$ Matrix

The maximal eigenvalue $\lambda _{\max }(\mathbf {G})$ of a $2\times 2$ GUE${}_0$ matrix $\mathbf {G}$ can be easily analyzed, as $\lambda _{\max }(\mathbf {G})=\sqrt{\frac{1}{2} {{\,\mathrm{tr}\,}}\mathbf {G}^2}$.

### Lemma C.1

For $\mathbf {X}\sim \mathrm {GUE}_0(2)$, $\sqrt{2}\lambda _{\max }(\mathbf {G})\sim \chi _3$, where $\chi _3$ is the chi-distribution with three degrees of freedom.^9^ Consequently, $\mathbb {E}\left[ \lambda _{\max }(\mathbf {G})\right] =\frac{2}{\sqrt{\pi }}$.

### Proof

By definition, the probability density of $\mathrm {GUE}(d)$ is

$$\begin{aligned} p_{\mathrm {GUE}}(M)=\left( 2\pi \right) ^{-\frac{d^2}{2}}\exp \left( -\frac{{{\,\mathrm{tr}\,}}M^2}{2}\right) , \end{aligned}$$

and therefore we get

$$\begin{aligned} p_{\mathrm {GUE}_0}(G)=\left( 2\pi \right) ^{-\frac{d^2-1}{2}}\exp \left( -\frac{{{\,\mathrm{tr}\,}}G^2}{2}\right) , \end{aligned}$$

for the density of GUE${}_0$. Writing $\mathbf {G}=\sum _{i=1}^3\mathbf {x}_i \sigma _i$ with the Pauli matrices $\sigma _i, i=1,2,3$, we see that the $\mathbf {x}_i$ are independent normal random variables with variance 1/2, and

$$\begin{aligned} \lambda _{\max }(\mathbf {G})=\sqrt{\frac{{{\,\mathrm{tr}\,}}\mathbf {G}^2}{2}}=\sqrt{\sum _{i=1}^3\mathbf {x}_i^2 }, \end{aligned}$$

proving the claim. $\quad \square$

## Technical Lemmas

The following “mirror lemma”, also called “transpose trick”, is well known in the literature, and can be proven in a straightforward way:

### Lemma D.1

(Mirror lemma, transpose trick). Let $\lbrace |i\rangle \rbrace _{i=1}^d$ be a basis and $|\gamma \rangle =\sum _{i=1}^d |i\rangle |i\rangle$ be the unnormalized maximally entangled state. For any operator *X*,

$$\begin{aligned} I\otimes X|\gamma \rangle = X^T\otimes I |\gamma \rangle , \end{aligned}$$

where $X^T$ denotes transposition of *X* with respect to the basis $\lbrace |i\rangle \rbrace _{i=1}^d$.

The maximization in the definition of the diamond norm can be carried out explicitly for the distance of two unitarily covariant channels. This is the statement of the following lemma, which is a special case of a more general result about generalized divergences proven in [69].

### Lemma D.2

[69]. Let $\Lambda ^{(i)}_{A\rightarrow A}$ for $i=1,2$ be unitarily covariant maps. Then the maximally entangled state $|\phi ^+\rangle _{AA'}$ is a maximizer for their diamond norm distance, i.e.,

$$\begin{aligned} \left\| \Lambda ^{(1)}_{A\rightarrow A}-\Lambda ^{(2)}_{A\rightarrow A}\right\| _\diamond =\left\| \left( \Lambda ^{(1)}_{A\rightarrow A}-\Lambda ^{(2)}_{A\rightarrow A}\right) (\phi ^+_{AA'})\right\| _1. \end{aligned}$$

The following Lemma from Ref. [5] shows that the entanglement fidelity and the diamond norm distance to the identity channel are even in a 1-1 relation for unitarily covariant channels.

### Lemma D.3

[5]. For a unitarily covariant channel $\Lambda :A\rightarrow A$,

$$\begin{aligned} \Vert {{\,\mathrm{id}\,}}_A-\Lambda \Vert _\diamond =2\left( 1-\sqrt{F}(\Lambda )\right) . \end{aligned}$$

We need an explicit limit of certain Riemann sums. The proof of the following can, e.g., be found in [70].

### Lemma D.4

Let $f:\mathbb {R}_+\rightarrow \mathbb {R}_+$ be nonincreasing such that the (proper or improper) Riemann integral

$$\begin{aligned} \intop _a^b f(x)\mathrm {d}x \end{aligned}$$

exists for all $a,b\in [0,\infty ]$ with $a<b$. Then

$$\begin{aligned} \lim _{n\rightarrow \infty }\frac{1}{n}\sum _{i=1}^{g n}f\left( \frac{c+i}{n}\right) =\intop _0^gf(x)\mathrm {d}x \end{aligned}$$

for all $c\ge 0$ and $g\in [0,\infty ]$.

The following lemma provides the volume of the simplex of ordered probability distributions as well as the volume of its boundary.

### Lemma D.5

Let

$$\begin{aligned} \mathrm {OS}_{d-1}=\left\{ x\in \mathbb {R}^{d}\Bigg |\sum _i x_i=0, x_i\ge x_{i+1}, x_{d}\ge 0\right\} \end{aligned}$$

be the simplex of ordered probability distributions. The volume of this simplex, and the volume of its boundary, are given by

D.1 $$\begin{aligned} {{\,\mathrm{vol}\,}}(\mathrm {OS}_{d-1})&=\frac{1}{\sqrt{d} ((d-1)!)^2}\text {, and}\nonumber \\ {{\,\mathrm{vol}\,}}(\partial \mathrm {OS}_{d-1})&={{\,\mathrm{vol}\,}}(\mathrm {OS}_{d-1})\left( \frac{d(d-1)^2}{\sqrt{2}}+\sqrt{d}(d-1)^{3/2}+\sqrt{2}(d-1)\right) , \end{aligned}$$

respectively.

### Proof

$\mathrm {OS}_{d-1}$ is given in its dual description above, let us therefore begin by finding its extremal points. These are clearly given by

$$\begin{aligned} v_i=\left( \frac{1}{i},\ldots ,\frac{1}{i},0,\ldots ,0\right) , \end{aligned}$$

i.e. the *i*th extremal point has *i* entries $\frac{1}{i}$ and $d-i$ entries 0. The supporting (affine) hyperplanes $H_i$ of the facets $F_i$, $i=1,\ldots ,d$ of $\mathrm {OS}_{d-1}$ in $V_0^{(d-1)}=\left\{ x\in \mathbb {R}^d|\sum _i x_i=0\right\}$ are given by the normalized normal vectors

$$\begin{aligned} n_i&=\frac{e_i-e_{i+1}}{\sqrt{2}},\ i=1,\ldots ,d-1,\text { and}\\ n_d&=\frac{1}{\sqrt{d(d-1)}}(1,\ldots ,1,-d+1). \end{aligned}$$

Now note that the facet $F_d=\{x\in \mathrm {OS}_{d-1}|x_d=0\}$ is equal to $\mathrm {OS}_{d-2}$, and the volume of a $(d-1)$-dimensional pyramid is given by the product of the volume of its base and its height, divided by $d-1$. Therefore we get the recursive formula

$$\begin{aligned} {{\,\mathrm{vol}\,}}(\mathrm {OS}_{d-1})=\frac{1}{d-1}{{\,\mathrm{vol}\,}}(\mathrm {OS}_{d-2})h_{d}, \end{aligned}$$

where we have defied the distance $h_{i}$ between $v_i$ and $H_i$. Let us calculate $h_d$. This can be done by taking the difference of $v_d$ and any point in $H_i$ and calculating the absolute value of its inner product with $n_d$. We thus get

$$\begin{aligned} h_d&=|\langle n_d,v_d-v_1\rangle |\\&=\frac{1}{\sqrt{d(d-1)}}\left| -\frac{d-1}{d}+(d-2)\frac{1}{d}-\frac{d-1}{d}\right| \\&=\frac{1}{\sqrt{d(d-1)}}. \end{aligned}$$

The recursion therefore becomes

$$\begin{aligned} {{\,\mathrm{vol}\,}}(\mathrm {OS}_{d-1})=\sqrt{\frac{d-1}{d}}\frac{1}{(d-1)^{2}}{{\,\mathrm{vol}\,}}(\mathrm {OS}_{d-2}). \end{aligned}$$

The claimed formula for the volume is now proven by induction. $\mathrm {OS}_2$ is just the line from (1, 0) to (1/2, 1/2), so its volume is clearly

$$\begin{aligned} {{\,\mathrm{vol}\,}}(\mathrm {OS}_2)=\frac{1}{\sqrt{2}}=\frac{1}{\sqrt{2}(1!)^2}, \end{aligned}$$

proving Eq. (D.1) for $d=2$. For the induction step, assume that the formula Eq. (D.1) holds for $d=k-1$. Then we have

$$\begin{aligned} {{\,\mathrm{vol}\,}}(\mathrm {OS}_{k-1})&=\sqrt{\frac{k-1}{k}}\frac{1}{(k-1)^{2}}{{\,\mathrm{vol}\,}}(\mathrm {OS}_{k-2})\\&=\sqrt{\frac{k-1}{k}}\frac{1}{(k-1)^{2}}\frac{1}{\sqrt{k-1}((k-2)!)^2}\\&=\frac{1}{\sqrt{k}((k-1)!)^2}.\\ \end{aligned}$$

For the boundary volume, we can use the pyramid volume formula again to obtain

$$\begin{aligned} {{\,\mathrm{vol}\,}}(\mathrm {OS}_{d-1})=\frac{1}{d-1}{{\,\mathrm{vol}\,}}(F_i)h_{i}, \end{aligned}$$

i.e. we obtain the formula

$$\begin{aligned} {{\,\mathrm{vol}\,}}(\partial \mathrm {OS}_{d-1})&=\sum _{i=1}^d{{\,\mathrm{vol}\,}}(F_i)\\&=(d-1){{\,\mathrm{vol}\,}}(\mathrm {OS}_{d-1})\sum _{i=1}^d\frac{1}{h_i}. \end{aligned}$$

We calculate the heights $h_i$ for $i\ne d$. For $1<i<d$ we get in the same way as above for $i=d$,

$$\begin{aligned} h_i&=|\langle n_i,v_i-v_1\rangle |\\&=\frac{1}{i\sqrt{2}}. \end{aligned}$$

for $i=1$ we calculate

$$\begin{aligned} h_1&=|\langle n_1,v_1-v_2\rangle |\\&=\frac{1}{2\sqrt{2}}. \end{aligned}$$

Therefore we get the boundary volume

$$\begin{aligned} {{\,\mathrm{vol}\,}}(\partial \mathrm {OS}_{d-1})&=(d-1){{\,\mathrm{vol}\,}}(\mathrm {OS}_{d-1})\left( 2\sqrt{2}+\sqrt{d(d-1)}+\sqrt{2}\sum _{i=2}^{d-1}i\right) \\&=(d-1){{\,\mathrm{vol}\,}}(\mathrm {OS}_{d-1})\left( \sqrt{2}+\sqrt{d(d-1)}+\frac{d(d-1)}{\sqrt{2}}\right) \\&={{\,\mathrm{vol}\,}}(\mathrm {OS}_{d-1})\left( \frac{d(d-1)^2}{\sqrt{2}}+\sqrt{d}(d-1)^{3/2}+\sqrt{2}(d-1)\right) . \end{aligned}$$

## Footnotes

1 The standard protocol uses a maximally entangled resource state and the so-called Pretty Good Measurement.

2 This can even be done while keeping the structure of the resource state if desired, by adding the purifying system to one of Alice’s systems $A_i$, or splitting it between all of them.

3 Alternatively, one could call “deterministic PBT” just “PBT” and for “probabilistic PBT” use the term “heralded PBT”, which is borrowed from quantum optics terminology as used in, e.g., [42]. However, we will stick to the widely used terms.

4 Here, $f(n) = \omega (g(n))$ means that |*f*(*n*)/*g*(*n*)| diverges as $n\rightarrow \infty$.

5 The second derivative is continuous and its limit for the argument approaching the boundary exists.

6 Observe that a constant fraction of $U_N(y)$ of each lattice point $y\in \mathrm {OS}_{d-1}\cap \frac{1}{N}\mathbb {Z}^d$ lies inside $\mathrm {OS}_{d-1}$. This fraction is not uniformly bounded in *d*, as the solid angle of the vertices of $\mathrm {OS}_{d-1}$ decreases with *d*. However, this does not concern us, since we are only interested in the limit $N\rightarrow \infty$ for fixed *d*.

7 Except in the PhD thesis of one of the authors [66].
